# Do Laboratory Results Concerning High-Viscosity Glass-Ionomers versus Amalgam for Tooth Restorations Indicate Similar Effect Direction and Magnitude than that of Controlled Clinical Trials? - A Meta-Epidemiological Study

**DOI:** 10.1371/journal.pone.0132246

**Published:** 2015-07-13

**Authors:** Steffen Mickenautsch, Veerasamy Yengopal

**Affiliations:** Systematic Review initiative for Evidence-based Minimum Intervention in Dentistry, Department of Community Dentistry, Faculty of Health Sciences, University of the Witwatersrand, Johannesburg, South Africa; University of North Carolina at Chapel Hill, UNITED STATES

## Abstract

**Background:**

A large percentage of evidence concerning dental interventions is based on laboratory research. The apparent wealth of laboratory evidence is sometimes used as basis for clinical inference and recommendations for daily dental practice. In this study two null-hypotheses are tested: whether trial results from laboratory and controlled clinical trials concerning the comparison of high-viscosity glass-ionomer cements (HVGIC) to amalgam for restorations placed in permanent posterior teeth have: (i) similar effect direction and (ii) similar effect magnitude.

**Methods:**

7 electronic databases were searched, as well as reference lists. Odds ratios (OR) and Standardised Mean Differences (SMD) with 95% Confidence intervals were computed for extracted dichotomous and continuous data, respectively. Pooled effect estimates for laboratory and clinical data were computed to test for effect direction. Odds ratios were converted into SMDs. SMDs from laboratory and clinical data were statistically compared to test for differences in effect magnitude. The analysed results were further investigated within the context of potential influencing or confounding factors using a Directed acyclic graph.

**Results:**

Of the accepted eight laboratory and nine clinical trials, 13 and 21 datasets could be extracted, respectively. The pooled results of the laboratory datasets were highly statistically significant in favor of amalgam. No statistically significant differences, between HVGICs and amalgam, were identified for clinical data. For effect magnitude, statistically significant differences between clinical and laboratory trial results were found. Both null-hypotheses were rejected.

**Conclusion:**

Laboratory results concerning high-viscosity glass-ionomers versus amalgam for tooth restorations do not indicate similar effect direction and magnitude than that of controlled clinical trials.

## Introduction

A large percentage of evidence concerning dental interventions is based on laboratory research. A simple PubMed search (27 August 2014) of the dental literature published between 2009–2014, using the broad MeSH terms “Dental Amalgam” and “Glass Ionomer Cements” (S1 File) reveals a 2–3, as well as an over 7 times higher number of listed citations of laboratory (including *in-vitro* and animal based *in-vivo*) studies than of prospective clinical studies with control groups (including randomised controlled trials, non-randomised controlled trials, split-mouth trials and prospective 2-arm observational studies), respectively. The apparent wealth of laboratory evidence is sometimes used as basis for clinical inference and recommendations for daily dental practice. For example: In 2012, Ilie et al. recommended that selection of a suitable restorative material for clinical use in especially stress-bearing areas should been done with respect to *in-vitro* measured material properties, particularly in relation to its fracture toughness (K_IC_) [1]. Vichi et al. (2013) presumed that low *in-vitro* microleakage measurements for restorative materials translate into an adequate marginal seal when such materials are used for placing tooth restorations in the clinic [2], and based on laboratory shear bond strength (SBS) measurements, Ilie et al. (2014) suggested possible clinical advantages of using flowable bulk-fill resin composites for restoring deep, narrow cavities, with difficult access angles, and high-viscosity compounds for easier and faster restoration of larger tooth cavities [3].

In contrast, studies comparing the findings of both laboratory and clinical trials found only little correlation between the two. Papagiannoulis et al. (2002) established a lack of any correlation between *in-vivo* and *in-vitro* models in terms of artificial caries experiments and suggested that these may have only negligible clinical relevance in predicting the *in-vivo* effect [4]. Purk et al. (2004) established that the bonding of resin-based composite to teeth under *in-vivo* conditions yielded much weaker microtensile bond strengths than did bonding under *in-vitro* conditions and that bonding to dentin at the gingival wall under *in-vivo* conditions is weaker than that reported *in-vitro* studies [5]. In his review of dental literature, Heintze (2007) established that the quantitative marginal analysis of Class V fillings in the laboratory was unable to predict the performance of the same materials *in-vivo* [6] and Heintze and Cavalleri (2010) found that retention loss of Class V tooth restorations in nonretentive cavities could not be simulated in the laboratory, even after prolonged water storage and mechanical loading and thus could not reflect the clinical findings [7]. In contrast, van Meerbeek et al. (2010) found some indications for correlation of laboratory bond strength with clinical retention rates of Class-V restorations [8]. However, the predictive strength of the laboratory findings was only expressed as linear correlation and not in line with full Prentice requirements [9], and appeared to be weak and of borderline significance (r = 0.5811, p = 0.0475), only. In addition, Heintze and Zimmerli (2011) stated that *in-vitro* dye penetration measurements have no clinical relevance for the clinical performance of restorative materials, that marginal gap analysis in the laboratory is clinically relevant only to a limited extent and that bond strength tests are useful as screening tests, only [10].

Traditionally, glass ionomer cements are considered as unsuitable for clinical use as a permanent filling material in the posterior dentition due to *in-vitro* measured poor mechanical properties [1,11]. Specifically, *in-vitro* measured low material strength and wear resistance have been stated as reasons why glass-ionomers cannot rival amalgam as truly universal posterior restorative material [12].

However, based on the demonstrated general lack of any observed correlations between laboratory and clinical evidence, particularly related to tooth restorations [6,7,10] the *in-vitro* measured poor mechanical properties of glass ionomers, including high-viscosity glass-ionomers (HVGICs), may not translate into poor clinical results.

Against this general background, the aim of this meta-epidemiological study was to test two null-hypotheses:

H01: The results from laboratory trials concerning HVGICs versus amalgam indicate similar effect direction as results from controlled clinical trials concerning HVGICs versus amalgam restorations placed in permanent posterior teeth.
H02: The results from laboratory trials concerning HVGICs versus amalgam indicate similar effect magnitude as results from controlled clinical trials concerning HVGICs versus amalgam restorations placed in permanent posterior teeth.

## Methods

The protocol of this study has been published prior to its start in an open access journal [13] and is freely available online (http://www.jmid.org). Although the methodology of this meta-epidemiological study shared many aspects of a systematic review, its objective was methodological in nature. Therefore, the study protocol was not eligible for registration with the International Prospective Register of Systematic Reviews (PROSPERO) [14].

### Systematic literature search

The following databases: CENTRAL accessed via Cochrane Library; MEDLINE accessed via PubMed; Biomed Central; Database of Open Access Journals (DOAJ); IndMed; OpenSIGLE and Google Scholar were searched by both authors, independently using the search strategies for both laboratory and clinical trials, presented in Table 1. In addition to the search of databases, reference lists of accepted trial reports and systematic reviews, as well as narrative reviews, if found of importance to the topic, were checked for further suitable trials.

**Table 1:** Search strategy and results: electronic database search.

**[1] Laboratory trials**	
Electronic database	Number of Citations found
BMC search strategy: 14 September 2014 Online: http://www.biomedcentral.com/search/	
[1] Fuji IX AND amalgam AND in vitro	0
[2] Ketac Molar AND amalgam AND in vitro	0
[3] glass ionomer AND amalgam AND in vitro	6
[4] glass ionomer AND amalgam AND fracture strength	4
Articles included (Duplications removed)	0
CENTRAL (Trials) search strategy: 14 September 2014 Online: http://www.thecochranelibrary.com/view/0/index.html	
[1] Fuji IX AND amalgam AND in vitro	0
[2] Ketac Molar AND amalgam AND in vitro	1
[3] glass ionomer AND amalgam AND in vitro	11
[4] glass ionomer AND amalgam AND fracture strength	3
Articles included (Duplications removed)	0
DOAJ search strategy: 14 September 2014 Online: http://www.doaj.org	
[1] Fuji IX AND amalgam AND in vitro	0
[2] Ketac Molar AND amalgam AND in vitro	0
[3] glass ionomer AND amalgam AND in vitro	13
[4] glass ionomer AND amalgam AND fracture strength	3
Articles included (Duplications removed)	0
GoogleScholar search strategy: 14 September 2014 Online: http://scholar.google.co.za/	
[1] Fuji IX AND amalgam AND in vitro	553
[2] Ketac Molar AND amalgam AND in vitro	906
[3] glass ionomer AND amalgam AND in vitro	6270
[4] glass ionomer AND amalgam AND fracture strength	4290
Articles included (Duplications removed)	0
IndMed search strategy: 14 September 2014 Online: http://indmed.nic.in/	
[1] Fuji IX AND amalgam AND in vitro	0
[2] Ketac Molar AND amalgam AND in vitro	0
[3] glass ionomer AND amalgam AND in vitro	0
[4] glass ionomer AND amalgam AND fracture strength	0
Articles included (Duplications removed)	0
OpenSIGLE search strategy: 14 September 2014 Online: http://opensigle.inist.fr/	
[1] Fuji IX AND amalgam AND in vitro	0
[2] Ketac Molar AND amalgam AND in vitro	0
[3] glass ionomer AND amalgam AND in vitro	1
[4] glass ionomer AND amalgam AND fracture strength	0
Articles included (Duplications removed)	0
PubMed search strategy: 12 September 2014 Online: http://www.pubmed.org	
[1] Fuji IX AND amalgam AND in vitro	5
[2] Ketac Molar AND amalgam AND in vitro	1
[3] glass ionomer AND amalgam AND in vitro	161
[4] glass ionomer AND amalgam AND fracture strength	29
Articles included (Duplications removed)	15
Hand-search (Internet):	4
Reference check of included trial reports:	0
Total articles/documents included:	19
**[2] Clinical trials**	
BMC search strategy: 14 September 2014 Online: http://www.biomedcentral.com/search/	
[1] Fuji IX AND amalgam	1
[2] Ketac Molar AND amalgam	2
[3] glass ionomer AND amalgam	18
Articles included (Duplications removed)	0
CENTRAL (Trials) search strategy: 14 September 2014 Online: http://www.thecochranelibrary.com/view/0/index.html	
[1] Fuji IX AND amalgam	3
[2] Ketac Molar AND amalgam	8
[3] glass ionomer AND amalgam	102
Articles included (Duplications removed)	1
DOAJ search strategy: 14 September 2014 nline: http://www.doaj.org	
[1] Fuji IX AND amalgam	0
[2] Ketac Molar AND amalgam	1
[3] glass ionomer AND amalgam	30
Articles included (Duplications removed)	0
GoogleScholar search strategy: 14 September 2014 Online: http://scholar.google.co.za/	
[1] Fuji IX AND amalgam	1090
[2] Ketac Molar AND amalgam	1220
[3] glass ionomer AND amalgam	9910
Articles included (Duplications removed)	0
IndMed search strategy: 14 September 2014 Online: http://indmed.nic.in/	
[1] Fuji IX AND amalgam	0
[2] Ketac Molar AND amalgam	0
[3] glass ionomer AND amalgam	0
Articles included (Duplications removed)	0
OpenSIGLE search strategy: 14 September 2014 Online: http://opensigle.inist.fr/	
[1] Fuji IX AND amalgam	0
[2] Ketac Molar AND amalgam	0
[3] glass ionomer AND amalgam	2
Articles included (Duplications removed)	0
PubMed search strategy: 14 September 2014 Online: http://www.pubmed.org	
[1] Fuji IX AND amalgam	20
[2] Ketac Molar AND amalgam	17
[3] glass ionomer AND amalgam	842
Articles included (Duplications removed)	10
Hand-search (Internet):	2
Reference check of included trial reports:	0
Total articles/documents included:	13

The identified citations were eligible for possible inclusion if they followed the inclusion criteria:

1. Articles published in English;
2. Full reports of prospective controlled clinical (including randomised control trials and non- randomised control trials) and laboratory trials (including: *in-vitro*; *in-vivo* on animal tissues);
3. Head-to-head comparison of high-viscosity glass-ionomers (HVGIC) versus amalgam;
4. Longest follow-up period reported per trial;
5. Relevance to tooth restorations in posterior teeth of the permanent dentition;
6. Computable data reported:
  - Continuous data per intervention group: N = Number of evaluated units; x = Mean value of measured outcome; SD = Standard deviation or SE = Standard error.
  - Dichotomous data per intervention group: N = Number of evaluated units; n = Number of failed interventions.

Clinical trial participants included all patients of any age, gender or place of origin with restorable cavities in permanent posterior teeth.

Any provisionally included articles were further excluded, if: No computable dichotomous or continuous data was reported; test and control groups were not evaluated the same way; low-viscosity chemically cured, metal-reinforced, resin-modified or light-cured glass-ionomers were used as test intervention; reports and/or results of earlier follow-up periods than reported elsewhere; clinical trials investigating tunnel or sandwich restorations; clinical trials investigating restorations placed in primary and/or anterior teeth; dichotomous datasets with zero number of failed interventions (n = 0) in both test and control groups.

Both authors scanned titles and abstracts of identified citations from data sources in duplication. For articles with suitable titles but lacked listed abstracts the full reports were retrieved. Both authors judged separately all included articles; for possible exclusion, with reason, or acceptance, in line with the exclusion criteria. Any disagreements were resolved through discussion and consensus.

### Data extraction and Statistical Analysis

The outcome measure was the number of teeth with reported restoration failures (n) from the total number of evaluated teeth (N) for dichotomous data and the statistical mean (X) of outcomes with standard deviation (SD) or standard error (SE) from the total number of evaluated units (N) for continuous data (in cases were SE were reported instead of SD, the SE were converted into SD).

Data was extracted by both authors from accepted trials independently. The authors were not blinded to article authors, institutions, journal name and trial results. Disagreements between authors concerning data extracted were solved through discussion and consensus. All extracted data were entered in specifically designed data sheets in MS Excel. All completed data sheets were made available as S1–S3 Files. The following data was extracted: Article first author; year of publication and full article reference; (per test- and control group) product name of material used; number of subjects/units at beginning of trial (BSL); number of evaluated units at end of follow-up period (N); number of failures (n) for dichotomous data; statistical mean (X) of outcomes with standard deviation (SD) or standard error (SE) for continuous data; length of trial (follow-up period); verbatim conclusions and recommendations for clinical practice. A dichotomous dataset was defined as any extracted set of n / N for test- and control group. For each dichotomous dataset the Odds ratio (OR) with 95% Confidence intervals (CI) and p-values was computed. A continuous dataset was defined as any extracted set of N, X, SD or SE for test- and control group. For each continuous dataset the Standardised Mean Difference (SMD) [15] with 95% Confidence intervals (CI) and p-values was computed.

Statistical significance was set at alpha 5%. The statistical software programme RevMan 4.2 was used for computation of all point estimates.

### Null-hypothesis testing

In order to test the null-hypotheses (H01) that the results from laboratory trials and controlled clinical trials indicate similar effect directions, fixed-effects meta-analysis was conducted for clinical and laboratory data, separately, using RevMan 4.2 statistical software. A pooled Odds ratio (OR) and a pooled Standardised Mean Difference (SMD) with 95% CI and p—values for dichotomous and continuous data, respectively, were computed. Statistical significance for the pooled data was set at alpha 5%.

Three types of effect direction were considered:

1. Statistically significant differences (p<0.05) between HVGIC and amalgam (in favor of amalgam);
2. Statistically significant differences (p<0.05) between HVGIC and amalgam (in favor of HVGIC);
3. Lack of statistically significant differences (p>0.05) between HVGIC and amalgam.

Rejection of the null-hypothesis was based on the observation that the pooled effect estimates of both, clinical and laboratory trials have different effect direction (i)–(iii). Because the objective of this study was to investigate whether results from, both, clinical and laboratory trials generally point in the same effect direction and not to establish the actual clinical meaning of the pooled effect estimates, aspects of in-between-dataset heterogeneity was not considered during analysis.

In order to test the null-hypotheses (H02) that the results from laboratory trials and controlled clinical trials have similar effect magnitude the following analysis steps were undertaken:

1. Odds ratios (OR) with 95% Confidence intervals (CI) from clinical dichotomous data were converted into SMD with 95% CI using the tested method by Hasselblad and Hedges [15,16]:
  - Natural log (ln) transformation of OR values and Upper/Lower confidence interval limits;
  - Division of ln-values by 1.81
2. Statistical comparison of SMD point estimates from clinical and laboratory trials;
3. Statistical comparison of SMD ‘conservative’ point estimates, defined as Upper or Lower 95% Confidence levels closest to zero value, from clinical and laboratory trials.

The data from both, clinical and laboratory trials were considered to be independent from each other and the variances of both data types expected to be unequal. In addition, past systematic reviews of clinical trials in dentistry have shown only a limited number of head-to-head comparisons for posterior HVGIC and amalgam restorations in the permanent dentition [17] and thus a limited number of datasets (< 30) was expected to be available for analysis. Therefore, the Mann-Whitney U test was chosen as appropriate tool for statistical comparison. The statistical software Biostat 2009 was used for computation. Statistical significance was set at alpha 5%. Rejection of the null-hypothesis was based on the observation that the difference between the SMD point estimates from clinical and laboratory trials, was statistically significant (p < 0.05) for both statistical comparisons.

### Assessment of publication bias risk

It was planned to compute the I^2^ point-estimate with 95% CI of all extracted datasets for clinical and laboratory data, separately. Thresholds for I^2^ point estimates (in %) and its upper confidence values were used in order to interpret the I^2^ test results: 0–40% = might not be important; 30–60% = may represent moderate heterogeneity; 50–90% = may represent substantial heterogeneity; 75–100% = considerable heterogeneity [18]. High statistical in-between-datasets heterogeneity as per thresholds was taken under consideration when assessing publication bias risk by graphical and statistical methods.

It was planned to generate a funnel plot for clinical and laboratory data, separately, using a fixed-effects model with the natural logarithm (ln) of the Risk ratio (RR) as effect size estimate from all extracted dichotomous datasets and the Mean Difference (MD) for continuous datasets and examined for potential scatter asymmetry. The graphical findings were to be statistically verified using Egger’s regression [19]. Statistical significance was set at alpha 5%. The I^2^ point-estimate with 95% CI, funnel plot generation and Egger’s regression analysis was computed using MIX 1.7 statistical software [20]. Both, funnel plot and Egger’s regression would not be conducted if the number of extracted datasets were < 10 per data type.

### Directed acyclic graph (DAG)

The analysis results were further investigated within the context of potential influencing or confounding factors by use of a Directed acyclic graph (DAG). DAGs have been developed to graphically evaluate causal effects and to identify multiple confounders (or influences) within a causal system [21,22]. DAGs display a web of causation and consist of variables represented by alphabetic letters (A,B,..) and arrow lines that represent direct causal links between these variables.

## Results

### Systematic literature search

**Fig 1:**
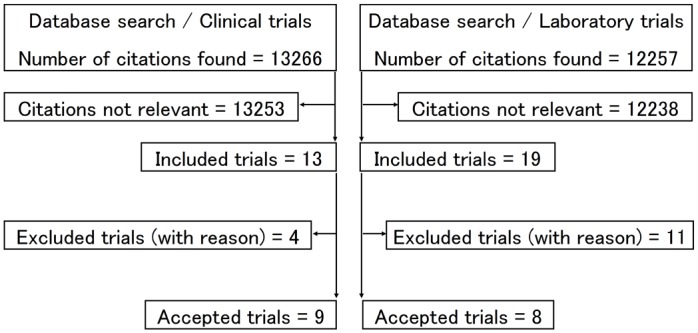
Flow diagram of trial selection.

Information on the number of articles identified are provided in Fig 1. The search of the electronic databases for clinical trials generated 13266 citations and the literature search of the electronic databases for laboratory trials generated 12257 citations. Of these, 11 clinical trial reports and 15 laboratory research reports were provisionally included. In addition, hand searching of the literature generated reports of two clinical and four laboratory trials (Table 1).

From the total provisionally included 13 clinical and 19 laboratory reports, four and 11 reports were excluded, respectively. The reasons for excluding the clinical trial reports were: Shorter reported period of same trial (n = 3) [23–25]; no computable dichotomous or continuous data reported (n = 1) [26]. The reasons for excluding the laboratory trial reports were: Non-English publication (n = 1) [27]; no HVGIC included in study (RMGIC used instead) or no clear indication that HVGIC was included in study (n = 7) [28–34]; no amalgam included in study (n = 1) [35]; no computable dichotomous or continuous data reported (n = 2) [36–37]. Nine clinical [38–46] and eight laboratory [47–54] trial reports were accepted for data extraction (S2 File).

### Data extraction and analysis

Of the accepted eight laboratory trials reports, five included no conclusions or recommendations for clinical practice based on its results [47,49–52] and one trial report cautioned the reader of any clinical extrapolation of its presented *in-vitro* results [53]. However, two of the eight reports included clear recommendations for daily clinical practice [48,54].

Of the accepted eight laboratory trials, 13 datasets (four dichotomous and nine continuous) could be extracted. Of the nine continuous laboratory datasets, six datasets indicated material efficacy for high measurement values [47,49,51,52,54] and three datasets indicated material efficacy for low measurement values [48,50,53]. Of the accepted clinical trials 21 dichotomous datasets could be extracted. Details of the extracted raw data are presented in S2 File.

### Null-hypothesis testing

In order to test the null-hypotheses (H01) that the results from laboratory trials and controlled clinical trials indicate similar effect directions, fixed-effects meta-analysis was conducted for clinical and laboratory data, separately from the extracted raw data presented in S2 File. Some *in-vitro* types of outcomes indicated material efficacy for measured low values and some *in-vitro* types of outcomes indicated material efficacy for measured high values. In addition, some of the laboratory data was not of continuous but of dichotomous nature. Due such heterogeneity of data types, the established laboratory dataset results could not be combined and were pooled separately, per data type.

**Fig 2:**
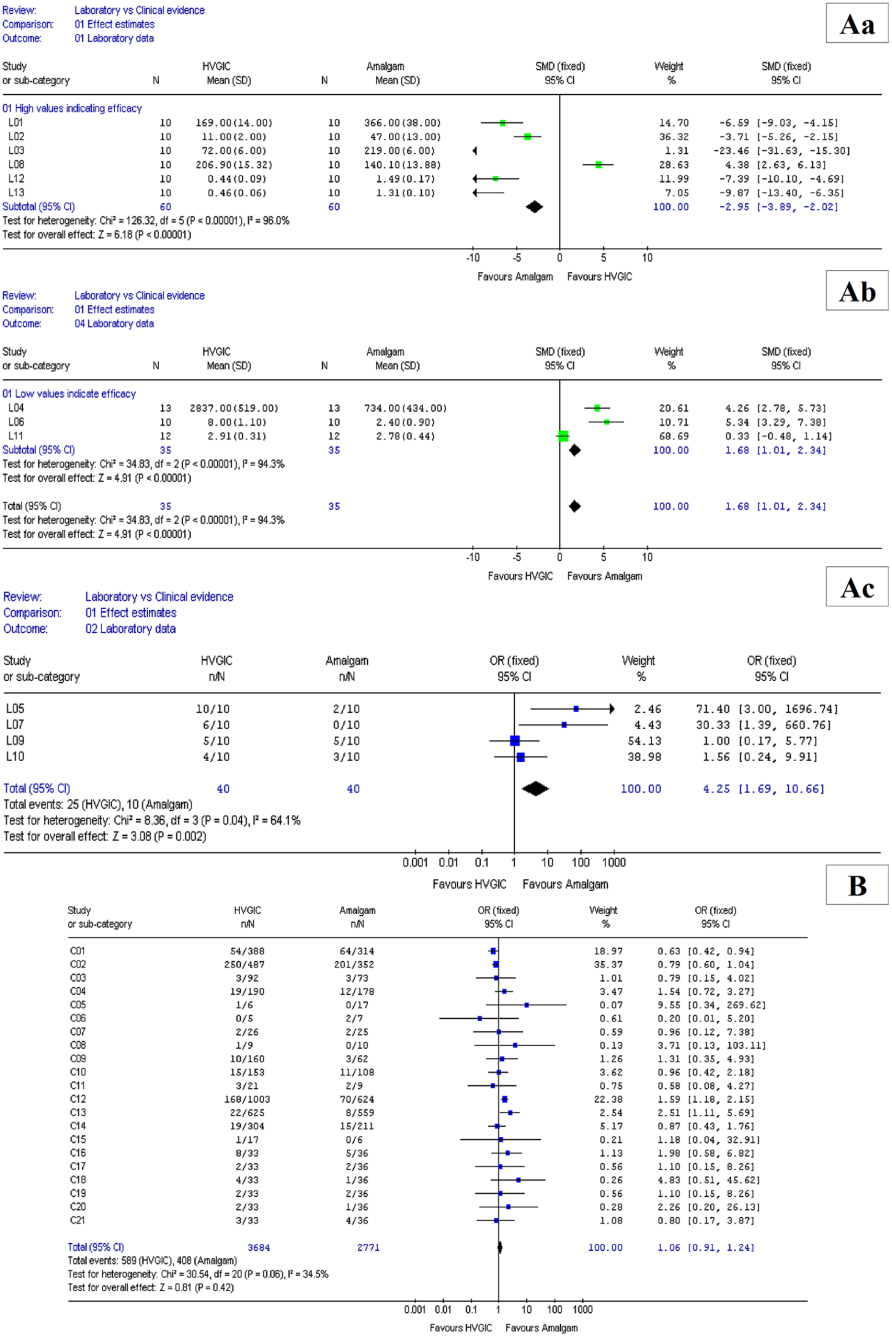
Meta-analysis results. Aa = Laboratory results, continuous data / measured lower values indicate material efficacy; Ab = Laboratory results, continuous data / measured higher values indicate material efficacy; Ac = Laboratory results, dichotomous data; B = Clinical results, dichotomous data.

All meta-analysis results are presented in four separate forest plots (Fig 2). The pooled results of the laboratory datasets for continuous and dichotomous data, SMD –2.95, 95%CI: -3.89, -2.02 (p < 0.00001); SMD 1.68, 95%CI: 1.01–2.34 (p < 0.00001) and OR 4.25, 95%CI: 1.69–10.66 (p = 0.02), respectively, were all highly statistically significant in favor of amalgam above HVGIC. In contrast, no statistically significant differences, between HVGICs and amalgam, were identified for clinical data (OR 1.06, 95%CI: 0.91–1.24; p = 0.42). These findings suggest that results from laboratory trials and controlled clinical trials do not indicate similar effect directions and thus the null-hypothesis (H01) was rejected.

In order to test the null-hypotheses (H02) that the results from laboratory trials and controlled clinical trials have similar effect magnitude all Odds ratios (OR) with 95% Confidence intervals (CI) from clinical dichotomous laboratory and clinical data were converted into SMD (95% CI). SMD point estimates from datasets where *in-vitro* types of outcomes indicated material efficacy for measured high values were converted into their opposite (+/-) values.

All converted values are presented in S3 File. The median SMD point estimates with 25% and 75% percentile range were –3.04 (-5.34; -0.24) and –0.08 (-0.38; 0.12) for laboratory and clinical data, respectively. The median SMD ‘conservative’ point estimates were –1.56 (-3.29; 0.48) and 0.08 (-0.42; 0.89) for laboratory and clinical data, respectively. The results of the comparison using Mann-Whitney U test indicted highly statistically significant differences between clinical and laboratory trial results for SMD point estimates (n_lab_ = 13; n_clin_ = 21; U = 46.5; p = 0.0014) and SMD ‘conservative’ point estimates (n_lab_ = 13; n_clin_ = 21; U = 64; p = 0.01). These findings suggest that results from laboratory trials and controlled clinical trials do not indicate similar effect magnitude and thus the null-hypothesis (H02) was rejected.

### Publication bias risk

Publication bias risk could only be assessed for clinical (n = 21) and not for laboratory data. Due to the latter’s large differences in data types, an insufficient number of datasets per data subgroup was available (n < 10), only.

**Fig 3:**
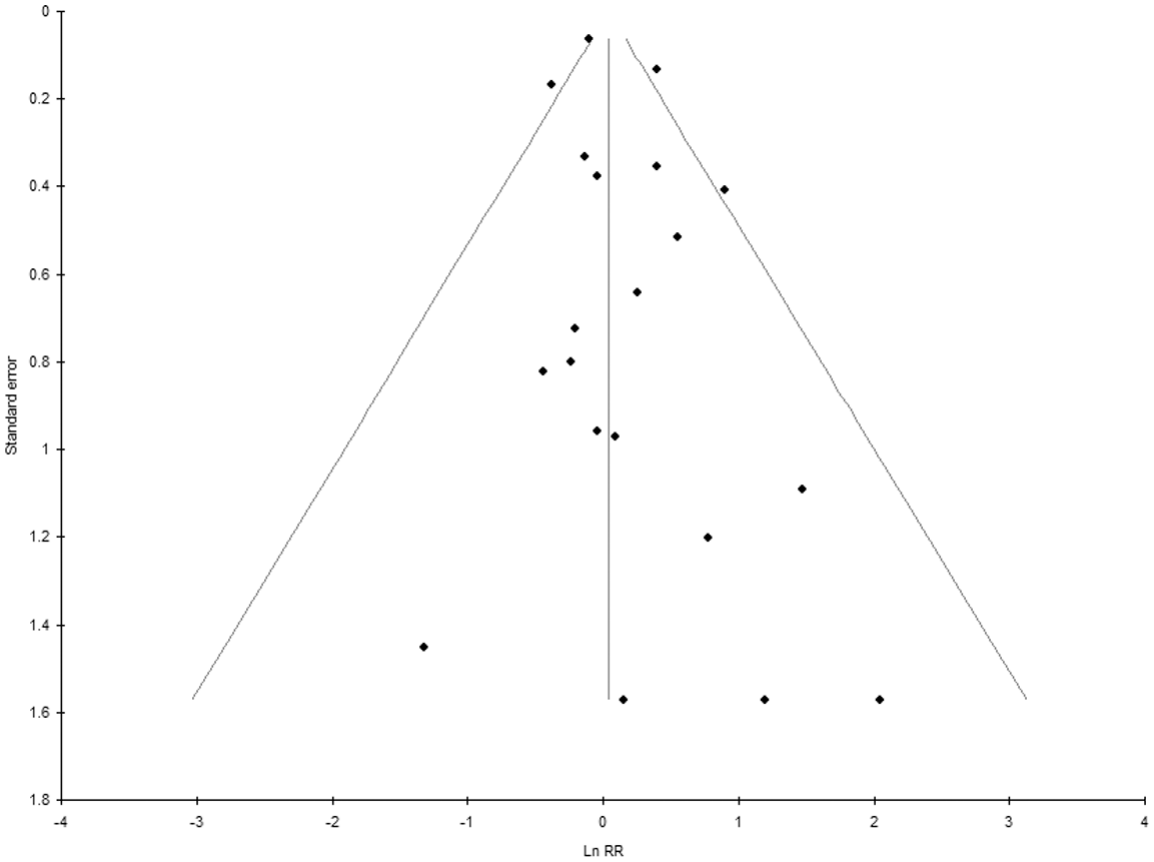
Funnel plot of clinical dataset results (test for publication bias). ln RR = natural logarithm Relative risk.

Statistical inter-dataset heterogeneity for clinical datasets was I^2^ = 34.5% (95% CI: 0–61.4%) and may represent low to moderate heterogeneity, only. The funnel plot (Fig 3) showed an even distribution, thus indicating low risk of publication bias. Egger’s linear regression method for the same datasets showed an intercept of 0.49 (95% CI: -0.19–1.18); p = 0.15, confirming low publication bias risk for clinical trials.

### Directed acyclic graph (DAG)

**Fig 4:**
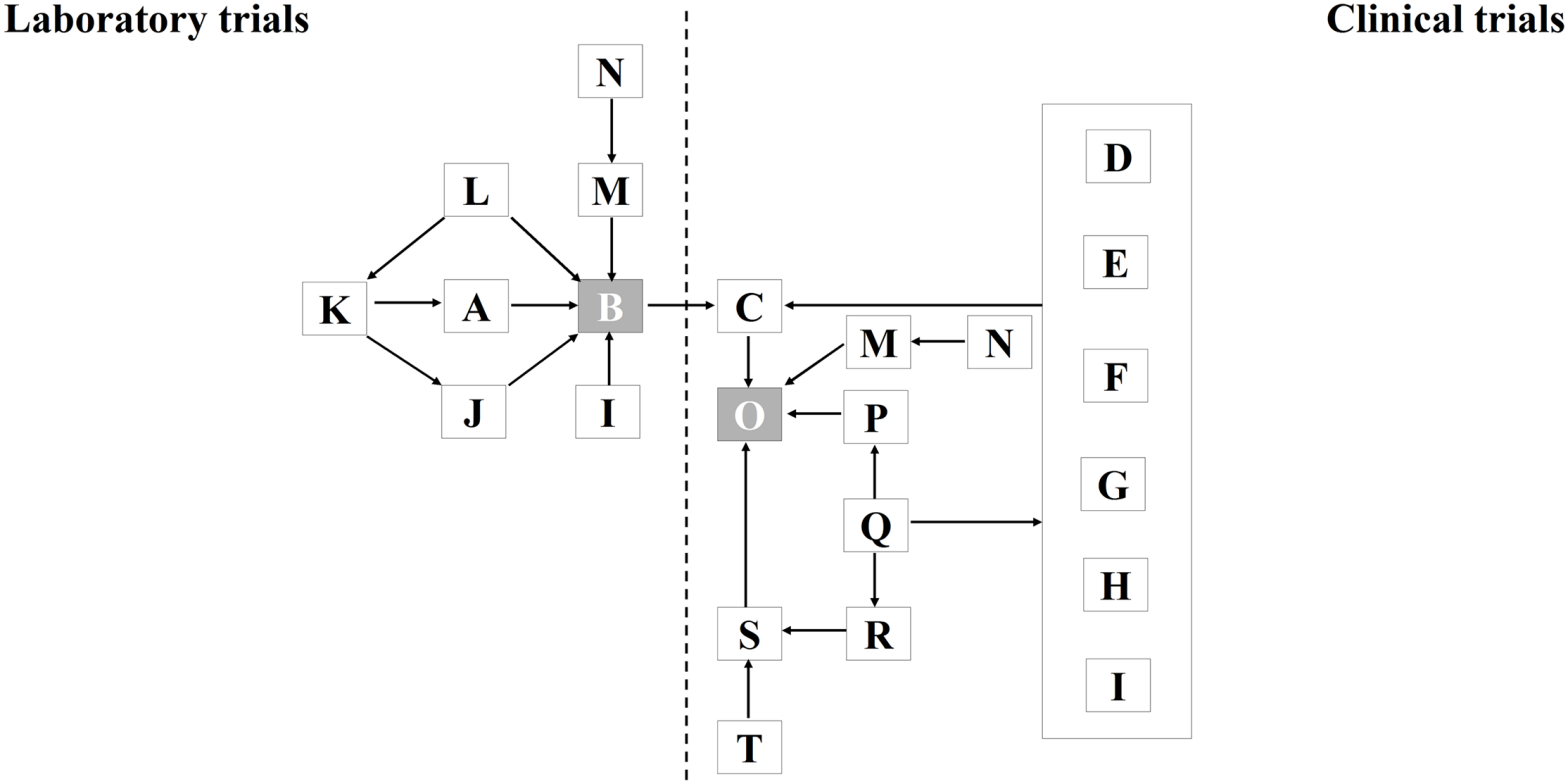
Directed acyclic graph (DAG). A = Material group characteristics; B = Measured laboratory outcome; C = True clinical outcome; D = Type of dentition; E = Type of tooth; F = Position of tooth in oral cavity; G = Type/Size of tooth cavity; H = Oral hygiene/habits of patient(s); I = Unknown influencing factors; J = Number of units evaluated (laboratory); K = Specimen selection process; L = Type of measurement; M = Quality /Characteristics of measurement procedure/evaluation process; N = Foreknowledge; O = Measured clinical outcome; P = Group characteristics; Q = Group selection process (randomisation); R = Number of units at baseline; S = Number of units evaluated (clinical); T = Number of units lost-to-follow-up.

The influence of factors on the compared variables, measured laboratory and clinical outcomes, were investigated using a DAG (Fig 4). The constructed DAG indicates that the measured laboratory outcome [B] may be determined by material group characteristics [A] but at the same time be influenced by unknown influencing factors [I], the number of evaluated units (sample size) [J], the type of measurement applied [L] and how well the measurement procedure was performed [M]. The latter may further be influenced by foreknowledge about the type of measured material, due to lack of evaluator blinding [N]. Material group characteristics [A] (e.g. one material being truly inferior/superior in properties to the other) may genuinely be the reason for the measured laboratory outcome [B] but may also be confounded by inadequate specimen selection process [K] (e.g. biased selection of artificially inferior/superior samples of a particular material).

Similarly, the measured clinical effect [O] may be genuine, due to the true clinical outcome [C], i.e. one material being truly inferior/superior to the other, but also due to biased group characteristic [P], the number of evaluated units [S] and a range of clinical conditions: Type of dentition [D], type of tooth [E], Position of tooth in the oral cavity [F], the type and/or size of the tooth cavity [G], any specific oral hygiene and oral habits of the patient [H] and other unknown influencing factors [I]. In addition, the number of evaluated units (sample size) [S] may be influenced by the number of units included at baseline [R] and the number of units lost-to-follow-up [T] at the end of the trial. The quality of the group selection process [Q], which may be particularly influenced by the adequacy of the randomisation procedure, may in turn determine the number of units included at baseline [R], group characteristic [P] and clinical conditions [D]–[I]. In addition, foreknowledge [N] about the type of measured material, due to lack of (operator, evaluator, patient) blinding, may influence the evaluation process [M].

The measured clinical effect [O] may correlate with the measured laboratory outcome [B] and at the same time correctly reflect the true clinical outcome [C]. However, the measured clinical effect [O] may also correctly reflect the true clinical outcome [C] but lack significant correlation with the measured laboratory outcomes [B] because too many other influencing factors were not included when establishing the latter.

## Discussion

### Limitations of study method

The aim of this meta-epidemiological study was to test the two null-hypotheses that the results from laboratory trials concerning HVGICs versus amalgam indicate similar effect direction (H01) and magnitude (H02) as results from controlled clinical trials concerning HVGICs versus amalgam restorations placed in permanent posterior teeth.

For this purpose, data was drawn from laboratory and clinical studies published in English, only. The reason for this language restriction was the consideration that English trial reports are more influential than reports published in other languages: English-language journals have a higher mean log impact factor than non-English language journals [55] and publishing in English appears to favor a high impact factor level and therefore, a high international citation frequency [56]. In addition, it has been shown that the inclusion of non-English trials may have little effect on summary treatment effect estimates and thus may be assumed as confirmatory of English publications [57,58]. Within the context of this study, the consideration of English publications having potentially higher impact on readers is particularly important as it highlights the influence that clinical recommendations based on laboratory results may have.

No assessment of internal trial validity was included in this study. However, an assessment of the majority of included clinical trials has been presented elsewhere [17]. Instead, a detailed discussion of potential influencing or confounding factors by use of a Directed acyclic graph (DAG) was chosen. Specific internal validity assessment, by evaluation of selection-, detection/performance and attrition bias was not conducted for each separate trial, as this would not have had relevance to the investigated question whether or not laboratory and clinical trial results are similar. However, the question whether laboratory data can generally have clinical relevance (or not) to this particular topic is pivotal within the context of this study and was explored in detail by use of a DAG.

A further limitation of this study in regard to its precision may have been introduced through the conversion of dichotomous into continuous data. In this regard, the results of this study rely on the empirically established accuracy for the applied conversion method by Hasselblad and Hedges, with a range of the Ratio of Odds ratios (ROR, being the ratio of the Odds ratio from the original and the Odds ratio from the converted data) for various clinical outcome measures between 0.80 (95% CI: 0.45–1.43) and 1.02 (95% CI: 0.90–1.16) [15].

### Study results

The results of this meta-epidemiology study suggest that the results from laboratory trials concerning HVGICs versus amalgam do not indicate similar effect direction and magnitude as results from controlled clinical trials concerning HVGICs versus amalgam restorations placed in permanent posterior teeth. These findings are in line with investigations in other topics, suggesting little correlation between *in-vitro* and *in-vivo* results [4–7]. While it was not possible to investigate publication bias risk within the laboratory literature, the results within the clinical literature suggest that publication bias risk is low.

The reason for the discrepancy between laboratory and clinical outcomes need to be regarded as unknown but can be assumed as being multi-factorial in nature as indicated in Fig 4:

A possible explanation of the differences may consider errors in effect measurement under either laboratory or clinical conditions. Errors under laboratory conditions may include: Mistakes made during the measurement process [M], the use of wrong measurement types [L], the confounding of material characteristics [A] due to biased specimen selection [K] or indeed unknown factors [I]. In specific regard to the analyzed trial data, six of the 13 extracted datasets from laboratory trials represent data concerning microleakage (S2 File). It has been stated that the general consensus among researchers who correlated *in-vivo* and *in-vitro* testing that laboratory microleakage tests are (i) inconsistent; (ii) fail to correlate with clinical margin discoloration and thus (iii) are not reliable tests and (iv) no valid predictors of clinical outcome [10,59].

Errors under clinical conditions may include: biased selection of trial subject [Q] with traits [D]–[I] / [P] that favor one intervention above the other (Selection bias); high loss to follow up [T] of trial subjects [S] that may influence result validity (Attrition bias risk); low samples size at baseline [R] that render the statistical power of the trial too low in order to detect true effect-size difference between the compared interventions and foreknowledge [N] due to lack of blinding that may affect the evaluation process [M] (Detection- and performance bias risk). From the nine included clinical trials, seven trials [38,40–45] were assessed for internal validity in a systematic review [17]. The results indicated high risk for selection-, detection/performance- and attrition bias, while four appeared statistical underpowered due to too low sample size [40–42,45]. The other two trials appeared to be limited by high detection-/performance bias risk due to lack of blinding [39] and poor statistical power due to too low sample size [46]. While these shortcomings may suggest possible reasons for the difference between laboratory [B] and clinical outcomes [C], they need to be regarded with caution, as the extent of their impact on direction and magnitude of the trial results remains unclear.

Notwithstanding possible explanations of differences due to errors in effect measurement, the difference between laboratory and clinical outcomes may also be explained on basis that any measured laboratory outcome [B] may be genuinely unable to correlate with any measured clinical effect [O] because too many other influencing factors, present under clinical conditions, are missing under laboratory conditions that cannot be sufficiently reproduced.

In line with laboratory trial evidence, the risk of microleakage may indeed be higher for placed HVGIC restorations than that of tooth restorations placed with amalgam. However, systematic review evidence of clinical trials indicate that such higher risk appears not only to translate into no higher risk of caries on restoration margins but such risk may even be substantially less for glass-ionomer restorations when compared to that of amalgam [60]. In this context, the presence of fluoride has been discussed as reason for a reduced susceptibility to secondary caries due to its potential to increase enamel resistance to acid demineralization [59]. Glass-ionomers contain fluoride, have been shown *in-vivo* to release fluoride into the oral cavity on a consistent basis [61] and have been associated with the development of a caries-protective hypermineralisation zone in walls of tooth cavities restored with glass-ionomers [62].

Furthermore, the *in-vitro* established lower physical strength of HVGIC in comparison to that of amalgam might not translate into a clinically higher fracture rate, because:

1. Placed glass-ionomer restorations are generally smaller than amalgam fillings [42], adhere to the tooth structure on basis of ion exchange between carboxylate and phosphate ions and thus do not require the preparation of macroretention areas in tooth cavities, like the latter;
2. Glass-ionomers placed in tooth cavities may abrade out of contact due to its potentially lower wear resistance.

For both reasons, HVGIC restoration may not be exposed to the same extent of daily masticatoric forces in the oral cavity than amalgam restorations are.

Therefore, while *in-vitro* measured material properties such as compressive strength, fracture toughness or microleakage of HVGIC may indeed be inferior to that of silver amalgam, these may not be sufficiently strong enough to translate into clinically meaningful differences, due to other influencing factors that are not present during laboratory trials.

## Conclusions

This study showed that laboratory results concerning HVGIC versus amalgam for tooth restorations have no similar effect direction and magnitude than that of controlled clinical trials. The reasons remain unclear but may be due to multifactor influences and confounding, particularly due to the lack of clinical factors that are absent in laboratory trials. Hence, while laboratory trial results may provide valuable explanations to this topic for observed clinical phenomena and may serve during the hypothesis development process, they appear not be suitable as basis for clinical inference and clinical recommendations.

## Supporting Information

S1 File Simple PubMed search of the dental literature published between 2009–2014. (DOC)

S2 File Data extracted from trial reports. (XLS)

S3 File Converted data values. (XLS)

S1 PRISMA Checklist PRISMA 2009 Checklist. (DOC)

S1 PRISMA Flow Chart PRISMA 2009 Flow Diagram. (DOC)
