# Supplementary material for: Do Laboratory Results Concerning High-Viscosity Glass-Ionomers versus Amalgam for Tooth Restorations Indicate Similar Effect Direction and Magnitude than that of Controlled Clinical Trials? - A Meta-Epidemiological Study
Source: PLoS One. 2015 Jul 13;10(7):e0132246. doi: 10.1371/journal.pone.0132246 (PMC4500394; doi:10.1371/journal.pone.0132246)
Supplement: S1 File — (DOC) [file pone.0132246.s001.doc]

Do laboratory results concerning high-viscosity glass-ionomers versus amalgam for tooth restorations indicate similar effect direction and magnitude than that of controlled clinical trials? - A meta-epidemiological study

Steffen Mickenautsch, Veerasamy Yengopal

File S1: Simple PubMed of the dental literature published

between 2009 - 2014

|  | Glass-ionomer | Silver amalgam |
| --- | --- | --- |
| Database searched | PubMed | PubMed |
| MeSH search terms | “Glass Ionomer Cements” | “Dental Amalgam” |
| Date of search | 27 August 2014 | 27 August 2014 |
| Number of full citations | 792 | 385 |
| Number of citations of clinical studies | 72 | 32 |
| Number of citations of laboratory studies | 513 | 85 |
| Laboratory/Clinical study ratio | 7.12 | 2.66 |
| Citations of clinical studies | 1/1: Gomes GM, Gomes OM, Gomes JC, Loguercio AD, Calixto AL, Reis A. Evaluation of different restorative techniques for filling flared root canals: fracture resistance and bond strength after mechanical fatigue. J Adhes Dent. 2014 Jun;16(3):267-76. doi: 10.3290/j.jad.a31940. PubMed PMID: 24779026.  2/2: Cakmak F, Turk T, Karadeniz EI, Elekdag-Turk S, Darendeliler MA. Physical properties of root cementum: part 24. Root resorption of the first premolars after 4 weeks of occlusal trauma. Am J Orthod Dentofacial Orthop. 2014 May;145(5):617-25. doi: 10.1016/j.ajodo.2013.12.027. PubMed PMID: 24785926.  3/3: Ding YJ, Yao H, Wang GH, Song H. A randomized double-blind placebo-controlled study of the efficacy of Clinpro XT varnish and Gluma dentin desensitizer on dentin hypersensitivity. Am J Dent. 2014 Apr;27(2):79-83. PubMed PMID: 25000665.  4/4: Casagrande L, Dalpian DM, Ardenghi TM, Zanatta FB, Balbinot CE, García-Godoy F, De Araujo FB. Randomized clinical trial of adhesive restorations in primary molars. 18-month results. Am J Dent. 2013 Dec;26(6):351-5. PubMed PMID: 24640441.  5/5: Koyuturk AE, Ozmen B, Tokay U, Tuloglu N, Sari ME, Sonmez TT. Two-year follow-up of indirect posterior composite restorations of permanent teeth with excessive material loss in pediatric patients: a clinical study. J Adhes Dent. 2013 Dec;15(6):583-90. doi: 10.3290/j.jad.a30897. PubMed PMID: 24278962.  6/6: Santamaria MP, da Silva Feitosa D, Casati MZ, Nociti FH Jr, Sallum AW, Sallum EA. Randomized controlled clinical trial evaluating connective tissue graft plus resin-modified glass ionomer restoration for the treatment of gingival recession associated with non-carious cervical lesion: 2-year follow-up. J Periodontol. 2013 Sep;84(9):e1-8. doi: 10.1902/jop.2013.120447. Epub 2013 Jan 31. PubMed PMID: 23368948.  7/7: Bonifácio CC, Hesse D, Raggio DP, Bönecker M, van Loveren C, van Amerongen WE. The effect of GIC-brand on the survival rate of proximal-ART restorations. Int J Paediatr Dent. 2013 Jul;23(4):251-8. doi: 10.1111/j.1365-263X.2012.01259.x. Epub 2012 Aug 14. PubMed PMID: 22891625.  8/8: Campus G, Carta G, Cagetti MG, Bossù M, Sale S, Cocco F, Conti G, Nardone M, Sanna G, Strohmenger L, Lingström P; Italian Experimental Group on Oral Health. Fluoride concentration from dental sealants: a randomized clinical trial. J Dent Res. 2013 Jul;92(7 Suppl):23S-8S. doi: 10.1177/0022034513484329. Epub 2013 May 20. PubMed PMID: 23690357.  9/9: Hilton TJ, Ferracane JL, Mancl L; Northwest Practice-based Research Collaborative in Evidence-based Dentistry (NWP). Comparison of CaOH with MTA for direct pulp capping: a PBRN randomized clinical trial. J Dent Res. 2013 Jul;92(7 Suppl):16S-22S. doi: 10.1177/0022034513484336. Epub 2013 May 20. PubMed PMID: 23690353; PubMed Central PMCID: PMC3706175.  10/10: Blatz MB, Mante FK, Saleh N, Atlas AM, Mannan S, Ozer F. Postoperative tooth sensitivity with a new self-adhesive resin cement--a randomized clinical trial. Clin Oral Investig. 2013 Apr;17(3):793-8. doi: 10.1007/s00784-012-0775-4. Epub 2012 Jul 11. PubMed PMID: 22782256.  11/11: Guler C, Yilmaz Y. A two-year clinical evaluation of glass ionomer and ormocer based fissure sealants. J Clin Pediatr Dent. 2013 Spring;37(3):263-7. PubMed PMID: 23855170.  12/12: Havale R, Anegundi RT, Indushekar K, Sudha P. Clinical and radiographic evaluation of pulpotomies in primary molars with formocresol, glutaraldehyde and ferric sulphate. Oral Health Dent Manag. 2013 Mar;12(1):24-31. PubMed PMID: 23474578.  13/13: Ferreira JM, Pinheiro SL, Sampaio FC, Menezes VA. Use of glass ionomer cement containing antibiotics to seal off infected dentin: a randomized clinical trial. Braz Dent J. 2013;24(1):68-73. PubMed PMID: 23657417.  14/14: Corralo DJ, Maltz M. Clinical and ultrastructural effects of different liners/restorative materials on deep carious dentin: a randomized clinical trial. Caries Res. 2013;47(3):243-50. doi: 10.1159/000345648. Epub 2013 Jan 19. PubMed PMID: 23343804.  15/15: Maltz M, Jardim JJ, Mestrinho HD, Yamaguti PM, Podestá K, Moura MS, de Paula  LM. Partial removal of carious dentine: a multicenter randomized controlled trial and 18-month follow-up results. Caries Res. 2013;47(2):103-9. doi: 10.1159/000344013. Epub 2012 Nov 28. PubMed PMID: 23207420.  16/16: Santamaria MP, Casati MZ, Nociti FH Jr, Sallum AW, Sallum EA, Aukhil I, Wallet SM, Shaddox LM. Connective tissue graft plus resin-modified glass ionomer restoration for the treatment of gingival recession associated with non-carious cervical lesions: microbiological and immunological results. Clin Oral Investig. 2013 Jan;17(1):67-77. doi: 10.1007/s00784-012-0690-8. Epub 2012 Feb 29. PubMed PMID: 22373777.  17/17: Chen Xx, Liu Xg. Clinical comparison of Fuji VII and a resin sealant in children at high and low risk of caries. Dent Mater J. 2013;32(3):512-8. PubMed PMID: 23719016.  18/18: Nosrat A, Seifi A, Asgary S. Pulpotomy in caries-exposed immature permanent molars using calcium-enriched mixture cement or mineral trioxide aggregate: a randomized clinical trial. Int J Paediatr Dent. 2013 Jan;23(1):56-63. doi: 10.1111/j.1365-263X.2012.01224.x. Epub 2012 Feb 6. PubMed PMID: 22309243.  19/19: Bezerra AC, Novaes RC, Faber J, Frencken JE, Leal SC. Ion concentration adjacent to glass-ionomer restorations in primary molars. Dent Mater. 2012 Nov;28(11):e259-63. doi: 10.1016/j.dental.2012.08.014. Epub 2012 Sep 20. PubMed PMID: 22999372.  20/20: Zhi QH, Lo EC, Lin HC. Randomized clinical trial on effectiveness of silver diamine fluoride and glass ionomer in arresting dentine caries in preschool children. J Dent. 2012 Nov;40(11):962-7. doi: 10.1016/j.jdent.2012.08.002. Epub 2012 Aug 11. PubMed PMID: 22892463.  21/21: Perdigão J, Dutra-Corrêa M, Saraceni SH, Ciaramicoli MT, Kiyan VH. Randomized clinical trial of two resin-modified glass ionomer materials: 1-year results. Oper Dent. 2012 Nov-Dec;37(6):591-601. doi: 10.2341/11-415-C. Epub 2012 Jul 7. PubMed PMID: 22770485.  22/22: Jongsma LA, Kleverlaan CJ, Feilzer AJ. Clinical success and survival of indirect resin composite crowns: results of a 3-year prospective study. Dent Mater. 2012 Sep;28(9):952-60. doi: 10.1016/j.dental.2012.04.007. Epub 2012 May  18. PubMed PMID: 22608959.  23/23: Huth KC, Hajek-Al-Khatar N, Wolf P, Ilie N, Hickel R, Paschos E. Long-term effectiveness of four pulpotomy techniques: 3-year randomised controlled trial. Clin Oral Investig. 2012 Aug;16(4):1243-50. doi: 10.1007/s00784-011-0602-3. Epub 2011 Aug 13. PubMed PMID: 21842145.  24/24: Andrade AK, Duarte RM, Silva FD, Batista AU, Lima KC, Pontual ML, Montes MA. Clinical evaluation of nanofill and nanohybrid composite in Class I restorations: a 12-month randomized trial. Gen Dent. 2012 Jul-Aug;60(4):e255-62. PubMed PMID: 22782061.  25/25: Roshan NM, Sakeenabi B. Anxiety in children during occlusal ART restorations in primary molars placed in school environment and hospital dental setup. J Clin Pediatr Dent. 2012 Summer;36(4):349-52. PubMed PMID: 23019830.  26/26: Phonghanyudh A, Phantumvanit P, Songpaisan Y, Petersen PE. Clinical evaluation of three caries removal approaches in primary teeth: a randomised controlled trial. Community Dent Health. 2012 Jun;29(2):173-8. PubMed PMID: 22779380.  27/27: Vigolo P, Mutinelli S. Evaluation of zirconium-oxide-based ceramic single-unit posterior fixed dental prostheses (FDPs) generated with two CAD/CAM systems compared to porcelain-fused-to-metal single-unit posterior FDPs: a 5-year clinical prospective study. J Prosthodont. 2012 Jun;21(4):265-9. doi: 10.1111/j.1532-849X.2011.00825.x. Epub 2012 Feb 19. PubMed PMID: 22339945.  28/28: Chen X, Du MQ, Fan MW, Mulder J, Huysmans MC, Frencken JE. Caries-preventive  effect of sealants produced with altered glass-ionomer materials, after 2 years. Dent Mater. 2012 May;28(5):554-60. doi: 10.1016/j.dental.2012.01.001. Epub 2012 Jan 31. PubMed PMID: 22300651.  29/29: Shetty RM, Bhat S, Mehta D, Srivatsa G, Shetty YB. Comparative analysis of postcementation hypersensitivity with glass ionomer cement and a resin cement: an in vivo study. J Contemp Dent Pract. 2012 May 1;13(3):327-31. PubMed PMID: 22918005.  30/30: Ulusu T, Odabaş ME, Tüzüner T, Baygin O, Sillelioğlu H, Deveci C, Gökdoğan FG, Altuntaş A. The success rates of a glass ionomer cement and a resin-based fissure sealant placed by fifth-year undergraduate dental students. Eur Arch Paediatr Dent. 2012 Apr;13(2):94-7. PubMed PMID: 22449810.  31/31: Corbacho de Melo MM, Cardoso MG, Faber J, Sobral A. Risk factors for periodontal changes in adult patients with banded second molars during orthodontic treatment. Angle Orthod. 2012 Mar;82(2):224-8. doi: 10.2319/030911-172.1. Epub 2011 Sep 7. PubMed PMID: 21899378.  32/32: Baracco B, Perdigão J, Cabrera E, Giráldez I, Ceballos L. Clinical evaluation of a low-shrinkage composite in posterior restorations: one-year results. Oper Dent. 2012 Mar-Apr;37(2):117-29. doi: 10.2341/11-179-C. Epub 2012 Feb 7. PubMed PMID: 22313275.  33/33: Dos Santos VE Jr, de Vasconcelos FM, Ribeiro AG, Rosenblatt A. Paradigm shift in the effective treatment of caries in schoolchildren at risk. Int Dent J. 2012 Feb;62(1):47-51. doi: 10.1111/j.1875-595X.2011.00088.x. PubMed PMID: 22251037.  34/34: Antonson SA, Antonson DE, Brener S, Crutchfield J, Larumbe J, Michaud C, Yazici AR, Hardigan PC, Alempour S, Evans D, Ocanto R. Twenty-four month clinical evaluation of fissure sealants on partially erupted permanent first molars: glass ionomer versus resin-based sealant. J Am Dent Assoc. 2012 Feb;143(2):115-22. Erratum in: J Am Dent Assoc. 2012 Apr;143(4):336. PubMed PMID: 22298552.  35/35: Dhar V, Chen H. Evaluation of resin based and glass ionomer based sealants placed with or without tooth preparation-a two year clinical trial. Pediatr Dent. 2012 Jan-Feb;34(1):46-50. PubMed PMID: 22353457.  36/36: De Menezes Abreu DM, Leal SC, Mulder J, Frencken JE. Dental anxiety in 6-7-year-old children treated in accordance with conventional restorative treatment, ART and ultra-conservative treatment protocols. Acta Odontol Scand. 2011 Nov;69(6):410-6. doi: 10.3109/00016357.2011.572561. Epub 2011 Apr 27. PubMed PMID: 21524172.  37/37: Dere H, Ozdogan F, Ozcan KM, Selcuk A, Ozcan I, Gokturk G. Comparison of glass ionomer cement and incus interposition in reconstruction of incus long process defects. Eur Arch Otorhinolaryngol. 2011 Nov;268(11):1565-8. doi: 10.1007/s00405-010-1454-1. Epub 2011 Feb 22. PubMed PMID: 21340562.  38/38: Maghrabi AA. Effect of dentin sealers on postoperative sensitivity of complete cast crowns cemented with glass ionomer cement. J Prosthodont. 2011 Jul;20(5):385-90. doi: 10.1111/j.1532-849X.2011.00724.x. Epub 2011 May 31. PubMed PMID: 21627710.  39/39: Ibiyemi O, Bankole OO, Oke GA. Survival rates of two atraumatic restorative treatment (ART) types in occlusal carious permanent teeth after two years. Afr J Med Med Sci. 2011 Jun;40(2):127-34. PubMed PMID: 22195380.  40/40: Nazir M, Walsh T, Mandall NA, Matthew S, Fox D. Banding versus bonding of  first permanent molars: a multi-centre randomized controlled trial. J Orthod. 2011 Jun;38(2):81-9. doi: 10.1179/14653121141308. PubMed PMID: 21677099.  41/41: Kemoli AM, Opinya GN, van Amerongen WE, Mwalili SM. Two-year survival rates of proximal atraumatic restorative treatment restorations in relation to glass ionomer cements and Postrestoration meals consumed. Pediatr Dent. 2011 May-Jun;33(3):246-51. PubMed PMID: 21703078.  42/42: Tagliaferro EP, Pardi V, Ambrosano GM, Meneghim Mde C, da Silva SR, Pereira AC. Occlusal caries prevention in high and low risk schoolchildren. A clinical trial. Am J Dent. 2011 Apr;24(2):109-14. PubMed PMID: 21698991.  43/43: De Moor RJ, Stassen IG, van 't Veldt Y, Torbeyns D, Hommez GM. Two-year clinical performance of glass ionomer and resin composite restorations in xerostomic head- and neck-irradiated cancer patients. Clin Oral Investig. 2011 Feb;15(1):31-8. doi: 10.1007/s00784-009-0355-4. Epub 2009 Dec 8. PubMed PMID: 19997859.  44/44: Liu H, Zhou Q, Qin M. Mineral trioxide aggregate versus calcium hydroxide for pulpotomy in primary molars. Chin J Dent Res. 2011;14(2):121-5. PubMed PMID: 22319753.  45/45: Rinke S, Schäfer S, Roediger M. Complication rate of molar crowns: a practice-based clinical evaluation. Int J Comput Dent. 2011;14(3):203-18. English, German. PubMed PMID: 22141230.  46/46: de Andrade AK, Duarte RM, Medeiros e Silva FD, Batista AU, Lima KC, Pontual ML, Montes MA. 30-Month randomised clinical trial to evaluate the clinical performance of a nanofill and a nanohybrid composite. J Dent. 2011 Jan;39(1):8-15. doi: 10.1016/j.jdent.2010.09.005. Epub 2010 Oct 1. PubMed PMID: 20888884.  47/47: Trairatvorakul C, Itsaraviriyakul S, Wiboonchan W. Effect of glass-ionomer cement on the progression of proximal caries. J Dent Res. 2011 Jan;90(1):99-103. doi: 10.1177/0022034510381265. Epub 2010 Sep 21. PubMed PMID: 20858776.  48/48: Deepa G, Shobha T. A clinical evaluation of two glass ionomer cements in primary molars using atraumatic restorative treatment technique in India: 1 year follow up. Int J Paediatr Dent. 2010 Nov;20(6):410-8. doi:  10.1111/j.1365-263X.2010.01067.x. PubMed PMID: 20642467.  49/49: Orhan AI, Oz FT, Orhan K. Pulp exposure occurrence and outcomes after 1- or 2-visit indirect pulp therapy vs complete caries removal in primary and permanent molars. Pediatr Dent. 2010 Jul-Aug;32(4):347-55. PubMed PMID: 20836956.  50/50: Bjørndal L, Reit C, Bruun G, Markvart M, Kjaeldgaard M, Näsman P, Thordrup M, Dige I, Nyvad B, Fransson H, Lager A, Ericson D, Petersson K, Olsson J, Santimano EM, Wennström A, Winkel P, Gluud C. Treatment of deep caries lesions in adults: randomized clinical trials comparing stepwise vs. direct complete excavation, and direct pulp capping vs. partial pulpotomy. Eur J Oral Sci. 2010 Jun;118(3):290-7. doi: 10.1111/j.1600-0722.2010.00731.x. PubMed PMID: 20572864.  51/51: Alves dos Santos MP, Luiz RR, Maia LC. Randomised trial of resin-based restorations in Class I and Class II beveled preparations in primary molars: 48-month results. J Dent. 2010 Jun;38(6):451-9. doi: 10.1016/j.jdent.2010.02.004. Epub 2010 Feb 25. PubMed PMID: 20188783.  52/52: Mendonça JS, Neto RG, Santiago SL, Lauris JR, Navarro MF, de Carvalho RM. Direct resin composite restorations versus indirect composite inlays: one-year results. J Contemp Dent Pract. 2010 May 1;11(3):025-32. PubMed PMID: 20461321.  53/53: van Dijken JW, Pallesen U. Fracture frequency and longevity of fractured resin composite, polyacid-modified resin composite, and resin-modified glass ionomer cement class IV restorations: an up to 14 years of follow-up. Clin Oral Investig. 2010 Apr;14(2):217-22. doi: 10.1007/s00784-009-0287-z. Epub 2009 Jun 6.  PubMed PMID: 19504133.  54/54: Târcă T, Bădescu A, Topoliceanu C, Lăcătuşu S. [Clinical and microbiological  study regarding surface antibacterial properties of bioactive dental materials]. Rev Med Chir Soc Med Nat Iasi. 2010 Apr-Jun;114(2):536-41. Romanian. PubMed PMID: 20700999.  55/55: Lutfi AN, Kannan TP, Fazliah MN, Jamaruddin MA, Saidi J. Proliferative activity of cells from remaining dental pulp in response to treatment with dental materials. Aust Dent J. 2010 Mar;55(1):79-85. doi: 10.1111/j.1834-7819.2009.01185.x. PubMed PMID: 20415916.  56/56: Sly EG, Kaplan AE, Missana L. Clinical evaluation of glass ionomer for pit and fissure sealing of fully erupted molars. Acta Odontol Latinoam. 2010;23(1):3-7. PubMed PMID: 20645635.  57/57: Santiago SL, Passos VF, Vieira AH, Navarro MF, Lauris JR, Franco EB. Two-year  clinical evaluation of resinous restorative systems in non-carious cervical lesions. Braz Dent J. 2010;21(3):229-34. PubMed PMID: 21203706.  58/58: Płuciennik-Stronias M, Sakowska D, Krzemiński Z, Piatowska D. [Influence of topical fluoridation of glass ionomer cements on inhibitory activity on cariogenic bacteria]. Med Dosw Mikrobiol. 2010;62(4):369-74. Polish. PubMed PMID: 21473102.  59/59: Ertugrul F, Cogulu D, Ozdemir Y, Ersin N. Comparison of conventional versus colored compomers for class II restorations in primary molars: a 12-month clinical study. Med Princ Pract. 2010;19(2):148-52. doi: 10.1159/000273077. Epub 2010 Feb 4. PubMed PMID: 20134179.  60/60: Shi L, Wang X, Zhao Q, Zhang Y, Zhang L, Ren Y, Chen Z. Evaluation of packable and conventional hybrid resin composites in Class I restorations: three-year results of a randomized, double-blind and controlled clinical trial. Oper Dent. 2010 Jan-Feb;35(1):11-9. doi: 10.2341/09-027CR. PubMed PMID: 20166406.  61/61: Baseggio W, Naufel FS, Davidoff DC, Nahsan FP, Flury S, Rodrigues JA. Caries-preventive efficacy and retention of a resin-modified glass ionomer cement and a resin-based fissure sealant: a 3-year split-mouth randomised clinical trial. Oral Health Prev Dent. 2010;8(3):261-8. PubMed PMID: 20848004.  62/62: Duque C, Negrini Tde C, Sacono NT, Spolidorio DM, de Souza Costa CA, Hebling  J. Clinical and microbiological performance of resin-modified glass-ionomer liners after incomplete dentine caries removal. Clin Oral Investig. 2009 Dec;13(4):465-71. doi: 10.1007/s00784-009-0304-2. Epub 2009 Jun 23. PubMed PMID: 19548010.  63/63: Kemoli AM, van Amerongen WE, Opinya G. Influence of the experience of operator and assistant on the survival rate of proximal ART restorations: two-year results. Eur Arch Paediatr Dent. 2009 Dec;10(4):227-32. PubMed PMID: 19995507.  64/64: Wolfart S, Harder S, Eschbach S, Lehmann F, Kern M. Four-year clinical results of fixed dental prostheses with zirconia substructures (Cercon): end abutments vs. cantilever design. Eur J Oral Sci. 2009 Dec;117(6):741-9. doi: 10.1111/j.1600-0722.2009.00693.x. PubMed PMID: 20121939.  65/65: Kemoli AM, van Amerongen WE. Influence of the cavity-size on the survival rate of proximal ART restorations in primary molars. Int J Paediatr Dent. 2009 Nov;19(6):423-30. doi: 10.1111/j.1365-263X.2009.01013.x. Epub 2009 Sep 1. PubMed  PMID: 19732191.  66/66: Malkhassian G, Manzur AJ, Legner M, Fillery ED, Manek S, Basrani BR, Friedman S. Antibacterial efficacy of MTAD final rinse and two percent chlorhexidine gel medication in teeth with apical periodontitis: a randomized double-blinded clinical trial. J Endod. 2009 Nov;35(11):1483-90. doi: 10.1016/j.joen.2009.08.003. Epub 2009 Sep 18. PubMed PMID: 19840635.  67/67: Oba AA, Dülgergil T, Sönmez IS, Doğan S. Comparison of caries prevention with glass ionomer and composite resin fissure sealants. J Formos Med Assoc. 2009 Nov;108(11):844-8. doi: 10.1016/S0929-6646(09)60415-0. PubMed PMID: 19933027.  68/68: Burrow MF, Banomyong D, Harnirattisai C, Messer HH. Effect of glass-ionomer cement lining on postoperative sensitivity in occlusal cavities restored with resin composite--a randomized clinical trial. Oper Dent. 2009 Nov-Dec;34(6):648-55. doi: 10.2341/08-098-C. PubMed PMID: 19953773.  69/69: Daou MH, Tavernier B, Meyer JM. Two-year clinical evaluation of three restorative materials in primary molars. J Clin Pediatr Dent. 2009 Fall;34(1):53-8. PubMed PMID: 19953810.  70/70: Santamaria MP, Ambrosano GM, Casati MZ, Nociti Júnior FH, Sallum AW, Sallum EA. Connective tissue graft plus resin-modified glass ionomer restoration for the treatment of gingival recession associated with non-carious cervical lesion: a randomized-controlled clinical trial. J Clin Periodontol. 2009 Sep;36(9):791-8.  doi: 10.1111/j.1600-051X.2009.01441.x. Epub 2009 Jul 7. PubMed PMID: 19594663.  71/71: Wilson TG Jr. The positive relationship between excess cement and peri-implant disease: a prospective clinical endoscopic study. J Periodontol. 2009 Sep;80(9):1388-92. doi: 10.1902/jop.2009.090115. PubMed PMID: 19722787.  72/72: Khairallah C, Hokayem A. [Long-term clinical evaluation of 2 dental materials used for the preparation of esthetic inlays]. Odontostomatol Trop. 2009 Sep;32(127):5-13. French. PubMed PMID: 20441124. | 1/1: Kisakol G. Dental amalgam implantation and thyroid autoimmunity. Bratisl Lek Listy. 2014;115(1):22-4. PubMed PMID: 24471898.  2/2: McCracken MS, Gordan VV, Litaker MS, Funkhouser E, Fellows JL, Shamp DG, Qvist V, Meral JS, Gilbert GH; National Dental Practice-Based Research Network Collaborative Group. A 24-month evaluation of amalgam and resin-based composite restorations: Findings from The National Dental Practice-Based Research Network. J Am Dent Assoc. 2013 Jun;144(6):583-93. PubMed PMID: 23729455; PubMed Central PMCID: PMC3694730.  3/3: Gilbert GH, Gordan VV, Funkhouser EM, Rindal DB, Fellows JL, Qvist V, Anderson G, Worley D; National Dental PBRN Collaborative Group. Caries treatment in a dental practice-based research network: movement toward stated evidence-based treatment. Community Dent Oral Epidemiol. 2013 Apr;41(2):143-53. doi: 10.1111/cdoe.12008. Epub 2012 Oct 5. PubMed PMID: 23036131; PubMed Central PMCID: PMC3811014.  4/4: Martin J, Fernandez E, Estay J, Gordan VV, Mjor IA, Moncada G. Minimal invasive treatment for defective restorations: five-year results using sealants. Oper Dent. 2013 Mar-Apr;38(2):125-33. doi: 10.2341/12-062C. Epub 2012 Jul 11. PubMed PMID: 22788726.  5/5: Maltz M, Jardim JJ, Mestrinho HD, Yamaguti PM, Podestá K, Moura MS, de Paula LM. Partial removal of carious dentine: a multicenter randomized controlled trial  and 18-month follow-up results. Caries Res. 2013;47(2):103-9. doi: 10.1159/000344013. Epub 2012 Nov 28. PubMed PMID: 23207420.  6/6: Bracher AK, Hofmann C, Bornstedt A, Hell E, Janke F, Ulrici J, Haller B, Geibel MA, Rasche V. Ultrashort echo time (UTE) MRI for the assessment of caries lesions. Dentomaxillofac Radiol. 2013;42(6):20120321. doi: 10.1259/dmfr.20120321. Epub 2013 Feb 18. PubMed PMID: 23420857; PubMed Central PMCID: PMC3667523.  7/7: Tüzüner T, Alacam A, Altunbas DA, Gokdogan FG, Gundogdu E. Clinical and radiographic outcomes of direct pulp capping therapy in primary molar teeth following haemostasis with various antiseptics: a randomised controlled trial. Eur J Paediatr Dent. 2012 Dec;13(4):289-92. PubMed PMID: 23270285.  8/8: Maserejian NN, Hauser R, Tavares M, Trachtenberg FL, Shrader P, McKinlay S. Dental composites and amalgam and physical development in children. J Dent Res. 2012 Nov;91(11):1019-25. doi: 10.1177/0022034512458691. Epub 2012 Sep 12. PubMed  PMID: 22972857; PubMed Central PMCID: PMC3525131.  9/10: Maserejian NN, Trachtenberg FL, Hauser R, McKinlay S, Shrader P, Bellinger DC. Dental composite restorations and neuropsychological development in children: treatment level analysis from a randomized clinical trial. Neurotoxicology. 2012 Oct;33(5):1291-7. doi: 10.1016/j.neuro.2012.08.001. Epub 2012 Aug 14. PubMed PMID: 22906860; PubMed Central PMCID: PMC3470777.  10/11: Naimi-Akbar A, Svedberg P, Alexanderson K, Ekstrand J, Sandborgh-Englund G. Reliance on social security benefits by Swedish patients with ill-health attributed to dental fillings: a register-based cohort study. BMC Public Health. 2012 Aug 30;12:713. doi: 10.1186/1471-2458-12-713. PubMed PMID: 22935213; PubMed Central PMCID: PMC3487969.  11/12: Yaman E, Görken F, Pinar Erdem A, Sepet E, Aytepe Z. Effects of folk medicinal plant extract Ankaferd Blood Stopper(®) in vital primary molar pulpotomy. Eur Arch Paediatr Dent. 2012 Aug;13(4):197-202. PubMed PMID: 22883359.  12/13: Maserejian NN, Trachtenberg FL, Hauser R, McKinlay S, Shrader P, Tavares M, Bellinger DC. Dental composite restorations and psychosocial function in children. Pediatrics. 2012 Aug;130(2):e328-38. doi: 10.1542/peds.2011-3374. Epub 2012 Jul 16. PubMed PMID: 22802599; PubMed Central PMCID: PMC3408688.  13/14: Neelakantan P, Rao CV, Indramohan J. Bacteriology of deep carious lesions underneath amalgam restorations with different pulp-capping materials--an in vivo analysis. J Appl Oral Sci. 2012 Mar-Apr;20(2):139-45. PubMed PMID: 22666827; PubMed Central PMCID: PMC3894753.  14/15: Overton JD, Sullivan DJ. Early failure of Class II resin composite versus Class II amalgam restorations placed by dental students. J Dent Educ. 2012 Mar;76(3):338-40. PubMed PMID: 22383602.  15/16: Geier DA, Carmody T, Kern JK, King PG, Geier MR. A dose-dependent relationship between mercury exposure from dental amalgams and urinary mercury levels: a further assessment of the Casa Pia Children's Dental Amalgam Trial. Hum Exp Toxicol. 2012 Jan;31(1):11-7. doi: 10.1177/0960327111417264. Epub 2011 Jul 29. PubMed PMID: 21803780.  16/17: Thygesen LC, Flachs EM, Hanehøj K, Kjuus H, Juel K. Hospital admissions for neurological and renal diseases among dentists and dental assistants occupationally exposed to mercury. Occup Environ Med. 2011 Dec;68(12):895-901. doi: 10.1136/oem.2010.064063. Epub 2011 Apr 20. PubMed PMID: 21508427.  17/18: De Menezes Abreu DM, Leal SC, Mulder J, Frencken JE. Dental anxiety in 6-7-year-old children treated in accordance with conventional restorative treatment, ART and ultra-conservative treatment protocols. Acta Odontol Scand. 2011 Nov;69(6):410-6. doi: 10.3109/00016357.2011.572561. Epub 2011 Apr 27. PubMed PMID: 21524172.  18/19: Sjursen TT, Lygre GB, Dalen K, Helland V, Laegreid T, Svahn J, Lundekvam BF, Björkman L. Changes in health complaints after removal of amalgam fillings. J Oral Rehabil. 2011 Nov;38(11):835-48. doi: 10.1111/j.1365-2842.2011.02223.x. Epub 2011 Apr 23. PubMed PMID: 21517933; PubMed Central PMCID: PMC3229679.  19/20: Samir AM, Aref WM. Impact of occupational exposure to elemental mercury on some antioxidative enzymes among dental staff. Toxicol Ind Health. 2011 Oct;27(9):779-86. doi: 10.1177/0748233710397420. Epub 2011 Mar 22. PubMed PMID: 21427135.  20/21: Malekafzali B, Shekarchi F, Asgary S. Treatment outcomes of pulpotomy in primary molars using two endodontic biomaterials. A 2-year randomised clinical trial. Eur J Paediatr Dent. 2011 Sep;12(3):189-93. PubMed PMID: 22077689.  21/22: Gordan VV, Riley JL 3rd, Blaser PK, Mondragon E, Garvan CW, Mjör IA. Alternative treatments to replacement of defective amalgam restorations: results of a seven-year clinical study. J Am Dent Assoc. 2011 Jul;142(7):842-9. PubMedPMID: 21719808.  22/23: Geier DA, Carmody T, Kern JK, King PG, Geier MR. A significant relationship between mercury exposure from dental amalgams and urinary porphyrins: a further assessment of the Casa Pia children's dental amalgam trial. Biometals. 2011 Apr;24(2):215-24. doi: 10.1007/s10534-010-9387-0. Epub 2010 Nov 5. PubMed PMID: 21053054.  23/24: Erdem AP, Guven Y, Balli B, Ilhan B, Sepet E, Ulukapi I, Aktoren O. Success rates of mineral trioxide aggregate, ferric sulfate, and formocresol pulpotomies: a 24-month study. Pediatr Dent. 2011 Mar-Apr;33(2):165-70. PubMed PMID: 21703067.  24/25: Fernández EM, Martin JA, Angel PA, Mjör IA, Gordan VV, Moncada GA. Survival rate of sealed, refurbished and repaired defective restorations: 4-year follow-up. Braz Dent J. 2011;22(2):134-9. PubMed PMID: 21537587.  25/26: Alptekin T, Ozer F, Unlu N, Cobanoglu N, Blatz MB. In vivo and in vitro evaluations of microleakage around Class I amalgam and composite restorations. Oper Dent. 2010 Nov-Dec;35(6):641-8. doi: 10.2341/10-065-L. PubMed PMID: 21180003.  26/27: Ahrari F, Nojoomian M, Moosavi H. Clinical evaluation of bonded amalgam restorations in endodontically treated premolar teeth: a one-year evaluation. J Contemp Dent Pract. 2010 Oct 14;11(5):009-16. PubMed PMID: 20978719.  27/28: de Oliveira MT, Pereira JR, Ghizoni JS, Bittencourt ST, Molina GO. Effects from exposure to dental amalgam on systemic mercury levels in patients and dental school students. Photomed Laser Surg. 2010 Oct;28 Suppl 2:S111-4. doi: 10.1089/pho.2009.2656. Epub 2010 Oct 7. PubMed PMID: 20929387.  28/29: Ansari G, Ranjpour M. Mineral trioxide aggregate and formocresol pulpotomy of primary teeth: a 2-year follow-up. Int Endod J. 2010 May;43(5):413-8. doi: 10.1111/j.1365-2591.2010.01695.x. PubMed PMID: 20518934.  29/30: Simratvir M, Singh N, Chopra S, Thomas AM. Efficacy of 10% Povidone Iodine in children affected with early childhood caries: an in vivo study. J Clin Pediatr Dent. 2010 Spring;34(3):233-8. PubMed PMID: 20578661.  30/31: Gupta M, Pandit IK, Srivastava N, Gugnani N. Comparative evaluation of 2% sodium fluoride iontophoresis and other cavity liners beneath silver amalgam restorations. J Indian Soc Pedod Prev Dent. 2010 Apr-Jun;28(2):68-72. doi: 10.4103/0970-4388.66738. PubMed PMID: 20660970.  31/32: Daou MH, Tavernier B, Meyer JM. Two-year clinical evaluation of three restorative materials in primary molars. J Clin Pediatr Dent. 2009 Fall;34(1):53-8. PubMed PMID: 19953810.  32/67. Sonmez D(1), Duruturk L. Success rate of calcium hydroxide pulpotomy in primary molars restored with amalgam and stainless steel crowns. Br Dent J. 2010 May 8;208(9):E18; discussion 408-9. doi: 10.1038/sj.bdj.2010.446. |
| Citations of laboratory studies | 1: Gomes GM, Gomes OM, Gomes JC, Loguercio AD, Calixto AL, Reis A. Evaluation of different restorative techniques for filling flared root canals: fracture resistance and bond strength after mechanical fatigue. J Adhes Dent. 2014 Jun;16(3):267-76. doi: 10.3290/j.jad.a31940. PubMed PMID: 24779026.  2: Cheetham JJ, Palamara JE, Tyas MJ, Burrow MF. A comparison of the shear bond strength and failure mode to metals of unsupported and supported luting cement specimens. J Adhes Dent. 2014 Jun;16(3):251-60. doi: 10.3290/j.jad.a31345. PubMed PMID: 24479119.  3: Zesewitz TF, Knauber AW, Northdurft FP. Fracture resistance of a selection of full-contour all-ceramic crowns: an in vitro study. Int J Prosthodont. 2014 May-Jun;27(3):264-6. doi: 10.11607/ijp.3815. PubMed PMID: 24905268.  4: Iliadi A, Baumgartner S, Athanasiou AE, Eliades T, Eliades G. Effect of intraoral aging on the setting status of resin composite and glass ionomer orthodontic adhesives. Am J Orthod Dentofacial Orthop. 2014 Apr;145(4):425-33. doi: 10.1016/j.ajodo.2013.11.020. PubMed PMID: 24703280.  5: Altamimi AM, Tripodakis AP, Eliades G, Hirayama H. Comparison of fracture resistance and fracture characterization of bilayered zirconia/fluorapatite and monolithic lithium disilicate all ceramic crowns. Int J Esthet Dent. 2014 Spring;9(1):98-110. PubMed PMID: 24757702.  6: De Conto F, Ericson Flores M, Cucco C, Prates Soares Zerbinati L, Dedavid BA, Gerhardt De Oliveira M. A comparative study of materials and storage modes for human teeth in apicoectomy: scanning electron microscopy analysis. Minerva Stomatol. 2014 Apr;63(4):95-102. PubMed PMID: 24705039.  7: Yap J, Walsh LJ, Naser-Ud Din S, Ngo H, Manton DJ. Evaluation of a novel approach in the prevention of white spot lesions around orthodontic brackets. Aust Dent J. 2014 Mar;59(1):70-80. doi: 10.1111/adj.12142. Epub 2014 Feb 14. PubMed PMID: 24528001.  8: Cakan U, Gultekin P, Guncu MB, Canay S. Effect of screw access channel filling materials on uniaxial retentive force of cement-retained implant restorations. Aust Dent J. 2014 Mar;59(1):65-9. doi: 10.1111/adj.12148. Epub 2014 Feb 4. PubMed PMID: 24494783.  9: Zmener O, Pameijer CH, Hernández S. Resistance against bacterial leakage of four luting agents used for cementation of complete cast crowns. Am J Dent. 2014 Feb;27(1):51-5. PubMed PMID: 24902406.  10: El-Negoly SA, El-Fallal AA, El-Sherbiny IM. A new modification for improving shear bond strength and other mechanical properties of conventional glass-ionomer restorative materials. J Adhes Dent. 2014 Feb;16(1):41-7. doi: 10.3290/j.jad.a30541. PubMed PMID: 24000334.  11: Colucci V, de Araújo Loiola AB, da Motta DS, do Amaral FL, Pécora JD, Corona SA. Influence of long-term water storage and thermocycling on shear bond strength of glass-ionomer cement to Er:YAG laser-prepared dentin. J Adhes Dent. 2014 Feb;16(1):35-9. doi: 10.3290/j.jad.a30539. PubMed PMID: 24000332.  12: Ráth G, Katona G, Bakó P, Török L, Révész P, Tóth E, Gerlinger I. Application of ionomer cement onto the stapedial footplate: impact on the perilymphatic aluminum level. Laryngoscope. 2014 Feb;124(2):541-4. doi: 10.1002/lary.24289. Epub 2013 Jul 9. PubMed PMID: 23818210.  13: Luczaj-Cepowicz E, Marczuk-Kolada G, Zalewska A, Pawińska M, Leszczyńska K. Antibacterial activity of selected glass ionomer cements. Postepy Hig Med Dosw (Online). 2014 Jan 22;68:23-8. doi: 10.5604/17322693.1086069. PubMed PMID: 24491892.  14: Santos RL, Moura Mde F, Carvalho FG, Guênes GM, Alves PM, Pithon MM. Histological analysis of biocompatibility of ionomer cements with an acid-base reaction. Braz Oral Res. 2014 Jan-Feb;28(1). doi: 10.1590/S1806-83242014.50000003. PubMed PMID: 25006620.  15: Cheetham JJ, Palamara JE, Tyas MJ, Burrow MF. Evaluation of the interfacial work of fracture of glass-ionomer cements bonded to dentin. J Mech Behav Biomed Mater. 2014 Jan;29:427-37. doi: 10.1016/j.jmbbm.2013.09.020. Epub 2013 Oct 10. PubMed PMID: 24189324.  16: Zoergiebel J, Ilie N. An in vitro study on the maturation of conventional glass ionomer cements and their interface to dentin. Acta Biomater. 2013 Dec;9(12):9529-37. doi: 10.1016/j.actbio.2013.08.010. Epub 2013 Aug 13. PubMed PMID: 23954325.  17: Scaminaci Russo D, Iuliano V, Franchi L, Ferrari M, Giachetti L. Adhesion to primary dentin: microshear bond strength and scanning electron microscopic observation. Am J Dent. 2013 Dec;26(6):341-6. PubMed PMID: 24640439.  18: Bagheri R, Taha NA, Azar MR, Burrow MF. Effect of G-Coat Plus on the mechanical properties of glass-ionomer cements. Aust Dent J. 2013 Dec;58(4):448-53. doi: 10.1111/adj.12122. PubMed PMID: 24320901.  19: Rodriguez IA, Ferrara CA, Campos-Sanchez F, Alaminos M, Echevarría JU, Campos A. An in vitro biocompatibility study of conventional and resin-modified glass ionomer cements. J Adhes Dent. 2013 Dec;15(6):541-6. doi: 10.3290/j.jad.a29588. PubMed PMID: 23593641.  20: Kim IY, Ohtsuki C, Coughlan A, Placek L, Wren AW, Towler MR. Characteristics of glass ionomer cements composed of glass powders in CaO-SrO-ZnO-SiO₂ system prepared by two different synthetic routes. J Mater Sci Mater Med. 2013 Dec;24(12):2677-82. doi: 10.1007/s10856-013-5017-z. Epub 2013 Aug 7. PubMed PMID: 23918526.  21: Barros AA, Alves A, Nunes C, Coimbra MA, Pires RA, Reis RL. Carboxymethylation of ulvan and chitosan and their use as polymeric components of bone cements. Acta Biomater. 2013 Nov;9(11):9086-97. doi: 10.1016/j.actbio.2013.06.036. Epub 2013 Jun 29. PubMed PMID: 23816652.  22: Enan ET, Hammad SM. Microleakage under orthodontic bands cemented with nano-hydroxyapatite-modified glass ionomer. Angle Orthod. 2013 Nov;83(6):981-6. doi: 10.2319/022013-147.1. Epub 2013 Jun 7. PubMed PMID: 23745977.  23: Baghalian A, Nakhjavani YB, Hooshmand T, Motahhary P, Bahramian H. Microleakage of Er:YAG laser and dental bur prepared cavities in primary teeth restored with different adhesive restorative materials. Lasers Med Sci. 2013 Nov;28(6):1453-60. doi: 10.1007/s10103-012-1222-0. Epub 2012 Nov 8. PubMed PMID: 23135785.  24: Hosoya Y, Ando S, Otani H, Yukinari T, Miyazaki M, Garcia-Godoy F. Ability of barrier coat S-PRG coating to arrest artificial enamel lesions in primary teeth. Am J Dent. 2013 Oct;26(5):286-90. PubMed PMID: 24479282.  25: Ferracane JL, Cooper PR, Smith AJ. Dentin matrix component solubilization by solutions of pH relevant to self-etching dental adhesives. J Adhes Dent. 2013 Oct;15(5):407-12. doi: 10.3290/j.jad.a29536. PubMed PMID: 23560260.  26: Prabhakar AR, Prahlad D, Kumar SR. Antibacterial activity, fluoride release, and physical properties of an antibiotic-modified glass ionomer cement. Pediatr Dent. 2013 Sep-Oct;35(5):411-5. PubMed PMID: 24290552.  27: Garbui BU, de Azevedo CS, Zezell DM, Aranha AC, Matos AB. Er,Cr:YSGG laser dentine conditioning improves adhesion of a glass ionomer cement. Photomed Laser Surg. 2013 Sep;31(9):453-60. doi: 10.1089/pho.2013.3546. PubMed PMID: 24047223.  28: Rirattanapong P, Vongsavan K, Surarit R. Microleakage of two fluoride-releasing sealants when applied following saliva contamination. Southeast Asian J Trop Med Public Health. 2013 Sep;44(5):931-4. PubMed PMID: 24437329.  29: Ferreira L, Pedrini D, Okamoto AC, Jardim Júnior EG, Henriques TA, Cannon M, Delbem AC. Biochemical and microbiological characteristics of in situ biofilm formed on materials containing fluoride or amorphous calcium phosphate. Am J Dent. 2013 Aug;26(4):207-13. PubMed PMID: 24693631.  30: Selimović-Dragaš M, Hasić-Branković L, Korać F, Đapo N, Huseinbegović A, Kobašlija S, Lekić M, Hatibović-Kofman Š. In vitro fluoride release from a different kind of conventional and resin modified glass-ionomer cements. Bosn J Basic Med Sci. 2013 Aug;13(3):197-202. PubMed PMID: 23988173.  31: Pereira TB, Jansen WC, Pithon MM, Souki BQ, Tanaka OM, Oliveira DD. Effects of enamel deproteinization on bracket bonding with conventional and resin-modified glass ionomer cements. Eur J Orthod. 2013 Aug;35(4):442-6. doi: 10.1093/ejo/cjs006. Epub 2012 Feb 29. PubMed PMID: 22379131.  32: Cvikl B, Filipowitsch R, Wernisch J, Raabe M, Gruber R, Moritz A. Immediate shear bond strengths of a composite, a compomer and a glass ionomer to a ceramic substrate. J Adhes Dent. 2013 Aug;15(4):385-91. doi: 10.3290/j.jad.a28879. PubMed PMID: 23534021.  33: Pereira JR, Lins do Valle A, Ghizoni JS, Lorenzoni FC, Ramos MB, Dos Reis Só MV. Push-out bond strengths of different dental cements used to cement glass fiber posts. J Prosthet Dent. 2013 Aug;110(2):134-40. doi: 10.1016/S0022-3913(13)60353-4. Erratum in: J Prosthet Dent. 2013 Sep;110(3):235. Barbosa, Marcelo Ramos [corrected to Ramos, Marcelo Barbosa]. PubMed PMID: 23929375.  34: Torres-Sánchez C, Montoya-Salazar V, Córdoba P, Vélez C, Guzmán-Duran A, Gutierrez-Pérez JL, Torres-Lagares D. Fracture resistance of endodontically treated teeth restored with glass fiber reinforced posts and cast gold post and cores cemented with three cements. J Prosthet Dent. 2013 Aug;110(2):127-33. doi: 10.1016/S0022-3913(13)60352-2. PubMed PMID: 23929374.  35: Korkmaz FM, Tüzüner T, Baygin O, Buruk CK, Durkan R, Bagis B. Antibacterial activity, surface roughness, flexural strength, and solubility of conventional luting cements containing chlorhexidine diacetate/cetrimide mixtures. J Prosthet Dent. 2013 Aug;110(2):107-15. doi: 10.1016/S0022-3913(13)60349-2. PubMed PMID: 23929372.  36: Tamilselvam S, Divyanand MJ, Neelakantan P. Biocompatibility of a conventional glass ionomer, ceramic reinforced glass ionomer, giomer and resin composite to fibroblasts: in vitro study. J Clin Pediatr Dent. 2013 Summer;37(4):403-6. PubMed PMID: 24046990.  37: Camilleri J. Investigation of Biodentine as dentine replacement material. J Dent. 2013 Jul;41(7):600-10. doi: 10.1016/j.jdent.2013.05.003. Epub 2013 May 15. PubMed PMID: 23685034.  38: Dickey BT, Kehoe S, Boyd D. Novel adaptations to zinc-silicate glass polyalkenoate cements: the unexpected influences of germanium based glasses on handling characteristics and mechanical properties. J Mech Behav Biomed Mater. 2013 Jul;23:8-21. doi: 10.1016/j.jmbbm.2013.03.012. Epub 2013 Apr 8. PubMed PMID: 23648365.  39: Imbery TA, Namboodiri A, Duncan A, Amos R, Best AM, Moon PC. Evaluating dentin surface treatments for resin-modified glass ionomer restorative materials. Oper Dent. 2013 Jul-Aug;38(4):429-38. doi: 10.2341/12-162-L. Epub 2012 Oct 22. PubMed PMID: 23088188.  40: Barbić MR, Segović S, Baraba A, Ribarić SP, Katunarić M, Anić I. Microleakage along glass-fibre posts cemented with three different materials after cyclic loading: a pilot study. Coll Antropol. 2013 Jun;37(2):431-5. PubMed PMID: 23940985.  41: Lim BS, Cheng Y, Lee SP, Ahn SJ. Chlorhexidine release from orthodontic adhesives after topical chlorhexidine treatment. Eur J Oral Sci. 2013 Jun;121(3 Pt 1):211-7. doi: 10.1111/eos.12033. Epub 2013 Mar 23. PubMed PMID: 23659245.  42: Tirali RE, Çehreli SB, Yazici R, Yalçinkaya Z. Effect of two anti-erosion pastes on surface roughness of different restorative materials. Eur J Paediatr Dent. 2013 Jun;14(2):135-9. PubMed PMID: 23758464.  43: Kuter B, Eden E, Yildiz H. The effect of heat on the mechanical properties of glass ionomer cements. Eur J Paediatr Dent. 2013 Jun;14(2):90-4. PubMed PMID: 23758455.  44: Chen YZ, Lü XY, Liu GD. A novel root-end filling material based on hydroxyapatite, tetracalcium phosphate and polyacrylic acid. Int Endod J. 2013 Jun;46(6):556-64. doi: 10.1111/iej.12028. Epub 2012 Nov 28. PubMed PMID: 23190302.  45: Ferla Jde O, Rodrigues JA, Arrais CA, Aranha AC, Cassoni A. Influence of photo-activation source on enamel demineralization around restorative materials. Braz Oral Res. 2013 May-Jun;27(3):286-92. PubMed PMID: 23459770.  46: Hitij T, Fidler A. Radiopacity of dental restorative materials. Clin Oral Investig. 2013 May;17(4):1167-77. doi: 10.1007/s00784-012-0797-y. Epub 2012 Jul 24. PubMed PMID: 22824916.  47: Fatima N, Ali Abidi SY, Qazi FU, Jat SA. Effectiveness of commonly available surface protecting agents in maintaining microhardness of two cements. J Coll Physicians Surg Pak. 2013 May;23(5):315-8. doi: 05.2013/JCPSP.314318. PubMed PMID: 23673167.  48: Zalizniak I, Palamara JE, Wong RH, Cochrane NJ, Burrow MF, Reynolds EC. Ion  release and physical properties of CPP-ACP modified GIC in acid solutions. J Dent. 2013 May;41(5):449-54. doi: 10.1016/j.jdent.2013.02.003. Epub 2013 Feb 21. PubMed PMID: 23438415.  49: Davis GR, Evershed AN, Mills D. Quantitative high contrast X-ray microtomography for dental research. J Dent. 2013 May;41(5):475-82. doi: 10.1016/j.jdent.2013.01.010. Epub 2013 Feb 1. PubMed PMID: 23380275.  50: Wren AW, Hansen JP, Hayakawa S, Towler MR. Aluminium-free glass polyalkenoate cements: ion release and in vitro antibacterial efficacy. J Mater Sci Mater Med. 2013 May;24(5):1167-78. doi: 10.1007/s10856-013-4880-y. Epub 2013 Feb 6. PubMed PMID: 23386211.  51: Ioannidis K, Beltes P, Lambrianidis T, Kapagiannidis D, Karagiannis V. Crown discoloration induced by endodontic sealers: spectrophotometric measurement of Commission International de I'Eclairage's L*, a*, b* chromatic parameters. Oper Dent. 2013 May-Jun;38(3):E1-12. doi: 10.2341/11-266-L. Epub 2013 Feb 7. PubMed PMID: 23391031.  52: Dursun E, Le Goff S, Ruse DN, Attal JP. Effect of chlorhexidine application on the long-term shear bond strength to dentin of a resin-modified glass ionomer. Oper Dent. 2013 May-Jun;38(3):275-81. doi: 10.2341/11-501-L. Epub 2012 Oct 23. PubMed PMID: 23092171.  53: Karaman E, Ozgunaltay G. Cuspal deflection in premolar teeth restored using current composite resins with and without resin-modified glass ionomer liner. Oper Dent. 2013 May-Jun;38(3):282-9. doi: 10.2341/11-400-L. Epub 2012 Oct 23. PubMed PMID: 23092141.  54: Zhou HM, Shen Y, Wang ZJ, Li L, Zheng YF, Häkkinen L, Haapasalo M. In vitro cytotoxicity evaluation of a novel root repair material. J Endod. 2013 Apr;39(4):478-83. doi: 10.1016/j.joen.2012.11.026. Epub 2013 Jan 11. PubMed PMID: 23522540.  55: da Rocha PV, Freitas MA, de Morais Alves da Cunha T. Influence of screw access on the retention of cement-retained implant prostheses. J Prosthet Dent. 2013 Apr;109(4):264-8. doi: 10.1016/S0022-3913(13)60055-4. PubMed PMID: 23566608.  56: Chee WW, Duncan J, Afshar M, Moshaverinia A. Evaluation of the amount of excess cement around the margins of cement-retained dental implant restorations: the effect of the cement application method. J Prosthet Dent. 2013 Apr;109(4):216-21. doi: 10.1016/S0022-3913(13)60047-5. PubMed PMID: 23566601.  57: Gomes FO, Pires RA, Reis RL. Aluminum-free glass-ionomer bone cements with enhanced bioactivity and biodegradability. Mater Sci Eng C Mater Biol Appl. 2013 Apr 1;33(3):1361-70. doi: 10.1016/j.msec.2012.12.037. Epub 2012 Dec 13. PubMed PMID: 23827583.  58: Romeed SA, Dunne SM. Stress analysis of different post-luting systems: a three-dimensional finite element analysis. Aust Dent J. 2013 Mar;58(1):82-8. doi: 10.1111/adj.12030. Epub 2013 Jan 30. PubMed PMID: 23441796.  59: Aguiar DA, Ritter DE, Rocha R, Locks A, Borgatto AF. Evaluation of mechanical properties of five cements for orthodontic band cementation. Braz Oral Res. 2013 Mar-Apr;27(2):136-41. Epub 2013 Mar 1. PubMed PMID: 23459769.  60: de Mattos Pimenta Vidal C, Pavan S, Briso AL, Bedran-Russo AK. Effects of three restorative techniques in the bond strength and nanoleakage at gingival wall of Class II restorations subjected to simulated aging. Clin Oral Investig. 2013 Mar;17(2):627-33. doi: 10.1007/s00784-012-0748-7. Epub 2012 May 11. PubMed PMID: 22576325.  61: Engels J, Schubert O, Güth JF, Hoffmann M, Jauernig C, Erdelt K, Stimmelmayr M, Beuer F. Wear behavior of different double-crown systems. Clin Oral Investig. 2013 Mar;17(2):503-10. doi: 10.1007/s00784-012-0746-9. Epub 2012 May 10. PubMed PMID: 22573245.  62: Zoergiebel J, Ilie N. Evaluation of a conventional glass ionomer cement with new zinc formulation: effect of coating, aging and storage agents. Clin Oral Investig. 2013 Mar;17(2):619-26. doi: 10.1007/s00784-012-0733-1. Epub 2012 May 2. PubMed PMID: 22549662.  63: Ozdemir-Ozenen D, Sungurtekin E, Issever H, Sandalli N. Surface roughness of fluoride-releasing restorative materials after topical fluoride application. Eur J Paediatr Dent. 2013 Mar;14(1):68-72. PubMed PMID: 23597225.  64: Erdemir U, Yildiz E, Eren MM, Ozel S. Surface hardness evaluation of different composite resin materials: influence of sports and energy drinks immersion after a short-term period. J Appl Oral Sci. 2013 Mar-Apr;21(2):124-31. doi: 10.1590/1678-7757201302185. PubMed PMID: 23739850; PubMed Central PMCID: PMC3881868.  65: Coughlan A, Breed SM, Ashraf C, Cardinale JA, Hall MM, Towler MR. Does elevating silver content in zinc-based glass polyalkenoate cements increase their antibacterial efficacy against two common bacteria using the agar gel diffusion method? J Biomater Appl. 2013 Mar;27(7):840-7. doi: 10.1177/0885328211427775. Epub 2012 Jan 19. PubMed PMID: 22262577.  66: Calixto LR, Tonetto MR, Pinto SC, Barros ED, Borges AH, Lima FV, de Andrade MF, Bandéca MC. Degree of conversion and hardness of two different systems of the Vitrebond™ glass ionomer cement light cured with blue LED. J Contemp Dent Pract. 2013 Mar 1;14(2):244-9. PubMed PMID: 23811653.  67: Menne-Happ U, Ilie N. Effect of gloss and heat on the mechanical behaviour of a glass carbomer cement. J Dent. 2013 Mar;41(3):223-30. doi: 10.1016/j.jdent.2012.11.005. Epub 2012 Nov 19. PubMed PMID: 23174652.  68: Turgut S, Bagis B. Effect of resin cement and ceramic thickness on final color of laminate veneers: an in vitro study. J Prosthet Dent. 2013  Mar;109(3):179-86. doi: 10.1016/S0022-3913(13)60039-6. PubMed PMID: 23522367.  69: Khoroushi M, Mousavinasab SM, Keshani F, Hashemi S. Effect of resin-modified glass ionomer containing bioactive glass on the flexural strength and morphology of demineralized dentin. Oper Dent. 2013 Mar-Apr;38(2):E1-10. doi: 10.2341/11-325-L. Epub 2012 Oct 25. PubMed PMID: 23098663.  70: Sabatini C, Patel M, D'Silva E. In vitro shear bond strength of three self-adhesive resin cements and a resin-modified glass ionomer cement to various prosthodontic substrates. Oper Dent. 2013 Mar-Apr;38(2):186-96. doi: 10.2341/11-317-L. Epub 2012 Aug 22. PubMed PMID: 22913244.  71: Apostolović M, Kalicanin B, Igić M, Tricković-Janjić O, Surdilović D, Kostadinović L, Stojković B, Velimirović D. Migration of fluoride ions from the permanent teeth into saliva in children with glass ionomer cement restorations: an in vitro study. Vojnosanit Pregl. 2013 Mar;70(3):279-83. PubMed PMID: 23607239.  72: Carvalho CN, Bauer J, Ferrari PH, Souza SF, Soares SP, Loguercio AD, Bombana AC. Influence of calcium hydroxide intracanal medication on bond strength of two endodontic resin-based sealers assessed by micropush-out test. Dent Traumatol. 2013 Feb;29(1):73-6. doi: 10.1111/j.1600-9657.2011.01109.x. Epub 2012 Jan 12. PubMed PMID: 22236169.  73: de Castilho AR, Duque C, Negrini Tde C, Sacono NT, de Paula AB, de Souza Costa CA, Spolidório DM, Puppin-Rontani RM. In vitro and in vivo investigation of the biological and mechanical behaviour of resin-modified glass-ionomer cement containing chlorhexidine. J Dent. 2013 Feb;41(2):155-63. doi: 10.1016/j.jdent.2012.10.014. Epub 2012 Oct 30. PubMed PMID: 23123495.  74: Öztürk E, Bolay Ş, Hickel R, Ilie N. Shear bond strength of porcelain laminate veneers to enamel, dentine and enamel-dentine complex bonded with different adhesive luting systems. J Dent. 2013 Feb;41(2):97-105. doi: 10.1016/j.jdent.2012.04.005. Epub 2012 Apr 19. PubMed PMID: 22521701.  75: Wren AW, Coughlan A, Laffir FR, Towler MR. Comparison of a SiO(2)-CaO-ZnO-SrO glass polyalkenoate cement to commercial dental materials: glass structure and physical properties. J Mater Sci Mater Med. 2013 Feb;24(2):271-80. doi: 10.1007/s10856-012-4813-1. Epub 2012 Nov 22. PubMed PMID: 23179999.  76: Singh P, Paul J, Al-Khuraif AA, Vellappally S, Halawany HS, Hashim M, Abraham NB, Jacob V, Thavarajah R. Sealing ability of mineral trioxide aggregate, calcium phosphate cement, and glass ionomer cement in the repair of furcation perforations. Acta Medica (Hradec Kralove). 2013;56(3):97-103. PubMed PMID: 24592746.  77: Ceylan G, Dede DÖ, Külünk S, Ongöz Dede F. Effects of refractive index solutions on the color of different luting cements. Acta Odontol Scand. 2013 Jan;71(1):88-91. doi: 10.3109/00016357.2011.654244. Epub 2012 Feb 3. PubMed PMID: 22300162.  78: Fabián Molina G, Cabral RJ, Mazzola I, Brain Lascano L, Frencken JE. Biaxial flexural strength of high-viscosity glass-ionomer cements heat-cured with an LED lamp during setting. Biomed Res Int. 2013;2013:838460. doi: 10.1155/2013/838460. Epub 2013 Jun 12. PubMed PMID: 23841095; PubMed Central PMCID: PMC3694373.  79: Royer K, Liu XJ, Zhu Q, Malmstrom H, Ren YF. Apical and root canal space sealing abilities of resin and glass ionomer-based root canal obturation systems. Chin J Dent Res. 2013;16(1):47-53. PubMed PMID: 23878826.  80: Shimazu K, Ogata K, Karibe H. Evaluation of the caries-preventive effect of three orthodontic band cements in terms of fluoride release, retentiveness, and microleakage. Dent Mater J. 2013;32(3):376-80. PubMed PMID: 23718996.  81: Dionysopoulos D, Koliniotou-Koumpia E, Helvatzoglou-Antoniades M, Kotsanos N. Fluoride release and recharge abilities of contemporary fluoride-containing restorative materials and dental adhesives. Dent Mater J. 2013;32(2):296-304. PubMed PMID: 23538766.  82: Tymofiyeva O, Vaegler S, Rottner K, Boldt J, Hopfgartner AJ, Proff PC, Richter EJ, Jakob PM. Influence of dental materials on dental MRI. Dentomaxillofac Radiol. 2013;42(6):20120271. doi: 10.1259/dmfr.20120271. Epub 2013 Apr 22. PubMed PMID: 23610088; PubMed Central PMCID: PMC3667526.  83: Lachowski KM, Botta SB, Lascala CA, Matos AB, Sobral MA. Study of the radio-opacity of base and liner dental materials using a digital radiography system. Dentomaxillofac Radiol. 2013;42(2):20120153. doi: 10.1259/dmfr.20120153. PubMed PMID: 23393292; PubMed Central PMCID: PMC3699023.  84: Portela A, Vasconcelos M, Fernandes MH, Garcia M, Silva A, Gabriel J, Gartner F, Amorim I, Cavalheiro J. Highly focalised thermotherapy using a ferromagnetic cement in the treatment of a melanoma mouse model by low temperature hyperthermia. Int J Hyperthermia. 2013;29(2):121-32. doi: 10.3109/02656736.2013.767478. PubMed PMID: 23418916.  85: Pette GA, Ganeles J, Norkin FJ. Radiographic appearance of commonly used cements in implant dentistry. Int J Periodontics Restorative Dent. 2013 Jan-Feb;33(1):61-8. PubMed PMID: 23342348.    86: Garg P, Gupta G, Prithviraj DR, Pujari M. Retentiveness of various luting agents used with implant-supported prostheses: a preliminary in vitro study. Int J Prosthodont. 2013 Jan-Feb;26(1):82-4. PubMed PMID: 23342339.  87: Molina GF, Cabral RJ, Mazzola I, Lascano LB, Frencken JE. Mechanical performance of encapsulated restorative glass-ionomer cements for use with Atraumatic Restorative Treatment (ART). J Appl Oral Sci. 2013;21(3):243-9. doi:  10.1590/1679-775720130129. PubMed PMID: 23857657; PubMed Central PMCID: PMC3881905.  88: Pacifici E, Chazine M, Vichi A, Grandini S, Goracci C, Ferrari M. Shear-bond strength of a new self-adhering flowable restorative material to dentin of primary molars. J Clin Pediatr Dent. 2013 Winter;38(2):149-54. PubMed PMID: 24683779.  89: Tapsir Z, Aly Ahmed HM, Luddin N, Husein A. Sealing ability of various restorative materials as coronal barriers between endodontic appointments. J Contemp Dent Pract. 2013 Jan 1;14(1):47-50. PubMed PMID: 23579892.  90: Rao KS, Reddy TP, Yugandhar G, Kumar BS, Reddy SN, Babu DA. Comparison of shear bond strength of resin reinforced chemical cure glass ionomer, conventional chemical cure glass ionomer and chemical cure composite resin in direct bonding systems: an in vitro study. J Contemp Dent Pract. 2013 Jan 1;14(1):21-5. PubMed PMID: 23579887.  91: Castillo-Oyagüe R, Lynch CD, Turrión AS, López-Lozano JF, Torres-Lagares D, Suárez-García MJ. Misfit and microleakage of implant-supported crown copings obtained by laser sintering and casting techniques, luted with glass-ionomer, resin cements and acrylic/urethane-based agents. J Dent. 2013 Jan;41(1):90-6. doi: 10.1016/j.jdent.2012.09.014. Epub 2012 Sep 26. PubMed PMID: 23022105.  92: Lenzi TL, Bonifácio CC, Bönecker M, Amerongen WE, Nogueira FN, Raggio DP. Flowable glass ionomer cement layer bonding to sound and carious primary dentin. J Dent Child (Chic). 2013 Jan-Apr;80(1):20-4. PubMed PMID: 23595240.  93: de Souza-e-Silva CM, Parisotto TM, Steiner-Oliveira C, Kamiya RU, Rodrigues LK, Nobre-dos-Santos M. Carbon dioxide laser and bonding materials reduce enamel demineralization around orthodontic brackets. Lasers Med Sci. 2013 Jan;28(1):111-8. doi: 10.1007/s10103-012-1076-5. Epub 2012 Mar 23. PubMed PMID: 22441828.  94: Baeza-Robleto SJ, Villa-Negrete DM, García-Contreras R, Scougall-Vílchis RJ, Guadarrama-Quiroz LJ, Robles-Bermeo NL. Effects of ultraviolet irradiation on the bond strength of a composite resin adhered to stainless steel crowns. Pediatr Dent. 2013 Jan-Feb;35(1):23-6. PubMed PMID: 23635891.  95: Bonifácio CC, Shimaoka AM, de Andrade AP, Raggio DP, van Amerongen WE, de Carvalho RC. Micro-mechanical bond strength tests for the assessment of the adhesion of GIC to dentine. Acta Odontol Scand. 2012 Dec;70(6):555-63. doi: 10.3109/00016357.2011.640280. Epub 2012 Jan 18. PubMed PMID: 22250948.  96: Bonifácio CC, Werner A, Kleverlaan CJ. Coating glass-ionomer cements with a nanofilled resin. Acta Odontol Scand. 2012 Dec;70(6):471-7. doi: 10.3109/00016357.2011.639307. Epub 2011 Dec 12. PubMed PMID: 22149968.  97: Gonzalez-Perez JC, Scougall-Vilchis RJ, Contreras-Bulnes R, De La Rosa-Gómez I, Uematsu S, Yamaguchi R. Adherence of Streptococcus mutans to orthodontic band cements. Aust Dent J. 2012 Dec;57(4):464-9. doi: 10.1111/adj.12004. Epub 2012 Oct 14. PubMed PMID: 23186572.  98: Gorseta K, Glavina D, Skrinjaric I. Influence of ultrasonic excitation and heat application on the microleakage of glass ionomer cements. Aust Dent J. 2012 Dec;57(4):453-7. doi: 10.1111/j.1834-7819.2012.01724.x. Epub 2012 Sep 11. PubMed PMID: 23186570.  99: Tuncer S, Demirci M, Schweikl H, Erguven M, Bilir A, Kara Tuncer A. Inhibition of cell survival, viability and proliferation by dentin adhesives after direct and indirect exposure in vitro. Clin Oral Investig. 2012 Dec;16(6):1635-46. doi: 10.1007/s00784-011-0669-x. Epub 2012 Jan 6. PubMed PMID: 22222515.  100: Gorseta K, Skrinjarić T, Glavina D. The effect of heating and ultrasound on the shear bond strength of glass ionomer cement. Coll Antropol. 2012 Dec;36(4):1307-12. PubMed PMID: 23390826.  101: Raskin A, Eschrich G, Dejou J, About I. In vitro microleakage of Biodentine as a dentin substitute compared to Fuji II LC in cervical lining restorations. J Adhes Dent. 2012 Dec;14(6):535-42. doi: 10.3290/j.jad.a25690. PubMed PMID: 22724110.  102: Samra AP, Ribeiro DG, Borges CP, Kossatz S. Influence of professional prophylaxis on reducing discoloration of different aesthetic restorative materials. J Dent. 2012 Dec;40 Suppl 2:e71-6. doi: 10.1016/j.jdent.2012.06.003. Epub 2012 Jun 17. PubMed PMID: 22713738.  103: Lee BN, Son HJ, Noh HJ, Koh JT, Chang HS, Hwang IN, Hwang YC, Oh WM. Cytotoxicity of newly developed ortho MTA root-end filling materials. J Endod. 2012 Dec;38(12):1627-30. doi: 10.1016/j.joen.2012.09.004. Epub 2012 Oct 13.  PubMed PMID: 23146650.  104: Abi-Rached Fde O, Fonseca RG, Haneda IG, de Almeida-Júnior AA, Adabo GL. The effect of different surface treatments on the shear bond strength of luting cements to titanium. J Prosthet Dent. 2012 Dec;108(6):370-6. doi: 10.1016/S0022-3913(12)60194-2. PubMed PMID: 23217469.  105: Selimović-Dragaš M, Huseinbegović A, Kobašlija S, Hatibović-Kofman S. A comparison of the in vitro cytotoxicity of conventional and resin modified glass ionomer cements. Bosn J Basic Med Sci. 2012 Nov;12(4):273-8. PubMed PMID: 23198945.  106: Dawood HF, Abdul Rahman N, Hassan R, Al-Juboori MJ. Shear bond strength comparison of two different adhesive systems. Dent Implantol Update. 2012 Nov;23(11):83-8. PubMed PMID: 23156078.  107: Suihkonen RW, Vandewalle KS, Dossett JM. Bond strength of resin-modified glass ionomer restorative materials using a no-rinse conditioner. Gen Dent. 2012 Nov-Dec;60(6):e413-7. PubMed PMID: 23220322.  108: Camargo CH, Fonseca MB, Carvalho AS, Camargo SE, Cardoso FG, Valera MC. Microhardness and sealing ability of materials used for root canal perforations. Gen Dent. 2012 Nov-Dec;60(6):e393-7. PubMed PMID: 23220318.  109: Khoroushi M, Mansoori-Karvandi T, Hadi S. The effect of pre-warming and delayed irradiation on marginal integrity of a resin-modified glass-ionomer. Gen Dent. 2012 Nov-Dec;60(6):e383-8. PubMed PMID: 23220316.  110: Souchois MW, Vieira RS. Effect of a glass ionomer cement and a fluoride varnish on cross-sectional microhardness values of artificial occlusal caries: in vitro study. Indian J Dent Res. 2012 Nov-Dec;23(6):732-7. doi: 10.4103/0970-9290.111248. PubMed PMID: 23649054.  111: Lewinstein I, Zenziper E, Block J, Kfir A. Incorporation of chlorhexidine diacetate in provisional cements: antimicrobial activity against Streptococcus mutans and the effect on tensile strength in vitro. Int Endod J. 2012 Nov;45(11):1010-7. doi: 10.1111/j.1365-2591.2012.02063.x. Epub 2012 May 8. PubMed  PMID: 22563792.  112: Pameijer CH. Crown retention with three resin-modified glass ionomer luting agents. J Am Dent Assoc. 2012 Nov;143(11):1218-22. PubMed PMID: 23115151.  113: Pamir T, Sen BH, Evcin O. Effects of etching and adhesive applications on the bond strength between composite resin and glass-ionomer cements. J Appl Oral Sci. 2012 Nov-Dec;20(6):636-42. PubMed PMID: 23329245; PubMed Central PMCID: PMC3881859.  114: Borges GA, Faria JS, Agarwal P, Spohr AM, Correr-Sobrinho L, Miranzi BA. In vitro marginal fit of three all-ceramic crown systems before and after cementation. Oper Dent. 2012 Nov-Dec;37(6):641-9. doi: 10.2341/11-012-L. Epub 2012 May 21. PubMed PMID: 22616924.  115: Catalbaş B, Kamak H, Demir A, Nur M, Hadimli HH. Antibacterial effects of several current orthodontic materials against Streptococcus mutans. West Indian Med J. 2012 Nov;61(8):821-5. PubMed PMID: 23757904.  116: Zainuddin N, Karpukhina N, Law RV, Hill RG. Characterisation of a remineralising Glass Carbomer® ionomer cement by MAS-NMR spectroscopy. Dent Mater. 2012 Oct;28(10):1051-8. doi: 10.1016/j.dental.2012.06.011. Epub 2012 Jul 27. PubMed PMID: 22841162.  117: Topaloglu-Ak A, Cogulu D, Ersin NK, Sen BH. Microhardness and surface roughness of glass ionomer cements after APF and TiF4 applications. J Clin Pediatr Dent. 2012 Fall;37(1):45-51. PubMed PMID: 23342566.  118: Son YH, Han CH, Kim S. Influence of internal-gap width and cement type on the retentive force of zirconia copings in pullout testing. J Dent. 2012 Oct;40(10):866-72. doi: 10.1016/j.jdent.2012.07.007. Epub 2012 Jul 20. PubMed PMID: 22819954.  119: Singhal R, Pathak A. Comparison of the fracture resistance of reattached  incisor tooth fragments using 4 different materials. J Indian Soc Pedod Prev Dent. 2012 Oct-Dec;30(4):310-6. doi: 10.4103/0970-4388.108927. PubMed PMID: 23514683.  120: Tiwari S, Nandlal B. Comparative evaluation of fluoride release from hydroxyapatite incorporated and conventional glass ionomer cement: an in vitro study. J Indian Soc Pedod Prev Dent. 2012 Oct-Dec;30(4):284-7. doi: 10.4103/0970-4388.108921. PubMed PMID: 23514678.  121: Carnaggio TV, Conrad R, Engelmeier RL, Gerngross P, Paravina R, Perezous L, Powers JM. Retention of CAD/CAM all-ceramic crowns on prefabricated implant abutments: an in vitro comparative study of luting agents and abutment surface area. J Prosthodont. 2012 Oct;21(7):523-8. doi: 10.1111/j.1532-849X.2012.00847.x. Epub 2012 Apr 1. PubMed PMID: 22469271.  122: Ghasemi A, Torabzadeh H, Mahdian M, Afkar M, Fazeli A, Akbarzadeh Baghban A. Effect of bonding application time on the microleakage of Class V sandwich restorations. Aust Dent J. 2012 Sep;57(3):334-8. doi: 10.1111/j.1834-7819.2012.01712.x. Epub 2012 Jul 6. PubMed PMID: 22924357.  123: Patil PG, Parkhedkar RD, Patil SP, Bhowmik HS. Comparative evaluation of effect of polymerizable and non-polymerizable desensitizing agents on crown-retentive-strength of zinc-phosphate, glass-ionomer and compomer cements. Eur J Prosthodont Restor Dent. 2012 Sep;20(3):102-10. PubMed PMID: 23101176.  124: Mehl C, Harder S, Shahriari A, Steiner M, Kern M. Influence of abutment height and thermocycling on retrievability of cemented implant-supported crowns. Int J Oral Maxillofac Implants. 2012 Sep-Oct;27(5):1106-15. PubMed PMID: 23057023.  125: Ali H, Maroli S. Glass ionomer cement as an orthodontic bonding agent. J Contemp Dent Pract. 2012 Sep 1;13(5):650-4. PubMed PMID: 23250169.  126: Carvalho FG, Sampaio CS, Fucio SB, Carlo HL, Correr-Sobrinho L, Puppin-Rontani RM. Effect of chemical and mechanical degradation on surface roughness of three glass ionomers and a nanofilled resin composite. Oper Dent. 2012 Sep-Oct;37(5):509-17. doi: 10.2341/10-406-L. Epub 2012 Mar 21. PubMed PMID: 22433031.  127: Weng Y, Howard L, Chong VJ, Sun J, Gregory RL, Xie D. A novel furanone-modified antibacterial dental glass ionomer cement. Acta Biomater. 2012 Aug;8(8):3153-60. doi: 10.1016/j.actbio.2012.04.038. Epub 2012 Apr 30. PubMed PMID: 22554887.  128: Mehl C, Harder S, Schwarz D, Steiner M, Vollrath O, Kern M. In vitro influence of ultrasonic stress, removal force preload and thermocycling on the retrievability of implant-retained crowns. Clin Oral Implants Res. 2012  Aug;23(8):930-7. doi: 10.1111/j.1600-0501.2011.02236.x. Epub 2011 Jul 4. PubMed  PMID: 21722192.  129: Özcan M, Mese A. Adhesion of conventional and simplified resin-based luting cements to superficial and deep dentin. Clin Oral Investig. 2012 Aug;16(4):1081-8. doi: 10.1007/s00784-011-0594-z. Epub 2011 Jul 21. PubMed PMID: 21833482.  130: Krämer N, Rudolph H, Garcia-Godoy F, Frankenberger R. Effect of thermo-mechanical loading on marginal quality and wear of primary molar crowns. Eur Arch Paediatr Dent. 2012 Aug;13(4):185-90. PubMed PMID: 22883357.  131: Rechenberg DK, Schriber M, Attin T. Bacterial leakage through temporary fillings in core buildup composite material - an in vitro study. J Adhes Dent. 2012 Aug;14(4):371-6. doi: 10.3290/j.jad.a22709. PubMed PMID: 22282750.  132: Taha NA, Palamara JE, Messer HH. Assessment of laminate technique using glass ionomer and resin composite for restoration of root filled teeth. J Dent. 2012 Aug;40(8):617-23. doi: 10.1016/j.jdent.2012.04.006. Epub 2012 Apr 20. PubMed PMID: 22521705.  133: Eid AA, Komabayashi T, Watanabe E, Shiraishi T, Watanabe I. Characterization of the mineral trioxide aggregate-resin modified glass ionomer cement interface in different setting conditions. J Endod. 2012 Aug;38(8):1126-9. doi: 10.1016/j.joen.2012.04.013. Epub 2012 Jun 17. PubMed PMID: 22794220; PubMed Central PMCID: PMC3739278.  134: Modareszadeh MR, Di Fiore PM, Tipton DA, Salamat N. Cytotoxicity and alkaline phosphatase activity evaluation of endosequence root repair material. J Endod. 2012 Aug;38(8):1101-5. doi: 10.1016/j.joen.2012.04.014. Epub 2012 Jun 8. PubMed PMID: 22794214.  135: Wren AW, Coughlan A, Placek L, Towler MR. Gallium containing glass polyalkenoate anti-cancerous bone cements: glass characterization and physical properties. J Mater Sci Mater Med. 2012 Aug;23(8):1823-33. doi: 10.1007/s10856-012-4624-4. Epub 2012 Jun 9. PubMed PMID: 22684625.  136: Cruz JB, Lenzi TL, Tedesco TK, Guglielmi Cde A, Raggio DP. Eroded dentin does not jeopardize the bond strength of adhesive restorative materials. Braz Oral Res. 2012 Jul-Aug;26(4):306-12. Epub 2012 Jun 19. PubMed PMID: 22714927.  137: Larsson C, El Madhoun S, Wennerberg A, Vult von Steyern P. Fracture strength of yttria-stabilized tetragonal zirconia polycrystals crowns with different design: an in vitro study. Clin Oral Implants Res. 2012 Jul;23(7):820-6. doi: 10.1111/j.1600-0501.2011.02224.x. Epub 2011 Jun 2. PubMed PMID: 21635559.  138: Santos MJ, Driessen CH, de Freitas AP, Rizkalla AS, Santos GC Jr. In vitro shear bond strength of resin-based luting cements to dentin. Gen Dent. 2012 Jul-Aug;60(4):e215-20. PubMed PMID: 22782054.  139: Moreau JL, Weir MD, Giuseppetti AA, Chow LC, Antonucci JM, Xu HH. Long-term mechanical durability of dental nanocomposites containing amorphous calcium phosphate nanoparticles. J Biomed Mater Res B Appl Biomater. 2012 Jul;100(5):1264-73. doi: 10.1002/jbm.b.32691. Epub 2012 Apr 19. PubMed PMID: 22514160; PubMed Central PMCID: PMC3373274.  140: Shimazu K, Ogata K, Karibe H. Caries-preventive effect of fissure sealant containing surface reaction-type pre-reacted glass ionomer filler and bonded by self-etching primer. J Clin Pediatr Dent. 2012 Summer;36(4):343-7. PubMed PMID: 23019829.  141: Pilo R, Nissan J, Shafir H, Shapira G, Alter E, Brosh T. The influence of long term water immersion on shear bond strength of amalgam repaired by resin composite and mediated by adhesives or resin modified glass ionomers. J Dent. 2012 Jul;40(7):594-602. doi: 10.1016/j.jdent.2012.04.001. Epub 2012 Apr 12. PubMed PMID: 22504527.  142: Bhalla M, Patel D, Shashikiran ND, Mallikarjuna RM, Nalawade TM, Reddy HK. Effect of light-emitting diode and halogen light curing on the micro-hardness of dental composite and resin-modified glass ionomer cement: an in vitro study. J Indian Soc Pedod Prev Dent. 2012 Jul-Sep;30(3):201-5. doi: 10.4103/0970-4388.105011. PubMed PMID: 23263422.  143: Oyagüe RC, Sánchez-Turrión A, López-Lozano JF, Montero J, Albaladejo A, Suárez-García MJ. Evaluation of fit of cement-retained implant-supported 3-unit structures fabricated with direct metal laser sintering and vacuum casting techniques. Odontology. 2012 Jul;100(2):249-53. doi: 10.1007/s10266-011-0050-1. Epub 2011 Nov 11. PubMed PMID: 22075754.  144: Lawson NC, Cakir D, Beck P, Ramp L, Burgess JO. Effect of light activation on resin-modified glass ionomer shear bond strength. Oper Dent. 2012 Jul-Aug;37(4):380-5. doi: 10.2341/11-212-L. Epub 2012 Feb 15. PubMed PMID: 22335299.  145: Naoum S, O'Regan J, Ellakwa A, Benkhart R, Swain M, Martin E. The effect of repeated fluoride recharge and storage media on bond durability of fluoride rechargeable Giomer bonding agent. Aust Dent J. 2012 Jun;57(2):178-83. doi: 10.1111/j.1834-7819.2012.01681.x. Epub 2012 Apr 4. PubMed PMID: 22624758.  146: Gulve MN, Gulve ND, Shinde R, Kolhe SJ. The effect of environmental pressure changes on the retentive strength of cements for orthodontic bands. Diving Hyperb Med. 2012 Jun;42(2):78-81. Erratum in: Diving Hyperb Med. 2012 Dec;42(4):236. PubMed PMID: 22828814.  147: Prabhakar AR, Sharma D, Sugandhan S. Comparative evaluation of the remineralising effects and surface microhardness of glass ionomer cement containing grape seed extract and casein phosphopeptide - amorphous calcium phosphate: an in vitro study. Eur Arch Paediatr Dent. 2012 Jun;13(3):138-43. PubMed PMID: 22652211.  148: dos Santos RL, Pithon MM, Martins FO, Romanos MT, Ruellas AC. Evaluation of cytotoxicity and degree of conversion of glass ionomer cements reinforced with resin. Eur J Orthod. 2012 Jun;34(3):362-6. doi: 10.1093/ejo/cjr009. Epub 2011 Apr 8. PubMed PMID: 21478300.  149: Xie KX, Wang XY, Gao XJ, Yuan CY, Li JX, Chu CH. Fracture resistance of root filled premolar teeth restored with direct composite resin with or without cusp coverage. Int Endod J. 2012 Jun;45(6):524-9. doi: 10.1111/j.1365-2591.2011.02005.x. Epub 2012 Jan 14. PubMed PMID: 22242600.  150: Pithon MM, Ferraz Cde S, de Oliveira Gdo C, Pereira TB, Oliveira DD, de Souza RA, de Freitas LM, dos Santos RL. Effect of 10% papain gel on enamel deproteinization before bonding procedure. Angle Orthod. 2012 May;82(3):541-5. doi: 10.2319/062911-423.1. Epub 2011 Nov 11. PubMed PMID: 22077189.  151: Cheng L, Weir MD, Xu HH, Kraigsley AM, Lin NJ, Lin-Gibson S, Zhou X. Antibacterial and physical properties of calcium-phosphate and calcium-fluoride nanocomposites with chlorhexidine. Dent Mater. 2012 May;28(5):573-83. doi: 10.1016/j.dental.2012.01.006. Epub 2012 Feb 6. PubMed PMID: 22317794; PubMed Central PMCID: PMC3322264.  152: Stawarczyk B, Ozcan M, Hämmerle CH, Roos M. The fracture load and failure types of veneered anterior zirconia crowns: an analysis of normal and Weibull distribution of complete and censored data. Dent Mater. 2012 May;28(5):478-87. doi: 10.1016/j.dental.2011.11.023. Epub 2011 Dec 22. PubMed PMID: 22196897.  153: Khoroushi M, Karvandi TM, Kamali B, Mazaheri H. Marginal microleakage of resin-modified glass-ionomer and composite resin restorations: effect of using etch-and-rinse and self-etch adhesives. Indian J Dent Res. 2012 May-Jun;23(3):378-83. doi: 10.4103/0970-9290.102234. PubMed PMID: 23059577.  154: Jha P, Jha M. Retention of fiber posts in different dentin regions: an in vitro study. Indian J Dent Res. 2012 May-Jun;23(3):337-40. doi: 10.4103/0970-9290.102219. PubMed PMID: 23059569.  155: Gunjal S, Nagesh L, Raju HG. Comparative evaluation of marginal integrity of  glass ionomer and resin based fissure sealants using invasive and non-invasive techniques: an in vitro study. Indian J Dent Res. 2012 May-Jun;23(3):320-5. doi: 10.4103/0970-9290.102214. PubMed PMID: 23059566.  156: Chaudhari D, Sung EC, Paranjpe A, Jewett A. Novel strategies to enhance survival and growth of pulp cells after dental restorations. J Calif Dent Assoc. 2012 May;40(5):409-17. PubMed PMID: 22685948.  157: Stona P, Bertella SM, Rockenbach MI, Holderbaum RM, Weber JB. Radiopacities of glass ionomer cements measured with direct digital radiographic system. J Dent Child (Chic). 2012 May-Aug;79(2):59-62. PubMed PMID: 22828759.  158: Abdelaziz KM, Al-Qahtani NM, Al-Shehri AS, Abdelmoneam AM. Bonding quality of contemporary dental cements to sandblasted esthetic crown copings. J Investig Clin Dent. 2012 May;3(2):142-7. doi: 10.1111/j.2041-1626.2011.00106.x. Epub 2011 Oct 31. PubMed PMID: 22522951.  159: Thomas JT, Roberts HW, Diaz L, Bradley TG, Berzins DW. Effect of light-cure initiation time on polymerization efficiency and orthodontic bond strength with a resin-modified glass-ionomer. Orthod Craniofac Res. 2012 May;15(2):124-34. doi: 10.1111/j.1601-6343.2011.01531.x. Epub 2012 Mar 27. PubMed PMID: 22515188.  160: Vahid-Dastjerdi E, Borzabadi-Farahani A, Pourmofidi-Neistanak H, Amini N. An in-vitro assessment of weekly cumulative fluoride release from three glass ionomer cements used for orthodontic banding. Prog Orthod. 2012 May;13(1):49-56. doi: 10.1016/j.pio.2011.09.002. Epub 2011 Dec 20. PubMed PMID: 22583587.  161: Antonijevic D, Jevremovic D, Jovanovic S, Obradovic-Djuricic K. An in vitro radiographic analysis of the density of dental luting cements as measured by CCD-based digital radiography. Quintessence Int. 2012 May;43(5):421-8. PubMed PMID: 22536594.  162: Martínez-Rus F, Suárez MJ, Rivera B, Pradíes G. Influence of CAD/CAM systems and cement selection on marginal discrepancy of zirconia-based ceramic crowns. Am J Dent. 2012 Apr;25(2):67-72. PubMed PMID: 22779278.  163: Ilie N, Hickel R, Valceanu AS, Huth KC. Fracture toughness of dental restorative materials. Clin Oral Investig. 2012 Apr;16(2):489-98. doi: 10.1007/s00784-011-0525-z. Epub 2011 Mar 2. PubMed PMID: 21365459.  164: Sauro S, Watson TF, Thompson I, Toledano M, Nucci C, Banerjee A. Influence of air-abrasion executed with polyacrylic acid-Bioglass 45S5 on the bonding performance of a resin-modified glass ionomer cement. Eur J Oral Sci. 2012 Apr;120(2):168-77. doi: 10.1111/j.1600-0722.2012.00939.x. Epub 2012 Feb 11. PubMed PMID: 22409224.  165: Iqbal K. Particle size variations in the glass component of glass-ionomer dental cements. J Ayub Med Coll Abbottabad. 2012 Apr-Jun;24(2):41-3. PubMed PMID: 24397049.  166: Bayrak S, Sen TE, Tuloglu N. The effects of surface pretreatment on the microleakage of resin-modified glass-ionomer cement restorations. J Clin Pediatr Dent. 2012 Spring;36(3):279-84. PubMed PMID: 22838231.  167: Subbarao C, Neelakantan P, Subbarao CV. In vitro biocompatibility tests of glass ionomer cements impregnated with collagen or bioactive glass to fibroblasts. J Clin Pediatr Dent. 2012 Spring;36(3):269-74. PubMed PMID: 22838229.  168: Fragou T, Tortopidis D, Kontonasaki E, Evangelinaki E, Ioannidis K, Petridis H, Koidis P. The effect of ferrule on the fracture mode of endodontically treated canines restored with fibre posts and metal-ceramic or all-ceramic crowns. J Dent. 2012 Apr;40(4):276-85. doi: 10.1016/j.jdent.2012.01.002. Epub 2012 Jan 11.  PubMed PMID: 22265988.  169: Wang JY, Belie ND, Verstraete W. Diatomaceous earth as a protective vehicle for bacteria applied for self-healing concrete. J Ind Microbiol Biotechnol. 2012 Apr;39(4):567-77. doi: 10.1007/s10295-011-1037-1. Epub 2011 Sep 17. PubMed PMID: 21927907.  170: Bahadure RN, Pandey RK, Kumar R, Gopal K, Singh RK. An estimation of fluoride release from various dental restorative materials at different pH: In vitro study. J Indian Soc Pedod Prev Dent. 2012 Apr-Jun;30(2):122-6. doi: 10.4103/0970-4388.99983. PubMed PMID: 22918096.  171: Marković D, Petrović B, Perić T, Blagojević D. Microleakage, adaptation ability and clinical efficacy of two fluoride releasing fissure sealants. Vojnosanit Pregl. 2012 Apr;69(4):320-5. PubMed PMID: 22624423.  172: Hahnel S, Mühlbauer G, Hoffmann J, Ionescu A, Bürgers R, Rosentritt M, Handel G, Häberlein I. Streptococcus mutans and Streptococcus sobrinus biofilm formation and metabolic activity on dental materials. Acta Odontol Scand. 2012 Mar;70(2):114-21. doi: 10.3109/00016357.2011.600703. Epub 2011 Jul 25. PubMed PMID: 21780966.  173: Kohda N, Iijima M, Brantley W, Muguruma T, Yuasa T, Nakagaki S, Mizoguchi I. Effects of bonding materials on the mechanical properties of enamel around orthodontic brackets. Angle Orthod. 2012 Mar;82(2):187-95. doi: 10.2319/020411-78.1. Epub 2011 Aug 9. PubMed PMID: 21827232.  174: Wang Y, Darvell BW. Effect of elastic modulus mismatch on failure behaviour of glass ionomer cement under Hertzian indentation. Dent Mater. 2012 Mar;28(3):279-86. doi: 10.1016/j.dental.2011.11.004. Epub 2011 Nov 29. PubMed PMID: 22130463.  175: Ráth G, Kereskai L, Bauer M, Bakó P, Bányavölgyi V, Gerlinger I. Should the ossicle be denuded prior to the application of glass ionomer cement? An experimental study on rabbit. Eur Arch Otorhinolaryngol. 2012 Mar;269(3):773-80. doi: 10.1007/s00405-011-1735-3. Epub 2011 Aug 4. PubMed PMID: 21814730.  176: Guglielmi CA, Mohana A, Hesse D, Lenzi TL, Bonini GC, Raggio DP. Influence of ultrasound or halogen light on microleakage and hardness of enamel adjacent to glass ionomer cement. Int J Paediatr Dent. 2012 Mar;22(2):110-5. doi: 10.1111/j.1365-263X.2011.01173.x. Epub 2011 Aug 16. PubMed PMID: 22092553.  177: Zhou SL, Zhou J, Watanabe S, Watanabe K, Wen LY, Xuan K. In vitro study of the effects of fluoride-releasing dental materials on remineralization in an enamel erosion model. J Dent. 2012 Mar;40(3):255-63. doi: 10.1016/j.jdent.2011.12.016. Epub 2011 Dec 28. PubMed PMID: 22227269.  178: Fleming GJ, Dowling AH, Addison O. The crushing truth about glass ionomer restoratives: exposing the standard of the standard. J Dent. 2012 Mar;40(3):181-8. doi: 10.1016/j.jdent.2011.12.004. Epub 2011 Dec 9. PubMed PMID: 22178631.  179: Dowling AH, Fleming GJ, McGinley EL, Addison O. Improving the standard of the standard for glass ionomers: an alternative to the compressive fracture strength test for consideration? J Dent. 2012 Mar;40(3):189-201. doi: 10.1016/j.jdent.2011.12.002. Epub 2011 Dec 9. PubMed PMID: 22178630.  180: Goenka S, Balu R, Sampath Kumar TS. Effects of nanocrystalline calcium deficient hydroxyapatite incorporation in glass ionomer cements. J Mech Behav Biomed Mater. 2012 Mar;7:69-76. doi: 10.1016/j.jmbbm.2011.08.002. Epub 2011 Aug  16. PubMed PMID: 22340686.  181: O'Kray H, Marshall TS, Braun TM. Supplementing retention through crown/preparation modification: an in vitro study. J Prosthet Dent. 2012 Mar;107(3):186-90. doi: 10.1016/S0022-3913(12)60054-7. PubMed PMID: 22385695.  182: Navimipour EJ, Oskoee SS, Oskoee PA, Bahari M, Rikhtegaran S, Ghojazadeh M. Effect of acid and laser etching on shear bond strength of conventional and resin-modified glass-ionomer cements to composite resin. Lasers Med Sci. 2012 Mar;27(2):305-11. doi: 10.1007/s10103-010-0868-8. Epub 2011 Jan 14. PubMed PMID: 21234634.  183: Haznedaroglu E, Mentes AR, Tanboga I. In vitro evaluation of microleakage under a glass ionomer surface protector cement after different enamel treatment procedures. Oral Health Dent Manag. 2012 Mar;11(1):16-22. PubMed PMID: 22488028.  184: Monmaturapoj N, Soodsawang W, Tanodekaew S. Enhancement effect of pre-reacted glass on strength of glass-ionomer cement. Dent Mater J. 2012 Feb 3;31(1):125-30. Epub 2012 Jan 21. PubMed PMID: 22277616.  185: Pekkan G, Ozcan M. Radiopacity of different resin-based and conventional luting cements compared to human and bovine teeth. Dent Mater J. 2012 Feb 3;31(1):68-75. Epub 2012 Jan 21. PubMed PMID: 22277608.  186: de Castilho AR, Duque C, Negrini Tde C, Sacono NT, de Paula AB, Sacramento PA, de Souza Costa CA, Spolidorio DM, Puppin-Rontani RM. Mechanical and biological characterization of resin-modified glass-ionomer cement containing doxycycline hyclate. Arch Oral Biol. 2012 Feb;57(2):131-8. doi: 10.1016/j.archoralbio.2011.08.009. Epub 2011 Sep 13. PubMed PMID: 21920494.  187: Krifka S, Seidenader C, Hiller KA, Schmalz G, Schweikl H. Oxidative stress and cytotoxicity generated by dental composites in human pulp cells. Clin Oral Investig. 2012 Feb;16(1):215-24. doi: 10.1007/s00784-010-0508-5. Epub 2011 Jan 18. PubMed PMID: 21243381.  188: Nicholson JW, Czarnecka B. Maturation affects fluoride uptake by glass-ionomer dental cements. Dent Mater. 2012 Feb;28(2):e1-5. doi:  10.1016/j.dental.2011.10.011. Epub 2011 Nov 17. PubMed PMID: 22100725.  189: Al-Hiyasat AS, Al-Sa'Eed OR, Darmani H. Quality of cellular attachment to various root-end filling materials. J Appl Oral Sci. 2012 Feb;20(1):82-8. PubMed PMID: 22437683; PubMed Central PMCID: PMC3928777.  190: Tüzüner T, Ulusu T. Effect of antibacterial agents on the surface hardness of a conventional glass-ionomer cement. J Appl Oral Sci. 2012 Feb;20(1):45-9. PubMed PMID: 22437677; PubMed Central PMCID: PMC3928771.  191: Okte Z, Bayrak S, Fidanci UR, Sel T. Fluoride and aluminum release from restorative materials using ion chromatography. J Appl Oral Sci. 2012 Feb;20(1):27-31. PubMed PMID: 22437674; PubMed Central PMCID: PMC3928768.  192: Takimoto M, Ishii R, Iino M, Shimizu Y, Tsujimoto A, Takamizawa T, Ando S, Miyazaki M. Influence of temporary cement contamination on the surface free energy and dentine bond strength of self-adhesive cements. J Dent. 2012 Feb;40(2):131-8. doi: 10.1016/j.jdent.2011.11.012. Epub 2011 Nov 22. PubMed PMID: 22133591.  193: Oyagüe RC, Sánchez-Turrión A, López-Lozano JF, Suárez-García MJ. Vertical discrepancy and microleakage of laser-sintered and vacuum-cast implant-supported structures luted with different cement types. J Dent. 2012 Feb;40(2):123-30. doi: 10.1016/j.jdent.2011.11.007. Epub 2011 Nov 15. PubMed PMID: 22108101.  194: Li YQ, Wang H, Wang YJ, Chen JH. Effect of different grit sizes of diamond rotary instruments for tooth preparation on the retention and adaptation of complete coverage restorations. J Prosthet Dent. 2012 Feb;107(2):86-93. doi: 10.1016/S0022-3913(12)60029-8. PubMed PMID: 22304742.  195: Shiiya T, Mukai Y, Ten Cate JM, Teranaka T. The caries-reducing benefit of fluoride-release from dental restorative materials continues after fluoride-release has ended. Acta Odontol Scand. 2012 Jan;70(1):15-20. doi: 10.3109/00016357.2011.575078. Epub 2011 Apr 26. PubMed PMID: 21521007.  196: de Mendonça AA, de Oliveira CF, Hebling J, Costa CA. Influence of thicknesses of smear layer on the transdentinal cytotoxicity and bond strength of a resin-modified glass-ionomer cement. Braz Dent J. 2012;23(4):379-86. PubMed PMID: 23207853.  197: Dantas RV, Conde MC, Sarmento HR, Zanchi CH, Tarquinio SB, Ogliari FA, Demarco FF. Novel experimental cements for use on the dentin-pulp complex. Braz Dent J. 2012;23(4):344-50. PubMed PMID: 23207847.  198: Hazar-Yoruc B, Bavbek AB, Özcan M. The erosion kinetics of conventional and resin-modified glass-ionomer luting cements in acidic buffer solutions. Dent Mater J. 2012;31(6):1068-74. PubMed PMID: 23207217.  199: Laiteerapong A, Lochaiwatana Y, Hirata I, Okazaki M, Mori K, Murakami S, Poolthong S. A novel glass ionomer cement containing MgCO(3 )apatite induced the increased proliferation and differentiation of human pulp cells in vitro. Dent Mater J. 2012;31(5):772-8. PubMed PMID: 23037840.  200: Erdemir U, Yildiz E, Eren MM, Ozel S. Surface hardness of different restorative materials after long-term immersion in sports and energy drinks. Dent Mater J. 2012;31(5):729-36. PubMed PMID: 23037834.  201: Wang X, Chang J, Hu S. A study on the sealing ability and antibacterial activity of Ca3SiO5/CaCl2 composite cement for dental applications. Dent Mater J. 2012;31(4):617-22. PubMed PMID: 22864215.  202: Boaventura JM, Bertolini MJ, Padovani GC, de Oliveira MR, Zaghete MA, de Oliveira Júnior OB, de Andrade MF. Tissue response to experimental dental cements prepared from a modified powder glass composition. Dent Mater J. 2012;31(4):583-92. PubMed PMID: 22864211.  203: Beriat NC, Ertan AA, Yilmaz Z, Gulay G, Sahin C. Effects of different luting cements and light curing units on the sealing ability and bond strength of fiber posts. Dent Mater J. 2012;31(4):575-82. PubMed PMID: 22864210.  204: Yu H, Buchalla W, Cheng H, Wiegand A, Attin T. Topical fluoride application is able to reduce acid susceptibility of restorative materials. Dent Mater J. 2012;31(3):433-42. Epub 2012 May 14. PubMed PMID: 22673456.  205: Pereira Ade F, Poiate IA, Poiate E Jr, Rodrigues FP, Turbino ML, Miranda WG Jr. Influence of restorative techniques on marginal adaptation and dye penetration around Class V restorations. Gen Dent. 2012 Jan-Feb;60(1):e17-21. PubMed PMID: 22313988.  206: Pattanaik BK, Nagda SJ. An evaluation of retention and marginal seating of Ni-Cr alloy cast restorations using three different luting cements: an in vitro study. Indian J Dent Res. 2012 Jan-Feb;23(1):20-5. doi: 10.4103/0970-9290.99032. PubMed PMID: 22842244.  207: Cano-Batalla J, Soliva-Garriga J, Campillo-Funollet M, Munoz-Viveros CA, Giner-Tarrida L. Influence of abutment height and surface roughness on in vitro retention of three luting agents. Int J Oral Maxillofac Implants. 2012 Jan-Feb;27(1):36-41. PubMed PMID: 22299076.  208: Yamada Y, Masuda Y, Kimura Y, Hossain M, Manabe A, Hisamitsu H. Adhesiveness of various glass ionomer cements in cavities treated with Carisolv. J Clin Pediatr Dent. 2012 Winter;37(2):183-7. PubMed PMID: 23534327.  209: Shah P, Gugwad SC, Bhat C, Lodaya R. Effect of three different core materials on the fracture resistance of endodontically treated deciduous mandibular second molars: an in vitro study. J Contemp Dent Pract. 2012 Jan 1;13(1):66-70. PubMed PMID: 22430696.  210: Ahluwalia P, Chopra S, Thomas AM. Strength characteristics and marginal sealing ability of chlorhexidine-modified glass ionomer cement: an in vitro study. J Indian Soc Pedod Prev Dent. 2012 Jan-Mar;30(1):41-6. doi: 10.4103/0970-4388.95580. PubMed PMID: 22565516.  211: Veerabadhran MM, Reddy V, Nayak UA, Rao AP, Sundaram MA. The effect of retentive groove, sandblasting and cement type on the retentive strength of stainless steel crowns in primary second molars--an in vitro comparative study. J Indian Soc Pedod Prev Dent. 2012 Jan-Mar;30(1):19-26. doi: 10.4103/0970-4388.95570. PubMed PMID: 22565513.  212: Dickey BT, Tyndyk MA, Doman DA, Boyd D. In silico evaluation of stress distribution after vertebral body augmentation with conventional acrylics, composites and glass polyalkenoate cements. J Mech Behav Biomed Mater. 2012 Jan;5(1):283-90. doi: 10.1016/j.jmbbm.2011.08.007. Epub 2011 Aug 24. PubMed PMID: 22100103.  213: Geraldo-Martins VR, Lepri CP, Palma-Dibb RG. Effect of different root caries treatments on the sealing ability of conventional glass ionomer cement restorations. Lasers Med Sci. 2012 Jan;27(1):39-45. doi: 10.1007/s10103-010-0840-7. Epub 2010 Oct 1. PubMed PMID: 20886360.  214: Kanjevac T, Milovanovic M, Volarevic V, Lukic ML, Arsenijevic N, Markovic D, Zdravkovic N, Tesic Z, Lukic A. Cytotoxic effects of glass ionomer cements on human dental pulp stem cells correlate with fluoride release. Med Chem. 2012 Jan;8(1):40-5. PubMed PMID: 22420549.    215: Khoroushi M, Karvandi TM, Sadeghi R. Effect of prewarming and/or delayed light activation on resin-modified glass ionomer bond strength to tooth structures. Oper Dent. 2012 Jan-Feb;37(1):54-62. doi: 10.2341/11-137-L. Epub 2011 Sep 13. PubMed PMID: 21913858.  216: Linkevicius T, Vindasiute E, Puisys A, Linkeviciene L, Svediene O. Influence of the temperature on the cement disintegration in cement-retained implant  restorations. Stomatologija. 2012;14(4):114-7. PubMed PMID: 23455980.  217: Ngo HC, Mount G, McIntyre J, Do L. An in vitro model for the study of chemical exchange between glass ionomer restorations and partially demineralized dentin using a minimally invasive restorative technique. J Dent. 2011 Dec;39 Suppl 2:S20-6. doi: 10.1016/j.jdent.2011.10.016. Epub 2011 Nov 15. PubMed PMID: 22101125.  218: Zanata RL, Magalhães AC, Lauris JR, Atta MT, Wang L, Navarro MF. Microhardness and chemical analysis of high-viscous glass-ionomer cement after 10 years of clinical service as ART restorations. J Dent. 2011 Dec;39(12):834-40. doi: 10.1016/j.jdent.2011.09.003. Epub 2011 Sep 16. PubMed PMID: 21945449.  219: Mayanagi G, Igarashi K, Washio J, Nakajo K, Domon-Tawaraya H, Takahashi N. Evaluation of pH at the bacteria-dental cement interface. J Dent Res. 2011 Dec;90(12):1446-50. doi: 10.1177/0022034511423392. Epub 2011 Sep 20. PubMed PMID: 21933936; PubMed Central PMCID: PMC4077519.  220: Azevedo ER, Coldebella CR, Zuanon AC. Effect of ultrasonic excitation on the microtensile bond strength of glass ionomer cements to dentin after different water storage times. Ultrasound Med Biol. 2011 Dec;37(12):2133-8. doi: 10.1016/j.ultrasmedbio.2011.09.014. Epub 2011 Oct 27. PubMed PMID: 22036636.  221: Iacobescu RI, Koumpouri D, Pontikes Y, Saban R, Angelopoulos GN. Valorisation of electric arc furnace steel slag as raw material for low energy belite cements. J Hazard Mater. 2011 Nov 30;196:287-94. doi: 10.1016/j.jhazmat.2011.09.024. Epub 2011 Sep 12. PubMed PMID: 21944704.  222: Dowling AH, Fleming GJ. Can poly(acrylic) acid molecular weight mixtures improve the compressive fracture strength and elastic modulus of a glass-ionomer restorative? Dent Mater. 2011 Nov;27(11):1170-9. doi: 10.1016/j.dental.2011.08.398. Epub 2011 Sep 16. PubMed PMID: 21925723.  223: Desai PD, Das UK. Comparison of fracture resistance of teeth restored with ceramic inlay and resin composite: an in vitro study. Indian J Dent Res. 2011 Nov-Dec;22(6):877. doi: 10.4103/0970-9290.94663. PubMed PMID: 22484893.  224: Oskoee SS, Kimyai S, Bahari M, Motahari P, Eghbal MJ, Asgary S. Comparison of shear bond strength of calcium-enriched mixture cement and mineral trioxide aggregate to composite resin. J Contemp Dent Pract. 2011 Nov 1;12(6):457-62. PubMed PMID: 22269237.  225: Kim MJ, Kim YK, Kim KH, Kwon TY. Shear bond strengths of various luting cements to zirconia ceramic: surface chemical aspects. J Dent. 2011 Nov;39(11):795-803. doi: 10.1016/j.jdent.2011.08.012. Epub 2011 Sep 1. PubMed PMID: 21907260.  226: Lim BS, Lee SJ, Lim YJ, Ahn SJ. Effects of periodic fluoride treatment on fluoride ion release from fresh orthodontic adhesives. J Dent. 2011 Nov;39(11):788-94. doi: 10.1016/j.jdent.2011.08.011. Epub 2011 Aug 26. PubMed PMID: 21896303.  227: Ghoneim AG, Lutfy RA, Sabet NE, Fayyad DM. Resistance to fracture of roots obturated with novel canal-filling systems. J Endod. 2011 Nov;37(11):1590-2. doi: 10.1016/j.joen.2011.08.008. Epub 2011 Sep 28. PubMed PMID: 22000470.  228: Ebert J, Leyer A, Günther O, Lohbauer U, Petschelt A, Frankenberger R, Roggendorf MJ. Bond strength of adhesive cements to root canal dentin tested with a novel pull-out approach. J Endod. 2011 Nov;37(11):1558-61. doi: 10.1016/j.joen.2011.08.009. Epub 2011 Sep 15. PubMed PMID: 22000463.  229: Vosoughhosseini S, Lotfi M, Shahmoradi K, Saghiri MA, Zand V, Mehdipour M, Ranjkesh B, Mokhtari H, Salemmilani A, Doosti S. Microleakage comparison of glass-ionomer and white mineral trioxide aggregate used as a coronal barrier in nonvital bleaching. Med Oral Patol Oral Cir Bucal. 2011 Nov 1;16(7):e1017-21. PubMed PMID: 21743399.  230: de Paula AB, Fucio SB, Ambrosano GM, Alonso RC, Sardi JC, Puppin-Rontani RM. Biodegradation and abrasive wear of nano restorative materials. Oper Dent. 2011 Nov-Dec;36(6):670-7. doi: 10.2341/10-221-L. Epub 2011 Sep 13. PubMed PMID: 21913859.  231: Zhang Y, Burrow MF, Palamara JE, Thomas CD. Bonding to glass ionomer cements  using resin-based adhesives. Oper Dent. 2011 Nov-Dec;36(6):618-25. doi: 10.2341/10-140-L. Epub 2011 Aug 24. PubMed PMID: 21864124.  232: Seraj B, Shahrabi M, Motahari P, Ahmadi R, Ghadimi S, Mosharafian S, Mohammadi K, Javad Kharazifard M. Microleakage of stainless steel crowns placed on intact and extensively destroyed primary first molars: an in vitro study. Pediatr Dent. 2011 Nov-Dec;33(7):525-8. PubMed PMID: 22353415.  233: Memarpour M, Mesbahi M, Rezvani G, Rahimi M. Microleakage of adhesive and nonadhesive luting cements for stainless steel crowns. Pediatr Dent. 2011 Nov-Dec;33(7):501-4. PubMed PMID: 22353410.  234: A. Alsaffar; D. Tantbirojn; A. Versluis; S. Beiraghi. Protective effect of pit and fissure sealants on demineralization of adjacent enamel. Pediatr Dent. 2011 Nov-Dec;33(7):491-5. PubMed PMID: 22353408.    235: Irie M, Nagaoka N, Tamada Y, Maruo Y, Nishigawa G, Minagi S, Finger WJ. Effect of spherical silica additions on marginal gaps and compressive strength of experimental glass-ionomer cements. Am J Dent. 2011 Oct;24(5):310-4. PubMed PMID: 22165460.  236: Salas CF, Guglielmi CA, Raggio DP, Mendes FM. Mineral loss on adjacent enamel glass ionomer cements restorations after cariogenic and erosive challenges. Arch Oral Biol. 2011 Oct;56(10):1014-9. doi: 10.1016/j.archoralbio.2011.03.005. Epub 2011 Apr 13. PubMed PMID: 21489401.  237: Güncü MB, Cakan U, Canay S. Comparison of 3 luting agents on retention of implant-supported crowns on 2 different abutments. Implant Dent. 2011 Oct;20(5):349-53. doi: 10.1097/ID.0b013e318225f68e. PubMed PMID: 21811170.  238: Balkenhol M, Rupf S, Laufersweiler I, Huber K, Hannig M. Failure analysis and survival rate of post and core restorations under cyclic loading. Int Endod J. 2011 Oct;44(10):926-37. doi: 10.1111/j.1365-2591.2011.01901.x. Epub 2011 Jun 10. PubMed PMID: 21658072.  239: Wangpermtam P, Botelho MG, Dyson JE. Effect of contamination and decontamination on adhesion of a resin-modified glass-ionomer cement to bovine dentin. J Adhes Dent. 2011 Oct;13(5):445-53. doi: 10.3290/j.jad.a22095. PubMed PMID: 21935513.  240: Manso AG, González-Lopez S, Bolaños-Carmona V, Maurício PJ, Félix SA, Carvalho PA. Regional bond strength to lateral walls in class I and II ceramic inlays luted with four resin cements and glass-ionomer luting agent. J Adhes Dent. 2011 Oct;13(5):455-65. doi: 10.3290/j.jad.a19816. PubMed PMID: 21246071.  241: Dursun E, Attal JP. Combination of a self-etching adhesive and a resin-modified glass ionomer: effect of water and saliva contamination on bond strength to dentin. J Adhes Dent. 2011 Oct;13(5):439-43. doi: 10.3290/j.jad.a19652. PubMed PMID: 20978640.  242: Hamouda IM. Effects of various beverages on hardness, roughness, and solubility of esthetic restorative materials. J Esthet Restor Dent. 2011 Oct;23(5):315-22. doi: 10.1111/j.1708-8240.2011.00453.x. Epub 2011 Jun 22. PubMed PMID: 21977954.  243: Camilleri J. Scanning electron microscopic evaluation of the material interface of adjacent layers of dental materials. Dent Mater. 2011 Sep;27(9):870-8. doi: 10.1016/j.dental.2011.04.013. Epub 2011 May 11. PubMed PMID: 21565396.  244: Cassoni A, Macarini P, Leonetti ES, Shibli JA, Rodrigues JA. Halogen lamp and LED activation of resin-modified glass ionomer restorative material. In vitro microhardness after long-term storage. Eur J Paediatr Dent. 2011 Sep;12(3):150-4. PubMed PMID: 22077680.  245: Maurya R, Tripathi T, Rai P. New generation of color bonding: a comparative in vitro study. Indian J Dent Res. 2011 Sep-Oct;22(5):733-4. doi: 10.4103/0970-9290.93472. PubMed PMID: 22406729.  246: Banerjee S, Banerjee R. A comparative evaluation of the shear bond strength of five different orthodontic bonding agents polymerized using halogen and light-emitting diode curing lights: an in vitro investigation. Indian J Dent Res. 2011 Sep-Oct;22(5):731-2. doi: 10.4103/0970-9290.93469. PubMed PMID: 22406726.  247: Abesi F, Safarcherati H, Sadati J, Kheirollahi H. In vitro wear of Ionofil Molar AC quick glass-ionomer cement. Indian J Dent Res. 2011 Sep-Oct;22(5):731. doi: 10.4103/0970-9290.93468. PubMed PMID: 22406725.  248: Clarkin O, Wren A, Thornton R, Cooney J, Towler M. Antibacterial analysis of a zinc-based glass polyalkenoate cement. J Biomater Appl. 2011 Sep;26(3):277-92. doi: 10.1177/0885328210364430. Epub 2010 Jun 21. PubMed PMID: 20566659.  249: de Menezes FC, Junior GT, de Oliveira WJ, Paulino Tde P, de Moura MB, da Silva IL, de Moura MB. Analysis of the properties of dental cements after exposure to incubation media containing Streptococcus mutans. J Contemp Dent Pract. 2011 Sep 1;12(5):385-91. PubMed PMID: 22269190.  250: Bakry AS, Tamura Y, Otsuki M, Kasugai S, Ohya K, Tagami J. Cytotoxicity of 45S5 bioglass paste used for dentine hypersensitivity treatment. J Dent. 2011 Sep;39(9):599-603. doi: 10.1016/j.jdent.2011.06.003. Epub 2011 Jun 24. PubMed PMID: 21726598.    251: Elsaka SE, Hamouda IM, Swain MV. Titanium dioxide nanoparticles addition to a conventional glass-ionomer restorative: influence on physical and antibacterial properties. J Dent. 2011 Sep;39(9):589-98. doi: 10.1016/j.jdent.2011.05.006. Epub 2011 May 27. PubMed PMID: 21651955.  252: Moshaverinia A, Ansari S, Moshaverinia M, Schricker SR, Chee WW. Ultrasonically set novel NVC-containing glass-ionomer cements for applications in restorative dentistry. J Mater Sci Mater Med. 2011 Sep;22(9):2029-34. doi: 10.1007/s10856-011-4391-7. Epub 2011 Jul 19. PubMed PMID: 21769626.  253: Perdigão J, Sezinando A, Gomes G. Microtensile bond strengths and interfacial examination of a polyalkenoate-based 1-step adhesive. Am J Dent. 2011 Aug;24(4):215-20. PubMed PMID: 22016915.  254: Brauer DS, Gentleman E, Farrar DF, Stevens MM, Hill RG. Benefits and drawbacks of zinc in glass ionomer bone cements. Biomed Mater. 2011 Aug;6(4):045007. doi: 10.1088/1748-6041/6/4/045007. Epub 2011 Jun 17. PubMed PMID: 21680957.  255: Xu HH, Moreau JL, Sun L, Chow LC. Nanocomposite containing amorphous calcium  phosphate nanoparticles for caries inhibition. Dent Mater. 2011 Aug;27(8):762-9. doi: 10.1016/j.dental.2011.03.016. Epub 2011 Apr 22. PubMed PMID: 21514655; PubMed Central PMCID: PMC3125490.  256: Vanni JR, Della-Bona A, Figueiredo JA, Pedro G, Voss D, Kopper PM. Radiographic evaluation of furcal perforations sealed with different materials in dogs' teeth. J Appl Oral Sci. 2011 Aug;19(4):421-5. Epub 2011 Jul 1. PubMed PMID: 21710090.  257: Taha NA, Palamara JE, Messer HH. Fracture strength and fracture patterns of root filled teeth restored with direct resin restorations. J Dent. 2011 Aug;39(8):527-35. doi: 10.1016/j.jdent.2011.05.003. Epub 2011 May 18. PubMed PMID: 21620926.  258: Campos RE, Soares CJ, Quagliatto PS, Soares PV, de Oliveira OB Jr, Santos-Filho PC, Salazar-Marocho SM. In vitro study of fracture load and fracture pattern of ceramic crowns: a finite element and fractography analysis. J Prosthodont. 2011 Aug;20(6):447-55. doi: 10.1111/j.1532-849X.2011.00744.x. PubMed PMID: 21843228.  259: Ordinola-Zapata R, Bramante CM, Duarte MH, Ramos Fernandes LM, Camargo EJ, de Moraes IG, Bernardineli N, Vivan RR, Capelozza AL, Garcia RB. The influence of cone-beam computed tomography and periapical radiographic evaluation on the assessment of periapical bone destruction in dog's teeth. Oral Surg Oral Med Oral Pathol Oral Radiol Endod. 2011 Aug;112(2):272-9. doi: 10.1016/j.tripleo.2011.01.031. Epub 2011 May 6. PubMed PMID: 21530334.  260: Fourie J, Smit CF. Cervical microleakage in Class II open-sandwich restorations: an in vitro study. SADJ. 2011 Aug;66(7):320-4. PubMed PMID: 23198465.  261: Costa CA, Ribeiro AP, Giro EM, Randall RC, Hebling J. Pulp response after application of two resin modified glass ionomer cements (RMGICs) in deep cavities of prepared human teeth. Dent Mater. 2011 Jul;27(7):e158-70. doi:  10.1016/j.dental.2011.04.002. Epub 2011 May 5. PubMed PMID: 21549419.  262: Rao A, Rao A, Sudha P. Fluoride rechargability of a non-resin auto-cured glass ionomer cement from a fluoridated dentifrice: an in vitro study. J Indian Soc Pedod Prev Dent. 2011 Jul-Sep;29(3):202-4. doi: 10.4103/0970-4388.85812. PubMed PMID: 21985875.  263: Kimyai S, Lotfipour F, Pourabbas R, Sadr A, Nikazar S, Milani M. Effect of two prophylaxis methods on adherence of Streptococcus mutans to microfilled composite resin and giomer surfaces. Med Oral Patol Oral Cir Bucal. 2011 Jul 1;16(4):e561-7. PubMed PMID: 20711117.  264: Nakamura K, Deyama Y, Yoshimura Y, Hashimoto M, Kaga M, Suzuki K, Yawaka Y. Tannin-fluoride preparation attenuates prostaglandin E2 production by dental pulp cells. Mol Med Rep. 2011 Jul-Aug;4(4):641-4. doi: 10.3892/mmr.2011.476. Epub 2011 Apr 15. PubMed PMID: 21503579.  265: Sampaio PC, de Almeida Júnior AA, Francisconi LF, Casas-Apayco LC, Pereira JC, Wang L, Atta MT. Effect of conventional and resin-modified glass-ionomer liner on dentin adhesive interface of Class I cavity walls after thermocycling. Oper Dent. 2011 Jul-Aug;36(4):403-12. doi: 10.2341/09-240-L. PubMed PMID: 21913841.  266: Briso AL, Caruzo LP, Guedes AP, Catelan A, dos Santos PH. In vitro evaluation of surface roughness and microhardness of restorative materials submitted to erosive challenges. Oper Dent. 2011 Jul-Aug;36(4):397-402. doi: 10.2341/10-356-L. Epub 2011 Aug 9. PubMed PMID: 21827225.  267: El-Askary F, Nassif M. Bonding nano-filled resin-modified glass ionomer to dentin using different self-etch adhesives. Oper Dent. 2011 Jul-Aug;36(4):413-21. doi: 10.2341/10-383-L. Epub 2011 Aug 5. PubMed PMID: 21819202.  268: Al Hamad KQ, Al Rashdan BA, Abu-Sitta EH. The effects of height and surface roughness of abutments and the type of cement on bond strength of cement-retained implant restorations. Clin Oral Implants Res. 2011 Jun;22(6):638-44. doi: 10.1111/j.1600-0501.2010.02011.x. Epub 2010 Oct 6. PubMed PMID: 21039897.  269: Dowling AH, Fleming GJ. The influence of poly(acrylic) acid number average molecular weight and concentration in solution on the compressive fracture strength and modulus of a glass-ionomer restorative. Dent Mater. 2011 Jun;27(6):535-43. doi: 10.1016/j.dental.2011.02.003. Epub 2011 Mar 5. PubMed PMID: 21377721.  270: Kawai N, Shinya A, Yokoyama D, Gomi H, Shinya A. Effect of cyclic impact load on shear bond strength of zirconium dioxide ceramics. J Adhes Dent. 2011 Jun;13(3):267-77. doi: 10.3290/j.jad.a19471. PubMed PMID: 20978648.  271: Barutcigil C, Harorli OT, Yildiz M, Ozcan E, Arslan H, Bayindir F. The color differences of direct esthetic restorative materials after setting and compared with a shade guide. J Am Dent Assoc. 2011 Jun;142(6):658-65. PubMed PMID:  21628688.  272: Parirokh M, Asgary S, Eghbal MJ, Kakoei S, Samiee M. A comparative study of using a combination of calcium chloride and mineral trioxide aggregate as the pulp-capping agent on dogs' teeth. J Endod. 2011 Jun;37(6):786-8. doi:  10.1016/j.joen.2011.03.010. PubMed PMID: 21787489.  273: Ugarte Núñez D, Terossi De Godoi A, De Biagi Freitas D, Catirse AB. In vitro study on micro-hardness of resin-modified glass ionomers at different depths. Minerva Stomatol. 2011 Jun;60(6):297-302. PubMed PMID: 21666565.  274: Chen YL, Ko MS, Lai YC, Chang JE. Hydration and leaching characteristics of cement pastes made from electroplating sludge. Waste Manag. 2011 Jun;31(6):1357-63. doi: 10.1016/j.wasman.2010.12.018. Epub 2011 Feb 3. PubMed PMID: 21295462.  275: Yang B, Flaim G, Dickens SH. Remineralization of human natural caries and artificial caries-like lesions with an experimental whisker-reinforced ART composite. Acta Biomater. 2011 May;7(5):2303-9. doi: 10.1016/j.actbio.2011.01.002. Epub 2011 Jan 11. PubMed PMID: 21232637; PubMed Central PMCID: PMC3074036.  276: Farret MM, de Lima EM, Mota EG, Oshima HM, Barth V, de Oliveira SD. Can we add chlorhexidine into glass ionomer cements for band cementation? Angle Orthod. 2011 May;81(3):496-502. doi: 10.2319/090310-518.1. Epub 2011 Feb 7. PubMed PMID: 21299380.  277: Xie D, Weng Y, Guo X, Zhao J, Gregory RL, Zheng C. Preparation and evaluation of a novel glass-ionomer cement with antibacterial functions. Dent Mater. 2011 May;27(5):487-96. doi: 10.1016/j.dental.2011.02.006. PubMed PMID: 21388668.  278: Zhao J, Xie D. A novel hyperbranched poly(acrylic acid) for improved resin-modified glass-ionomer restoratives. Dent Mater. 2011 May;27(5):478-86. doi: 10.1016/j.dental.2011.02.005. Epub 2011 Mar 5. PubMed PMID: 21377199.  279: Mazaheri H, Khoroushi M, Shafiei E, Ghorbanipour R, Majdzade F. Bond strength of composite-resin and resin-modified glass ionomer to bleached enamel: delay bonding versus an antioxidant agent. Indian J Dent Res. 2011 May-Jun;22(3):432-5. doi: 10.4103/0970-9290.87066. PubMed PMID: 22048584.  280: van de Sande FH, Silva AF, Michelon D, Piva E, Cenci MS, Demarco FF. Surface roughness of orthodontic band cements with different compositions. J Appl Oral Sci. 2011 May-Jun;19(3):223-7. PubMed PMID: 21625737.  281: Gupta I, Gupta S, Kothari A. Revisiting amalgam: a comparative study between bonded amalgam restoration and amalgam retained with undercuts. J Contemp Dent Pract. 2011 May 1;12(3):164-70. PubMed PMID: 22186810.  282: Al-Hezaimi K, Al-Tayar BA, Bajuaifer YS, Salameh Z, Al-Fouzan K, Tay FR. A hybrid approach to direct pulp capping by using emdogain with a capping material. J Endod. 2011 May;37(5):667-72. doi: 10.1016/j.joen.2011.02.003. PubMed PMID:  21496668.  283: Moshaverinia A, Ansari S, Roohpour N, Reshad M, Schricker SR, Chee WW. Effects of N-vinylcaprolactam containing polyelectrolytes on hardness, fluoride release and water sorption of conventional glass ionomers. J Prosthet Dent. 2011 May;105(5):323-31. doi: 10.1016/S0022-3913(11)60062-0. PubMed PMID: 21530758.  284: Carvalho TS, van Amerongen WE, de Gee A, Bönecker M, Sampaio FC. Shear bond strengths of three glass ionomer cements to enamel and dentine. Med Oral Patol Oral Cir Bucal. 2011 May 1;16(3):e406-10. PubMed PMID: 20526266.  285: Peutzfeldt A, Sahafi A, Flury S. Bonding of restorative materials to dentin with various luting agents. Oper Dent. 2011 May-Jun;36(3):266-73. doi:  10.2341/10-236-L. Epub 2011 Jul 8. PubMed PMID: 21740244.  286: Hooshmand T, Mohajerfar M, Keshvad A, Motahhary P. Microleakage and marginal gap of adhesive cements for noble alloy full cast crowns. Oper Dent. 2011 May-Jun;36(3):258-65. doi: 10.2341/10-253-L. Epub 2011 Jul 8. PubMed PMID: 21740242.  287: Peng Y, Stark PC, Rich A Jr, Loo CY. Marginal microleakage of triage sealant under different moisture contamination. Pediatr Dent. 2011 May-Jun;33(3):203-6. PubMed PMID: 21703071.  288: Lohbauer U, Krämer N, Siedschlag G, Schubert EW, Lauerer B, Müller FA, Petschelt A, Ebert J. Strength and wear resistance of a dental glass-ionomer cement with a novel nanofilled resin coating. Am J Dent. 2011 Apr;24(2):124-8. PubMed PMID: 21698994.  289: Boushell LW, Getz G, Swift EJ Jr, Walter R. Bond strengths of a silorane composite to various substrates. Am J Dent. 2011 Apr;24(2):93-6. PubMed PMID: 21698988.  290: Hammad HM, Hamadah MA, Al-Omari WM. Histological evaluation of rat tissue response to GMTA, Retroplast, and Geristore retrograde filling materials. Aust Endod J. 2011 Apr;37(1):18-25. doi: 10.1111/j.1747-4477.2009.00195.x. PubMed PMID: 21418410.  291: Deb S, Di Silvio L, Mackler HE, Millar BJ. Pre-warming of dental composites. Dent Mater. 2011 Apr;27(4):e51-9. doi: 10.1016/j.dental.2010.11.009. Epub 2010 Dec 9. PubMed PMID: 21145580.  292: Abduo J, Swain M. Self-reparability of glass-ionomer cements: an in vitro investigation. Eur J Oral Sci. 2011 Apr;119(2):187-91. doi: 10.1111/j.1600-0722.2011.00810.x. Epub 2011 Feb 22. PubMed PMID: 21410561.  293: Andersson J, Dahlgren U. Effects on mouse immunity of long-term exposure in vivo to minute amounts of HEMA. Eur J Oral Sci. 2011 Apr;119(2):109-14. doi: 10.1111/j.1600-0722.2011.00818.x. PubMed PMID: 21410549.  294: Cheng HY, Chen CH, Li CL, Tsai HH, Chou TH, Wang WN. Bond strength of orthodontic light-cured resin-modified glass ionomer cement. Eur J Orthod. 2011 Apr;33(2):180-4. doi: 10.1093/ejo/cjq056. Epub 2010 Aug 30. PubMed PMID: 20805142.  295: Markovic D, Petrovic B, Peric T, Miletic I, Andjelkovic S. The impact of fissure depth and enamel conditioning protocols on glass-ionomer and resin-based fissure sealant penetration. J Adhes Dent. 2011 Apr;13(2):171-8. doi: 10.3290/j.jad.a19006. PubMed PMID: 21594230.  296: Troca VB, Fernandes KB, Terrile AE, Marcucci MC, Andrade FB, Wang L. Effect of green propolis addition to physical mechanical properties of glass ionomer cements. J Appl Oral Sci. 2011 Apr;19(2):100-5. PubMed PMID: 21552709.  297: Cannon M, Wagner C, Thobaben JZ, Jurado R, Solt D. Early response of mechanically exposed dental pulps of swine to antibacterial-hemostatic agents or diode laser irradiation. J Clin Pediatr Dent. 2011 Spring;35(3):271-6. PubMed PMID: 21678669.  298: Dittmer MP, Kohorst P, Borchers L, Schwestka-Polly R, Stiesch M. Stress analysis of an all-ceramic FDP loaded according to different occlusal concepts. J Oral Rehabil. 2011 Apr;38(4):278-85. doi: 10.1111/j.1365-2842.2010.02147.x. Epub 2010 Aug 31. PubMed PMID: 20819134.  299: Ayad MF, Bahannan SA, Rosenstiel SF. Influence of irrigant, dowel type, and root-reinforcing material on fracture resistance of thin-walled endodontically treated teeth. J Prosthodont. 2011 Apr;20(3):180-9. doi: 10.1111/j.1532-849X.2011.00687.x. Epub 2011 Feb 15. PubMed PMID: 21323785.  300: Lodiene G, Kleivmyr M, Bruzell E, Ørstavik D. Sealing ability of mineral trioxide aggregate, glass ionomer cement and composite resin when repairing large furcal perforations. Br Dent J. 2011 Mar 12;210(5):E7. doi: 10.1038/sj.bdj.2011.198. PubMed PMID: 21394123.  301: Al Zraikat H, Palamara JE, Messer HH, Burrow MF, Reynolds EC. The incorporation of casein phosphopeptide-amorphous calcium phosphate into a glass ionomer cement. Dent Mater. 2011 Mar;27(3):235-43. doi: 10.1016/j.dental.2010.10.008. Epub 2010 Nov 18. PubMed PMID: 21087789.  302: Borges MA, Matos IC, Mendes LC, Gomes AS, Miranda MS. Degradation of polymeric restorative materials subjected to a high caries challenge. Dent Mater. 2011 Mar;27(3):244-52. doi: 10.1016/j.dental.2010.10.009. Epub 2010 Nov 13. PubMed PMID: 21075443.  303: Yilmaz Y, Kara NB, Yilmaz A, Sahin H. Wear and repair of stainless steel crowns. Eur J Paediatr Dent. 2011 Mar;12(1):25-30. PubMed PMID: 21434732.  304: Melara Munguía A, Arregui Gambús M, Guinot Jimeno F, Bellet Dalmau LJ. Temperature changes caused by light curing units on dentine of primary teeth. Eur J Paediatr Dent. 2011 Mar;12(1):7-12. PubMed PMID: 21434728.  305: Hengtrakool C, Kukiattrakoon B, Kedjarune-Leggat U. Gradual surface degradation of restorative materials by acidic agents. Gen Dent. 2011 Mar-Apr;59(2):e50-62. PubMed PMID: 21903509.  306: Vasudeva G, Bogra P, Nikhil V, Singh V. Effect of occlusal restoration on stresses around class V restoration interface: a finite-element study. Indian J Dent Res. 2011 Mar-Apr;22(2):295-302. doi: 10.4103/0970-9290.84308. PubMed PMID: 21891903.  307: Parolia A, Kundabala M, Gupta V, Verma M, Batra C, Shenoy R, Srikant N. Microleakage of bonded amalgam restorations using different adhesive agents with dye under vacuum: an in vitro study. Indian J Dent Res. 2011 Mar-Apr;22(2):252-5. doi: 10.4103/0970-9290.84298. PubMed PMID: 21891895.  308: Singla A, Garg S, Jindal SK, Suma Sogi HP, Sharma D. In vitro evaluation of marginal leakage using invasive and noninvasive technique of light cure glass ionomer and flowable polyacid modified composite resin used as pit and fissure sealant. Indian J Dent Res. 2011 Mar-Apr;22(2):205-9. doi: 10.4103/0970-9290.84286. PubMed PMID: 21891886.  309: Zavanelli AC, Mazaro VQ, Silva CR, Zavanelli RA, Mancuso DN. Surface roughness analysis of four restorative materials exposed to 10% and 15% carbamide peroxide. Int J Prosthodont. 2011 Mar-Apr;24(2):155-7. PubMed PMID: 21479284.  310: Ahn SJ, Lee SJ, Lee DY, Lim BS. Effects of different fluoride recharging protocols on fluoride ion release from various orthodontic adhesives. J Dent. 2011 Mar;39(3):196-201. doi: 10.1016/j.jdent.2010.12.003. Epub 2010 Dec 13. PubMed PMID: 21147194.  311: Rakkiettiwong N, Hengtrakool C, Thammasitboon K, Kedjarune-Leggat U. Effect  of novel chitosan-fluoroaluminosilicate glass ionomer cement with added transforming growth factor beta-1 on pulp cells. J Endod. 2011 Mar;37(3):367-71. doi: 10.1016/j.joen.2010.11.031. Epub 2011 Jan 15. PubMed PMID: 21329823.  312: Moshaverinia A, Chee WW, Brantley WA, Schricker SR. Surface properties and bond strength measurements of N-vinylcaprolactam (NVC)-containing glass-ionomer cements. J Prosthet Dent. 2011 Mar;105(3):185-93. doi: 10.1016/S0022-3913(11)60027-9. PubMed PMID: 21356411.  313: Kasraei S, Azarsina M, Majidi S. In vitro comparison of microleakage of posterior resin composites with and without liner using two-step etch-and-rinse and self-etch dentin adhesive systems. Oper Dent. 2011 Mar-Apr;36(2):213-21. doi: 10.2341/10-215-L. Epub 2011 Jun 24. PubMed PMID: 21702678.  314: Wang Y, Liao Z, Liu D, Liu Z, McIntyre GT, Jian F, Lv W, Yang J, Zhao Z, Lai W. 3D-fEA of stress levels and distributions for different bases under a Class I composite restoration. Am J Dent. 2011 Feb;24(1):3-7. PubMed PMID: 21469399.  315: Versluis A, Tantbirojn D, Lee MS, Tu LS, DeLong R. Can hygroscopic expansion compensate polymerization shrinkage? Part I. Deformation of restored teeth. Dent Mater. 2011 Feb;27(2):126-33. doi: 10.1016/j.dental.2010.09.007. Epub 2010 Oct 20. PubMed PMID: 20970176.  316: Chuang SF, Chang CH, Chen TY. Spatially resolved assessments of composite shrinkage in MOD restorations using a digital-image-correlation technique. Dent Mater. 2011 Feb;27(2):134-43. doi: 10.1016/j.dental.2010.09.008. Epub 2010 Oct  15. PubMed PMID: 20951420.  317: Heintze SD, Barkmeier WW, Latta MA, Rousson V. Round robin test: wear of nine dental restorative materials in six different wear simulators – supplement to the round robin test of 2005. Dent Mater. 2011 Feb;27(2):e1-9. doi:  10.1016/j.dental.2010.09.003. PubMed PMID: 20888629.  318: El-Tatari A, de Soet JJ, de Gee AJ, Abou Shelib M, van Amerongen WE. Influence of Salvadora persica (miswak) extract on physical and antimicrobial properties of glass ionomer cement. Eur Arch Paediatr Dent. 2011 Feb;12(1):22-5. PubMed PMID: 21299941.  319: Marquezan M, Skupien JA, da Silveira BL, Ciamponi A. Nanoleakage related to bond strength in RM-GIC and adhesive restorations. Eur Arch Paediatr Dent. 2011 Feb;12(1):15-21. PubMed PMID: 21299940.  320: Modareszadeh MR, Chogle SA, Mickel AK, Jin G, Kowsar H, Salamat N, Shaikh S, Qutbudin S. Cytotoxicity of set polymer nanocomposite resin root-end filling materials. Int Endod J. 2011 Feb;44(2):154-61. doi: 10.1111/j.1365-2591.2010.01825.x. Epub 2010 Nov 17. PubMed PMID: 21083578.  321: Tüzüner T, Kuşgöz A, Er K, Taşdemir T, Buruk K, Kemer B. Antibacterial activity and physical properties of conventional glass-ionomer cements containing chlorhexidine diacetate/cetrimide mixtures. J Esthet Restor Dent. 2011 Feb;23(1):46-55. doi: 10.1111/j.1708-8240.2010.00385.x. PubMed PMID: 21323839.  322: Shahid S, Billington RW, Hill RG. The effect of ultrasound on the uptake of fluoride by glass ionomer cements. J Mater Sci Mater Med. 2011 Feb;22(2):247-51. doi: 10.1007/s10856-010-4209-z. Epub 2011 Jan 8. PubMed PMID: 21221738.  323: Yu H, Li Q, Cheng H, Wang Y. The effects of temperature and bleaching gels on the properties of tooth-colored restorative materials. J Prosthet Dent. 2011 Feb;105(2):100-7. doi: 10.1016/S0022-3913(11)60007-3. PubMed PMID: 21262407.  324: Zahra VN, Kohen SG, Macchi RL. Powder-liquid ratio and properties of two restorative glass ionomer cements. Acta Odontol Latinoam. 2011;24(2):200-4. PubMed PMID: 22165320.  325: Reis KR, Spyrides GM, Oliveira JA, Jnoub AA, Dias KR, Bonfantes G. Effect of cement type and water storage time on the push-out bond strength of a glass fiber post. Braz Dent J. 2011;22(5):359-64. PubMed PMID: 22011889.  326: Basso GR, Della Bona A, Gobbi DL, Cecchetti D. Fluoride release from restorative materials. Braz Dent J. 2011;22(5):355-8. PubMed PMID: 22011888.  327: Paschoal MA, Gurgel CV, Rios D, Magalhães AC, Buzalaf MA, Machado MA. Fluoride release profile of a nanofilled resin-modified glass ionomer cement. Braz Dent J. 2011;22(4):275-9. PubMed PMID: 21861024.  328: Arita K, Yamamoto A, Shinonaga Y, Harada K, Abe Y, Nakagawa K, Sugiyama S.  Hydroxyapatite particle characteristics influence the enhancement of the mechanical and chemical properties of conventional restorative glass ionomer cement. Dent Mater J. 2011;30(5):672-83. Epub 2011 Sep 23. PubMed PMID: 21946488.  329: Muraguchi K, Minami H, Minesaki Y, Suzuki S, Tanaka T. A study of self-adhesive resin cements for bonding to silver-palladium-copper-gold alloy -- effect of including primer components in cement base. Dent Mater J. 2011;30(2):199-205. Epub 2011 Mar 12. PubMed PMID: 21422670.  330: Kurata S, Umemoto K. Characteristics of acrylic acid-modified temperature-responsive polymers and application as cement liquid. Dent Mater J. 2011;30(1):115-20. Epub 2011 Jan 26. PubMed PMID: 21282878.  331: Beattie S, Taskonak B, Jones J, Chin J, Sanders B, Tomlin A, Weddell J. Fracture resistance of 3 types of primary esthetic stainless steel crowns. J Can Dent Assoc. 2011;77:b90. PubMed PMID: 21736864.  332: Mousavinasab SM, Khoroushi M, Keshani F, Hashemi S. Flexural strength and morphological characteristics of resin-modified glass-ionomer containing bioactive glass. J Contemp Dent Pract. 2011 Jan 1;12(1):41-6. PubMed PMID: 22186689.  333: Dhull KS, Nandlal B. Effect of low-concentration daily topical fluoride application on fluoride release of giomer and compomer: an in vitro study. J Indian Soc Pedod Prev Dent. 2011 Jan-Mar;29(1):39-45. doi: 10.4103/0970-4388.79930. PubMed PMID: 21521917.  334: Upadhyay S, Rao A. Nanoionomer: evaluation of microleakage. J Indian Soc Pedod Prev Dent. 2011 Jan-Mar;29(1):20-4. doi: 10.4103/0970-4388.79919. PubMed PMID: 21521913.  335: Manuja N, Pandit IK, Srivastava N, Gugnani N, Nagpal R. Comparative evaluation of shear bond strength of various esthetic restorative materials to dentin: an in vitro study. J Indian Soc Pedod Prev Dent. 2011 Jan-Mar;29(1):7-13. doi: 10.4103/0970-4388.79913. PubMed PMID: 21521911.  336: Wren AW, Cummins NM, Laffir FR, Hudson SP, Towler MR. The bioactivity and ion release of titanium-containing glass polyalkenoate cements for medical applications. J Mater Sci Mater Med. 2011 Jan;22(1):19-28. doi: 10.1007/s10856-010-4184-4. Epub 2010 Nov 13. PubMed PMID: 21076857.  337: Coldebella CR, Santos-Pinto L, Zuanon AC. Effect of ultrasonic excitation on the porosity of glass ionomer cement: a scanning electron microscope evaluation. Microsc Res Tech. 2011 Jan;74(1):54-7. doi: 10.1002/jemt.20873. PubMed PMID: 21181710.  338: Wadenya R, Menon S, Mante F. Effect of chlorhexidine disinfectant on bond strength of glass ionomer cement to dentin using atraumatic restorative treatment. N Y State Dent J. 2011 Jan;77(1):23-6. PubMed PMID: 21417162.  339: Neelakantan P, John S, Anand S, Sureshbabu N, Subbarao C. Fluoride release from a new glass-ionomer cement. Oper Dent. 2011 Jan-Feb;36(1):80-5. doi: 10.2341/10-219-LR. Epub 2011 Feb 21. PubMed PMID: 21488733.  340: Abd El Halim S, Zaki D. Comparative evaluation of microleakage among three different glass ionomer types. Oper Dent. 2011 Jan-Feb;36(1):36-42. doi: 10.2341/10-123-LR. Epub 2011 Mar 24. PubMed PMID: 21488727.    341: Pithon MM, dos Santos RL, de Oliveira Ruellas AC, Nojima LI, Sant'anna EF. In vitro evaluation of fluoride release of orthodontic bonding adhesives. Orthodontics (Chic.). 2011 Winter;12(4):290-5. PubMed PMID: 22299103.  342: Peutzfeldt A, Sahafi A, Flury S. [Dentin bonding of cements. The bonding of cements with dentin in combination with various indirect restorative materials]. Schweiz Monatsschr Zahnmed. 2011;121(12):1153-63. French, German. PubMed PMID: 22203577.  343: McKissock AJ, Mines P, Sweet MB, Klyn SL. Ten-month in vitro leakage study of a single-cone obturation system. US Army Med Dep J. 2011 Jan-Mar:42-7. PubMed PMID: 21409760.  344: Moshaverinia A, Brantley WA, Chee WW, Rohpour N, Ansari S, Zheng F, Heshmati RH, Darr JA, Schricker SR, Rehman IU. Measure of microhardness, fracture toughness and flexural strength of N-vinylcaprolactam (NVC)-containing glass-ionomer dental cements. Dent Mater. 2010 Dec;26(12):1137-43. doi: 10.1016/j.dental.2010.08.002. Epub 2010 Sep 20. PubMed PMID: 20851458.  345: Villat C, Tran XV, Pradelle-Plasse N, Ponthiaux P, Wenger F, Grosgogeat B, Colon P. Impedance methodology: A new way to characterize the setting reaction of dental cements. Dent Mater. 2010 Dec;26(12):1127-32. doi: 10.1016/j.dental.2010.07.013. Epub 2010 Aug 21. Erratum in: Dent Mater. 2011 May;27(5):507. Tran, V X [corrected to Tran, Xuan-Vinh]. PubMed PMID: 20728209.  346: Heintze SD, Cavalleri A. Retention loss of class v restorations after artificial aging. J Adhes Dent. 2010 Dec;12(6):443-9. doi: 10.3290/j.jad.a18240. PubMed PMID: 21246065.  347: Can-Karabulut DC, Akıncıoglu A, Ozyegin SL, Karabulut B. Influence of experimental provisional cements containing zeolite, bone hydroxyapatite and linoleic Acid on bond strength of composite to dentin in vitro. J Adhes Dent. 2010 Dec;12(6):469-75. doi: 10.3290/j.jad.a17727. PubMed PMID: 20157651.  348: Fonseca RB, Branco CA, Quagliatto PS, Gonçalves Lde S, Soares CJ, Carlo HL, Correr-Sobrinho L. Influence of powder/liquid ratio on the radiodensity and diametral tensile strength of glass ionomer cements. J Appl Oral Sci. 2010  Dec;18(6):577-84. PubMed PMID: 21308288; PubMed Central PMCID: PMC3881762.  349: Kohorst P, Butzheinen LO, Dittmer MP, Heuer W, Borchers L, Stiesch M. Influence of preliminary damage on the load-bearing capacity of zirconia fixed dental prostheses. J Prosthodont. 2010 Dec;19(8):606-13. doi: 10.1111/j.1532-849X.2010.00640.x. Epub 2010 Aug 16. PubMed PMID: 20723012.  350: Knösel M, Mattysek S, Jung K, Sadat-Khonsari R, Kubein-Meesenburg D, Bauss O, Ziebolz D. Impulse debracketing compared to conventional debonding. Angle Orthod. 2010 Nov;80(6):1036-44. doi: 10.2319/033110-48.1. PubMed PMID: 20677952.  351: Dastjerdie EV, Zarnegar H, Behnaz M, Seifi M. Strength of attachment between band and glass ionomer cement. Aust Orthod J. 2010 Nov;26(2):149-52. PubMed PMID: 21175024.  352: Moreau JL, Xu HH. Fluoride releasing restorative materials: Effects of pH on mechanical properties and ion release. Dent Mater. 2010 Nov;26(11):e227-35. doi: 10.1016/j.dental.2010.07.004. Epub 2010 Aug 12. PubMed PMID: 20708253; PubMed Central PMCID: PMC2948642.  353: Santosa RE, Martin W, Morton D. Effects of a cementing technique in addition to luting agent on the uniaxial retention force of a single-tooth implant-supported restoration: an in vitro study. Int J Oral Maxillofac Implants. 2010 Nov-Dec;25(6):1145-52. PubMed PMID: 21197491.  354: Cardoso MV, Delmé KI, Mine A, Neves Ade A, Coutinho E, De Moor RJ, Van Meerbeek B. Towards a better understanding of the adhesion mechanism of resin-modified glass-ionomers by bonding to differently prepared dentin. J Dent. 2010 Nov;38(11):921-9. doi: 10.1016/j.jdent.2010.08.009. Epub 2010 Aug 20. PubMed  PMID: 20728505.  355: Kim NR, Park HC, Kim I, Lim BS, Yang HC. In vitro cytocompatibility of N-acetylcysteine-supplemented dentin bonding agents. J Endod. 2010 Nov;36(11):1844-50. doi: 10.1016/j.joen.2010.08.005. Epub 2010 Sep 16. PubMed PMID: 20951298.  356: Korkmaz Y, Ozel E, Attar N, Ozge Bicer C. Influence of different conditioning methods on the shear bond strength of novel light-curing  nano-ionomer restorative to enamel and dentin. Lasers Med Sci. 2010 Nov;25(6):861-6. doi: 10.1007/s10103-009-0718-8. Epub 2009 Aug 18. PubMed PMID: 19688586.  357: Yu H, Li Q, Attin T, Wang Y. Protective effect of resin coating on the microleakage of Class V restorations following treatment with carbamide peroxide in vitro. Oper Dent. 2010 Nov-Dec;35(6):634-40. doi: 10.2341/10-039-LR. PubMed PMID: 21180002.  358: Wang L, Honorio HM, Rios D, Delbem AC, Palma-Dibb RG, Buzalaf MA, Atta MT, Tenuta LM. Short-term in situ/ex vivo study of the anticariogenic potential of a resin-modified glass-ionomer cement associated with adhesive systems. Quintessence Int. 2010 Nov-Dec;41(10):e192-9. PubMed PMID: 20927415.  359: Osorio E, Osorio R, Toledano M, Quevedo-Sarmiento J, Ruiz-Bravo A. Influence  of different resin-based restorative materials on mutans streptococci adhesion. An in vitro study. Am J Dent. 2010 Oct;23(5):275-8. PubMed PMID: 21207795.  360: Minamikawa H, Yamada M, Iwasa F, Ueno T, Deyama Y, Suzuki K, Yawaka Y, Ogawa T. Amino acid derivative-mediated detoxification and functionalization of dual cure dental restorative material for dental pulp cell mineralization. Biomaterials. 2010 Oct;31(28):7213-25. doi: 10.1016/j.biomaterials.2010.06.018. PubMed PMID: 20621351.  361: Li J, Naito Y, Chen JR, Goto T, Ishida Y, Kawano T, Tomotake Y, Ichikawa T. New glass polyalkenoate temporary cement for cement-retained implant restoration: evaluation of elevation and retentive strength. Dent Mater J. 2010 Oct;29(5):589-95. Epub 2010 Sep 18. PubMed PMID: 20877129.  362: Raggio DP, Sônego FG, Camargo LB, Marquezan M, Imparato JC. Efficiency of different polyacrylic acid concentrations on the smear layer, after ART technique, by Scanning Electron Microscopy (SEM). Eur Arch Paediatr Dent. 2010 Oct;11(5):232-5. PubMed PMID: 20932397.  363: Weng Y, Guo X, Gregory R, Xie D. A novel antibacterial dental glass-ionomer cement. Eur J Oral Sci. 2010 Oct;118(5):531-4. PubMed PMID: 20853548.  364: Endo K, Hashimoto M, Haraguchi K, Ohno H. Crystal growth by restorative filling materials. Eur J Oral Sci. 2010 Oct;118(5):489-93. doi: 10.1111/j.1600-0722.2010.00764.x. PubMed PMID: 20831583.  365: Deepalakshmi M, Poorni S, Miglani R, Rajamani I, Ramachandran S. Evaluation of the antibacterial and physical properties of glass ionomer cements containing chlorhexidine and cetrimide: an in-vitro study. Indian J Dent Res. 2010 Oct-Dec;21(4):552-6. doi: 10.4103/0970-9290.74217. PubMed PMID: 21187624.  366: Sonmez NS, Sonmez E, Akcaboy C. Evaluation of biocompatibility of Targis Dentin and Artglass by using subcutaneous implantation test. Indian J Dent Res. 2010 Oct-Dec;21(4):537-43. doi: 10.4103/0970-9290.74211. PubMed PMID: 21187621.  367: Perillo L, Sorrentino R, Apicella D, Quaranta A, Gherlone E, Zarone F, Ferrari M, Aversa R, Apicella A. Nonlinear visco-elastic finite element analysis of porcelain veneers: a submodelling approach to strain and stress distributions in adhesive and resin cement. J Adhes Dent. 2010 Oct;12(5):403-13. doi: 10.3290/j.jad.a18394. PubMed PMID: 20157681.  368: Bagheri R, Mese A, Burrow MF, Tyas MJ. Comparison of the effect of storage media on shear punch strength of resin luting cements. J Dent. 2010 Oct;38(10):820-7. doi: 10.1016/j.jdent.2010.06.014. Epub 2010 Jul 1. PubMed PMID: 20600555.  369: Raghunath Reddy MH, Subba Reddy VV, Basappa N. A comparative study of retentive strengths of zinc phosphate, polycarboxylate and glass ionomer cements with stainless steel crowns - an in vitro study. J Indian Soc Pedod Prev Dent. 2010 Oct-Dec;28(4):245-50. doi: 10.4103/0970-4388.76150. PubMed PMID: 21273711.  370: Pavan S, dos Santos PH, Berger S, Bedran-Russo AK. The effect of dentin pretreatment on the microtensile bond strength of self-adhesive resin cements. J Prosthet Dent. 2010 Oct;104(4):258-64. doi: 10.1016/S0022-3913(10)60134-5. PubMed PMID: 20875530.  371: Kacimi L, Cyr M, Clastres P. Synthesis of alpha'L-C2S cement from fly-ash using the hydrothermal method at low temperature and atmospheric pressure. J Hazard Mater. 2010 Sep 15;181(1-3):593-601. doi: 10.1016/j.jhazmat.2010.05.054. Epub 2010 May 20. PubMed PMID: 20541318.  372: Malkoc S, Corekci B, Botsali HE, Yalçin M, Sengun A. Cytotoxic effects of resin-modified orthodontic band adhesives. Are they safe? Angle Orthod. 2010 Sep;80(5):890-5. doi: 10.2319/101509-577.1. PubMed PMID: 20578860.  373: Cuberos AJ, De la Torre AG, Alvarez-Pinazo G, Martín-Sedeño MC, Schollbach K, Pöllmann H, Aranda MA. Active iron-rich belite sulfoaluminate cements: clinkering and hydration. Environ Sci Technol. 2010 Sep 1;44(17):6855-62. doi: 10.1021/es101785n. PubMed PMID: 20701316.  374: Borges FT, Campos WR, Munari LS, Moreira AN, Paiva SM, Magalhães CS. Cariostatic effect of fluoride-containing restorative materials associated with fluoride gels on root dentin. J Appl Oral Sci. 2010 Sep-Oct;18(5):453-60. PubMed PMID: 21085800.  375: Chen X, Cuijpers V, Fan M, Frencken JE. Marginal leakage of two newer glass-ionomer-based sealant materials assessed using micro-CT. J Dent. 2010 Sep;38(9):731-5. doi: 10.1016/j.jdent.2010.05.018. Epub 2010 May 24. PubMed PMID: 20580767.  376: Farea M, Masudi S, Wan Bakar WZ. Apical microleakage evaluation of system B compared with cold lateral technique: In vitro study. Aust Endod J. 2010 Aug;36(2):48-53. doi: 10.1111/j.1747-4477.2009.00187.x. PubMed PMID: 20666748.  377: Fujimoto Y, Iwasa M, Murayama R, Miyazaki M, Nagafuji A, Nakatsuka T. Detection of ions released from S-PRG fillers and their modulation effect. Dent Mater J. 2010 Aug;29(4):392-7. Epub 2010 Jul 2. PubMed PMID: 20610878.  378: Portela A, Vasconcelos M, Branco R, Gartner F, Faria M, Cavalheiro J. An in vitro and in vivo investigation of the biological behavior of a ferromagnetic cement for highly focalized thermotherapy. J Mater Sci Mater Med. 2010 Aug;21(8):2413-23. doi: 10.1007/s10856-010-4093-6. Epub 2010 Jun 15. PubMed PMID: 20549312.  379: Wren AW, Kidari A, Cummins NM, Towler MR. A spectroscopic investigation into the setting and mechanical properties of titanium containing glass polyalkenoate cements. J Mater Sci Mater Med. 2010 Aug;21(8):2355-64. doi: 10.1007/s10856-010-4089-2. Epub 2010 May 13. PubMed PMID: 20464456.  380: Lee JJ, Lee YK, Choi BJ, Lee JH, Choi HJ, Son HK, Hwang JW, Kim SO. Physical properties of resin-reinforced glass ionomer cement modified with micro and nano-hydroxyapatite. J Nanosci Nanotechnol. 2010 Aug;10(8):5270-6. PubMed PMID: 21125881.  381: Knösel M, Mattysek S, Jung K, Kubein-Meesenburg D, Sadat-Khonsari R, Ziebolz D. Suitability of orthodontic brackets for rebonding and reworking following removal by air pressure pulses and conventional debracketing techniques. Angle Orthod. 2010 Jul;80(4):461-7. doi: 10.2319/102809-605.1. PubMed PMID: 20482349.  382: Maneenut C, Sakoolnamarka R, Tyas MJ. The repair potential of resin-modified glass-ionomer cements. Dent Mater. 2010 Jul;26(7):659-65. doi: 10.1016/j.dental.2010.03.009. Epub 2010 Apr 20. PubMed PMID: 20409575.  383: de Oliveira JL, Martins LM, Sanada J, de Oliveira PC, do Valle AL. The effect of framework design on fracture resistance of metal-ceramic implant-supported single crowns. Int J Prosthodont. 2010 Jul-Aug;23(4):350-2. PubMed PMID: 20617225.  384: Eisenburger M, Klug S. The effect of guidance elements on the rotation of cast crowns during cementation. Int J Prosthodont. 2010 Jul-Aug;23(4):347-9. PubMed PMID: 20617224.  385: Trairatvorakul C, Kangvansurakit N, Pathomburi J. In vitro comparison of self versus professionally applied remineralizing materials. J Clin Pediatr Dent. 2010 Summer;34(4):323-8. PubMed PMID: 20831134.  386: Subramaniam P, Kondae S, Gupta KK. Retentive strength of luting cements for stainless steel crowns: an in vitro study. J Clin Pediatr Dent. 2010 Summer;34(4):309-12. PubMed PMID: 20831131.  387: Xu HH, Moreau JL, Sun L, Chow LC. Novel CaF(2) nanocomposite with high strength and fluoride ion release. J Dent Res. 2010 Jul;89(7):739-45. doi: 10.1177/0022034510364490. Epub 2010 May 3. PubMed PMID: 20439933; PubMed Central  PMCID: PMC3077944.  388: Gill NC, Pathak A. Comparative evaluation of the effect of topical fluorides on the microhardness of various restorative materials: an in vitro study. J Indian Soc Pedod Prev Dent. 2010 Jul-Sep;28(3):193-9. doi: 10.4103/0970-4388.73784. PubMed PMID: 21157053.  389: Santamaria MP, Ambrosano GM, Casati MZ, Nociti FH Jr, Sallum AW, Sallum EA. The influence of local anatomy on the outcome of treatment of gingival recession associated with non-carious cervical lesions. J Periodontol. 2010 Jul;81(7):1027-34. doi: 10.1902/jop.2010.090366. PubMed PMID: 20214443.  390: Al-Maqtari AA, Lui JL. Effect of aging on coronal microleakage in access cavities through metal ceramic crowns restored with resin composites. J Prosthodont. 2010 Jul;19(5):347-56. doi: 10.1111/j.1532-849X.2010.00593.x. Epub 2010 Apr 23. PubMed PMID: 20456026.  391: Kwon OH, Kim DH, Park SH. The influence of elastic modulus of base material on the marginal adaptation of direct composite restoration. Oper Dent. 2010 Jul-Aug;35(4):441-7. doi: 10.2341/09-372-L. PubMed PMID: 20672729.  392: Zhang W, Masumi SI, Song XM. Bonding property of two resin-reinforced glass-ionomer cements to zirconia ceramic. Quintessence Int. 2010 Jul-Aug;41(7):e132-40. PubMed PMID: 20614036.  393: Uysal T, Yilmaz E, Ramoglu SI. Amorphous calcium phosphate-containing orthodontic cement for band fixation: an in vitro study. World J Orthod. 2010 Summer;11(2):129-34. PubMed PMID: 20552099.  394: Bagheri R, Azar MR, Tyas MJ, Burrow MF. The effect of aging on the fracture toughness of esthetic restorative materials. Am J Dent. 2010 Jun;23(3):142-6. PubMed PMID: 20718210.  395: Irie M, Maruo Y, Nishigawa G, Suzuki K, Watts DC. Physical properties of dual-cured luting-agents correlated to early no interfacial-gap incidence with composite inlay restorations. Dent Mater. 2010 Jun;26(6):608-15. doi: 10.1016/j.dental.2010.02.012. Epub 2010 Mar 23. PubMed PMID: 20334906.  396: Rusin RP, Agee K, Suchko M, Pashley DH. Effect of a new desensitising material on human dentin permeability. Dent Mater. 2010 Jun;26(6):600-7. doi: 10.1016/j.dental.2010.02.010. Epub 2010 Mar 20. PubMed PMID: 20304479.  397: Baysal A, Uysal T. Resin-modified glass ionomer cements for bonding orthodontic retainers. Eur J Orthod. 2010 Jun;32(3):254-8. doi: 10.1093/ejo/cjp066. Epub 2009 Sep 30. PubMed PMID: 19793779.  398: Topaloglu Ak A, Riza Alpoz A. Effect of saliva contamination on microleakage of three different pit and fissure sealants. Eur J Paediatr Dent. 2010 Jun;11(2):93-6. PubMed PMID: 20635844.  399: O'Brien T, Shoja-Assadi F, Lea SC, Burke FJ, Palin WM. Extrinsic energy sources affect hardness through depth during set of a glass-ionomer cement. J Dent. 2010 Jun;38(6):490-5. doi: 10.1016/j.jdent.2010.03.004. Epub 2010 Mar 17. PubMed PMID: 20302904.  400: Kirsten GA, Takahashi MK, Rached RN, Giannini M, Souza EM. Microhardness of dentin underneath fluoride-releasing adhesive systems subjected to cariogenic challenge and fluoride therapy. J Dent. 2010 Jun;38(6):460-8. doi: 10.1016/j.jdent.2010.02.006. Epub 2010 Mar 1. PubMed PMID: 20193726.  401: Nagas E, Uyanik O, Altundasar E, Durmaz V, Cehreli ZC, Vallittu PK, Lassila LV. Effect of different intraorifice barriers on the fracture resistance of roots obturated with Resilon or gutta-percha. J Endod. 2010 Jun;36(6):1061-3. doi: 10.1016/j.joen.2010.03.006. Epub 2010 Apr 9. PubMed PMID: 20478466.  402: Mulla Z, Edwards M, Nicholson JW. Release of sodium fusidate from glass-ionomer dental cement. J Mater Sci Mater Med. 2010 Jun;21(6):1997-2000. doi: 10.1007/s10856-010-4060-2. Epub 2010 Apr 8. PubMed PMID: 20376540.  403: Wadenya R, Smith J, Mante F. Microleakage of nano-particle-filled resin-modified glass ionomer using atraumatic restorative technique in primary molars. N Y State Dent J. 2010 Jun-Jul;76(4):36-9. PubMed PMID: 20863039.  404: Pithon MM, dos Santos RL. Does ozone water affect the bond strengths of orthodontic brackets? Aust Orthod J. 2010 May;26(1):73-7. PubMed PMID: 20575204.  405: E L, Irie M, Nagaoka N, Yamashiro T, Suzuki K. Mechanical properties of a resin-modified glass ionomer cement for luting: effect of adding spherical silica filler. Dent Mater J. 2010 May;29(3):253-61. Epub 2010 May 18. PubMed PMID: 20484829.  406: Imazato S, Horikawa D, Takeda K, Kiba W, Izutani N, Yoshikawa R, Hayashi M, Ebisu S, Nakano T. Proliferation and differentiation potential of pluripotent mesenchymal precursor C2C12 cells on resin-based restorative materials. Dent Mater J. 2010 May;29(3):341-6. Epub 2010 May 13. PubMed PMID: 20467157.  407: Schmitter M, Lippenberger S, Rues S, Gilde H, Rammelsberg P. Fracture resistance of incisor teeth restored using fibre-reinforced posts and threaded metal posts: effect of post length, location, pretreatment and cementation of the final restoration. Int Endod J. 2010 May;43(5):436-42. doi: 10.1111/j.1365-2591.2010.01705.x. PubMed PMID: 20518938.  408: Rodrigues FB, Paranhos MP, Spohr AM, Oshima HM, Carlini B, Burnett LH Jr. Fracture resistance of root filled molar teeth restored with glass fibre bundles. Int Endod J. 2010 May;43(5):356-62. doi: 10.1111/j.1365-2591.2009.01666.x. PubMed PMID: 20518927.  409: de Souza GM, Silva NR, Paulillo LA, De Goes MF, Rekow ED, Thompson VP. Bond strength to high-crystalline content zirconia after different surface treatments. J Biomed Mater Res B Appl Biomater. 2010 May;93(2):318-23. doi: 10.1002/jbm.b.31549. PubMed PMID: 20336733.  410: Prabhakar AR, Mahantesh T, Ahuja V. Comparison of retention and demineralization inhibition potential of adhesive banding cements in primary teeth. J Dent Child (Chic). 2010 May-Aug;77(2):66-71. PubMed PMID: 20819400.  411: Wadhwani C, Hess T, Faber T, Piñeyro A, Chen CS. A descriptive study of the radiographic density of implant restorative cements. J Prosthet Dent. 2010 May;103(5):295-302. doi: 10.1016/S0022-3913(10)60062-5. PubMed PMID: 20416413.  412: Yu H, Li Q, Lin Y, Buchalla W, Wang Y. Influence of carbamide peroxide on the flexural strength of tooth-colored restorative materials: an in vitro study at different environmental temperatures. Oper Dent. 2010 May-Jun;35(3):300-7. doi: 10.2341/09-139-L. PubMed PMID: 20533630.  413: Suzuki P, de Souza V, Holland R, Murata SS, Gomes-Filho JE, Dezan Junior E, Rodrigues Dos Passos T. Tissue reaction of the EndoREZ in root canal fillings short of or beyond an apical foramenlike communication. Oral Surg Oral Med Oral Pathol Oral Radiol Endod. 2010 May;109(5):e94-9. doi: 10.1016/j.tripleo.2009.12.047. PubMed PMID: 20416527.  414: Pameijer CH, Zmener O, Alvarez Serrano S, Garcia-Godoy F. Sealing properties of a calcium aluminate luting agent. Am J Dent. 2010 Apr;23(2):121-4. PubMed PMID: 20608305.  415: Ayad MF, Bahannan SA, Rosenstiel SF. Morphological characteristics of the interface between resin composite and glass-ionomer cement to thin-walled roots: a microscopic investigation. Am J Dent. 2010 Apr;23(2):103-7. PubMed PMID:  20608301.  416: Uysal T, Ramoglu SI, Ertas H, Ulker M. Microleakage of orthodontic band cement at the cement-enamel and cement-band interfaces. Am J Orthod Dentofacial Orthop. 2010 Apr;137(4):534-9. doi: 10.1016/j.ajodo.2008.03.025. PubMed PMID: 20362915.  417: Ahn SJ, Lim BS, Lee SJ. Surface characteristics of orthodontic adhesives and effects on streptococcal adhesion. Am J Orthod Dentofacial Orthop. 2010 Apr;137(4):489-95; discussion 13A. doi: 10.1016/j.ajodo.2008.05.015. PubMed PMID: 20362908.  418: Chatzistavrou E, Eliades T, Zinelis S, Athanasiou AE, Eliades G. Fluoride release from an orthodontic glass ionomer adhesive in vitro and enamel fluoride uptake in vivo. Am J Orthod Dentofacial Orthop. 2010 Apr;137(4):458.e1-8; discussion 458-9. doi: 10.1016/j.ajodo.2009.10.030. PubMed PMID: 20362902.  419: Beuer F, Edelhoff D, Gernet W, Naumann M. Parameters affecting retentive force of electroformed double-crown systems. Clin Oral Investig. 2010 Apr;14(2):129-35. doi: 10.1007/s00784-009-0271-7. Epub 2009 Apr 3. PubMed PMID: 19343381.  420: Lien W, Vandewalle KS. Physical properties of a new silorane-based restorative system. Dent Mater. 2010 Apr;26(4):337-44. doi: 10.1016/j.dental.2009.12.004. Epub 2010 Jan 6. PubMed PMID: 20053434.  421: Hashemipour MA, Mohammadpour A, Nassab SA. Transient thermal and stress analysis of maxillary second premolar tooth using an exact three-dimensional model. Indian J Dent Res. 2010 Apr-Jun;21(2):158-64. doi: 10.4103/0970-9290.66624. PubMed PMID: 20657080.  422: Sfondrini MF, Cacciafesta V, Noga E, Scribante A, Klersy C. In vitro bond strength evaluation of four orthodontic cements. J Adhes Dent. 2010 Apr;12(2):131-5. doi: 10.3290/j.jad.a17544. PubMed PMID: 20157669.  423: Brito CR, Velasco LG, Bonini GA, Imparato JC, Raggio DP. Glass ionomer cement hardness after different materials for surface protection. J Biomed Mater Res A. 2010 Apr;93(1):243-6. doi: 10.1002/jbm.a.32524. PubMed PMID: 19557791.  424: Pinheiro SL, Gallassi PC, Saldanha TC, Bincelli IN, Barros PP, Silva GH. Repairing collagen in dentin carious lesions. Influence of sealing the material: a morphometric study. J Clin Pediatr Dent. 2010 Spring;34(3):223-8. PubMed PMID: 20578659.  425: Munhoz T, Karpukhina N, Hill RG, Law RV, De Almeida LH. Setting of commercial glass ionomer cement Fuji IX by (27)Al and (19)F MAS-NMR. J Dent. 2010 Apr;38(4):325-30. doi: 10.1016/j.jdent.2009.12.005. Epub 2010 Jan 4. PubMed PMID: 20043973.  426: Baliga MS, Bhat SS. Effect of fluorides from various restorative materials on remineralization of adjacent tooth: an in vitro study. J Indian Soc Pedod Prev Dent. 2010 Apr-Jun;28(2):84-90. doi: 10.4103/0970-4388.66742. PubMed PMID: 20660973.  427: Ali MN, Edwards M, Nicholson JW. Zinc polycarboxylate dental cement for the controlled release of an active organic substance: proof of concept. J Mater Sci Mater Med. 2010 Apr;21(4):1249-53. doi: 10.1007/s10856-009-3957-0. Epub 2009 Dec 6. PubMed PMID: 19967407.  428: Hartwell GR, Loucks CA, Reavley BA. Bacterial leakage of provisional restorative materials used in endodontics. Quintessence Int. 2010 Apr;41(4):335-9. PubMed PMID: 20305868.  429: Chicri RO, Sasaki RT, Carvalho AS, Nouer PR, Lima-Arsati YB. Effect of enamel pretreatment on shear bond strength of brackets bonded with resin-modified glass-ionomer cement. World J Orthod. 2010 Spring;11(1):11-5. PubMed PMID: 20209171.  430: Parolia A, Kundabala M, Rao NN, Acharya SR, Agrawal P, Mohan M, Thomas M. A comparative histological analysis of human pulp following direct pulp capping with Propolis, mineral trioxide aggregate and Dycal. Aust Dent J. 2010 Mar;55(1):59-64. doi: 10.1111/j.1834-7819.2009.01179.x. PubMed PMID: 20415913.  431: Fan DH, Sun JY, Huang KJ. Direct electrochemistry of horseradish peroxidase on Nafion/[bmim]PF(6)/agarose composite film modified glassy carbon electrode. Colloids Surf B Biointerfaces. 2010 Mar 1;76(1):44-9. doi: 10.1016/j.colsurfb.2009.10.007. Epub 2009 Oct 14. PubMed PMID: 19896811.  432: Hewlett S, Wadenya RO, Mante FK. Bond strength of luting cements to core foundation materials. Compend Contin Educ Dent. 2010 Mar;31(2):140-6. PubMed PMID: 20344899.  433: Oliveira LC, Duarte S Jr, Araujo CA, Abrahão A. Effect of low-elastic modulus liner and base as stress-absorbing layer in composite resin restorations. Dent Mater. 2010 Mar;26(3):e159-69. doi: 10.1016/j.dental.2009.11.076. Epub 2009 Dec 23. PubMed PMID: 20031198.  434: Saku S, Kotake H, Scougall-Vilchis RJ, Ohashi S, Hotta M, Horiuchi S, Hamada K, Asaoka K, Tanaka E, Yamamoto K. Antibacterial activity of composite resin with glass-ionomer filler particles. Dent Mater J. 2010 Mar;29(2):193-8. PubMed PMID: 20379030.  435: Roggendorf MJ, Legner M, Ebert J, Fillery E, Frankenberger R, Friedman S. Micro-CT evaluation of residual material in canals filled with Activ GP or GuttaFlow following removal with NiTi instruments. Int Endod J. 2010 Mar;43(3):200-9. doi: 10.1111/j.1365-2591.2009.01659.x. PubMed PMID: 20158531.  436: Avşar A, Tuloglu N. Effect of different topical fluoride applications on the surface roughness of a colored compomer. J Appl Oral Sci. 2010 Mar-Apr;18(2):171-7. PubMed PMID: 20485929.  437: Rusin RP, Agee K, Suchko M, Pashley DH. Effect of a new liner/base on human dentin permeability. J Dent. 2010 Mar;38(3):245-52. doi: 10.1016/j.jdent.2009.11.004. Epub 2009 Nov 27. PubMed PMID: 19945499.  438: Kim YK, Yiu CK, Kim JR, Gu L, Kim SK, Weller RN, Pashley DH, Tay FR. Failure of a glass ionomer to remineralize apatite-depleted dentin. J Dent Res. 2010 Mar;89(3):230-5. doi: 10.1177/0022034509357172. Epub 2010 Jan 28. PubMed PMID: 20110510; PubMed Central PMCID: PMC2826886.  439: Delmé KI, Deman PJ, De Bruyne MA, Nammour S, De Moor RJ. Microleakage of glass ionomer formulations after erbium:yttrium-aluminium-garnet laser preparation. Lasers Med Sci. 2010 Mar;25(2):171-80. doi: 10.1007/s10103-008-0598-3. Epub 2008 Aug 21. PubMed PMID: 18716828.  440: Koubi S, Raskin A, Dejou J, About I, Tassery H, Camps J, Proust JP. Effect of dual cure composite as dentin substitute on the marginal integrity of Class II open-sandwich restorations. Oper Dent. 2010 Mar-Apr;35(2):165-71. doi: 10.2341/08-104-L. PubMed PMID: 20420059.  441: Marquezan M, Osorio R, Ciamponi AL, Toledano M. Resistance to degradation of bonded restorations to simulated caries-affected primary dentin. Am J Dent. 2010 Feb;23(1):47-52. PubMed PMID: 20437728.  442: Magni E, Ferrari M, Hickel R, Ilie N. Evaluation of the mechanical properties of dental adhesives and glass-ionomer cements. Clin Oral Investig. 2010 Feb;14(1):79-87. doi: 10.1007/s00784-009-0259-3. Epub 2009 Feb 25. PubMed PMID: 19241096.  443: Thanjal NK, Billington RW, Shahid S, Luo J, Hill RG, Pearson GJ. Kinetics of fluoride ion release from dental restorative glass ionomer cements: the influence of ultrasound, radiant heat and glass composition. J Mater Sci Mater Med. 2010 Feb;21(2):589-95. doi: 10.1007/s10856-009-3901-3. Epub 2009 Oct 23. PubMed PMID: 19851843.  444: Soares CJ, Raposo LH, Soares PV, Santos-Filho PC, Menezes MS, Soares PB, Magalhães D. Effect of different cements on the biomechanical behavior of teeth restored with cast dowel-and-cores-in vitro and FEA analysis. J Prosthodont. 2010 Feb;19(2):130-7. doi: 10.1111/j.1532-849X.2009.00527.x. Epub 2009 Dec 3. PubMed PMID: 20002972.  445: Coughlan A, Scanlon K, Mahon BP, Towler MR. Zinc and silver glass polyalkenoate cements: an evaluation of their antibacterial nature. Biomed Mater Eng. 2010;20(2):99-106. doi: 10.3233/BME-2010-0620. PubMed PMID: 20592447.  446: Maruo IT, Godoy-Bezerra J, Saga AY, Tanaka OM, Maruo H, Camargo ES. Effect of etching and light-curing time on the shear bond strength of a resin-modified glass ionomer cement. Braz Dent J. 2010;21(6):533-7. PubMed PMID: 21271044.  447: Garcia Lda F, Pires-de-Souza Fde C, Teófilo JM, Cestari A, Calefi PS, Ciuffi KJ, Nassar EJ. Synthesis and biocompatibility of an experimental glass ionomer cement prepared by a non-hydrolytic sol-gel method. Braz Dent J. 2010;21(6):499-507. PubMed PMID: 21271039.  448: Rodrigues E, Delbem AC, Pedrini D, Cavassan L. Enamel remineralization by fluoride-releasing materials: proposal of a pH-cycling model. Braz Dent J. 2010;21(5):446-51. PubMed PMID: 21180802.  449: Raggio DP, Bonifácio CC, Bönecker M, Imparato JC, Gee AJ, Amerongen WE. Effect of insertion method on knoop hardness of high viscous glass ionomer cements. Braz Dent J. 2010;21(5):439-45. PubMed PMID: 21180801.  450: dos Santos RL, Pithon MM, Vaitsman DS, Araújo MT, de Souza MM, Nojima MG. Long-term fluoride release from resin-reinforced orthodontic cements following recharge with fluoride solution. Braz Dent J. 2010;21(2):98-103. PubMed PMID: 20640354.  451: Durner J, Spahl W, Zaspel J, Schweikl H, Hickel R, Reichl FX. Eluted substances from unpolymerized and polymerized dental restorative materials and their Nernst partition coefficient. Dent Mater. 2010 Jan;26(1):91-9. doi: 10.1016/j.dental.2009.08.014. Epub. PubMed PMID: 19781758.  452: Lee YK, Yu B, Zhao GF, Lim JI. Effects of aging and HEMA content on the translucency, fluorescence, and opalescence properties of experimental HEMA-added glass ionomers. Dent Mater J. 2010 Jan;29(1):9-14. PubMed PMID: 20379006.  453: Prashanti E, Sajjan S, Kumar M. Comparison of stress patterns and displacement in conventional cantilever fixed partial denture with resin bonded cantilever fixed partial denture: a finite element analysis. Indian J Dent Res. 2010 Jan-Mar;21(1):59-62. doi: 10.4103/0970-9290.62797. PubMed PMID: 20427909.  454: Retamoso LB, Onofre NM, Hann L, Marchioro EM. Effect of light-curing units in shear bond strength of metallic brackets: an in vitro study. J Appl Oral Sci. 2010 Jan-Feb;18(1):68-74. PubMed PMID: 20379684.  455: Rodrigues Junior SA, Pin LF, Machado G, Della Bona A, Demarco FF. Influence of different restorative techniques on marginal seal of class II composite restorations. J Appl Oral Sci. 2010 Jan-Feb;18(1):37-43. PubMed PMID: 20379680.  456: Xie D, Zhao J, Weng Y. Synthesis and application of novel multi-arm poly(carboxylic acid)s for glass-ionomer restoratives. J Biomater Appl. 2010 Jan;24(5):419-36. doi: 10.1177/0885328208098593. Epub 2008 Nov 25. PubMed PMID: 19033326.  457: Zhang C, Degrange M. Shear bond strengths of self-adhesive luting resins fixing dentine to different restorative materials. J Biomater Sci Polym Ed. 2010;21(5):593-608. doi: 10.1163/156856209X431640. PubMed PMID: 20338094.  458: Fahmy AE, Farrag NM. Microleakage and shear punch bond strength in class II primary molars cavities restored with low shrink silorane based versus methacrylate based composite using three different techniques. J Clin Pediatr Dent. 2010 Winter;35(2):173-81. PubMed PMID: 21417120.  459: Sainulabdeen S, Neelakantan P, Ramesh S, Subbarao CV. Antibacterial activity of triclosan incorporated glass ionomer cements--an in vitro pilot study. J Clin Pediatr Dent. 2010 Winter;35(2):157-61. PubMed PMID: 21417117.  460: Wadenya RO, Yego C, Mante FK. Marginal microleakage of alternative restorative treatment and conventional glass ionomer restorations in extracted primary molars. J Dent Child (Chic). 2010 Jan-Apr;77(1):32-5. PubMed PMID: 20359427.  461: Bonifácio CC, van Amerongen WE, Meschini TG, Raggio DP, Bönecker M. Flowable glass ionomer cement as a liner: improving marginal adaptation of atraumatic restorative treatment restorations. J Dent Child (Chic). 2010 Jan-Apr;77(1):12-6. PubMed PMID: 20359424.  462: Berzins DW, Abey S, Costache MC, Wilkie CA, Roberts HW. Resin-modified glass-ionomer setting reaction competition. J Dent Res. 2010 Jan;89(1):82-6. doi: 10.1177/0022034509355919. Epub . PubMed PMID: 19966038; PubMed Central PMCID: PMC3318046.  463: Lewis G, Towler MR, Boyd D, German MJ, Wren AW, Clarkin OM, Yates A. Evaluation of two novel aluminum-free, zinc-based glass polyalkenoate cements as alternatives to PMMA bone cement for use in vertebroplasty and balloon kyphoplasty. J Mater Sci Mater Med. 2010 Jan;21(1):59-66. doi: 10.1007/s10856-009-3845-7. Epub 2009 Aug 5. PubMed PMID: 19655232.  464: Al-Omari WM, Zagibeh AM. The retention of cast metal dowels fabricated by direct and indirect techniques. J Prosthodont. 2010 Jan;19(1):58-63. doi: 10.1111/j.1532-849X.2009.00521.x. Epub 2009 Sep 17. PubMed PMID: 19765197.  465: Korkmaz Y, Gurgan S, Firat E, Nathanson D. Shear bond strength of three different nano-restorative materials to dentin. Oper Dent. 2010  Jan-Feb;35(1):50-7. doi: 10.2341/09-051-L. PubMed PMID: 20166411.  466: Matalon S, Peretz B, Sidon R, Weiss EI, Slutzky H. Antibacterial properties of pit and fissure sealants combined with daily fluoride mouth rinse. Pediatr Dent. 2010 Jan-Feb;32(1):9-13. PubMed PMID: 20298647.  467: Helmi K, Menard-Szczebara F, Lénès D, Jacob P, Jossent J, Barbot C, Delabre K, Arnal C. Adenovirus, MS2 and PhiX174 interactions with drinking water biofilms developed on PVC, cement and cast iron. Water Sci Technol. 2010;61(12):3198-207. doi: 10.2166/wst.2010.821. PubMed PMID: 20555217.  468: Capa N, Ozkurt Z, Canpolat C, Kazazoglu E. Shear bond strength of luting agents to fixed prosthodontic restorative core materials. Aust Dent J. 2009 Dec;54(4):334-40. doi: 10.1111/j.1834-7819.2009.01159.x. PubMed PMID: 20415932.  469: Bürgers R, Cariaga T, Müller R, Rosentritt M, Reischl U, Handel G, Hahnel S. Effects of aging on surface properties and adhesion of Streptococcus mutans on various fissure sealants. Clin Oral Investig. 2009 Dec;13(4):419-26. doi: 10.1007/s00784-009-0256-6. Epub 2009 Feb 21. PubMed PMID: 19234725.  470: Bortolotto T, Ferrari M, Susin A, Krejci I. Morphology of the smear layer after the application of simplified self-etch adhesives on enamel and dentin surfaces created with different preparation methods. Clin Oral Investig. 2009 Dec;13(4):409-17. doi: 10.1007/s00784-008-0242-4. Epub 2009 Jan 9. PubMed PMID: 19132414.  471: Zhao J, Platt JA, Xie D. Characterization of a novel light-cured star-shape poly(acrylic acid)-composed glass-ionomer cement: fluoride release, water sorption, shrinkage, and hygroscopic expansion. Eur J Oral Sci. 2009 Dec;117(6):755-65. doi: 10.1111/j.1600-0722.2009.00694.x. PubMed PMID: 20121941.  472: Mukai Y, Kamijo K, Fujino F, Hirata Y, Teranaka T, ten Cate JM. Effect of denture base-resin with prereacted glass-ionomer filler on dentin demineralization. Eur J Oral Sci. 2009 Dec;117(6):750-4. doi: 10.1111/j.1600-0722.2009.00678.x. PubMed PMID: 20121940.  473: Xie H, Yuan X, Wu F. Effects of surface treatments on bond strength between porcelain and electroformed gold substrates. J Adhes Dent. 2009 Dec;11(6):485-91. doi: 10.3290/j.jad.a18143. PubMed PMID: 20011769.  474: Yu H, Wegehaupt FJ, Wiegand A, Roos M, Attin T, Buchalla W. Erosion and abrasion of tooth-colored restorative materials and human enamel. J Dent. 2009 Dec;37(12):913-22. doi: 10.1016/j.jdent.2009.07.006. Epub 2009 Jul 24. PubMed PMID: 19674824.  475: Mourouzis P, Koulaouzidou EA, Vassiliadis L, Helvatjoglu-Antoniades M. Effects of sonic scaling on the surface roughness of restorative materials. J Oral Sci. 2009 Dec;51(4):607-14. PubMed PMID: 20032615.  476: Ayad MF, Johnston WM, Rosenstiel SF. Influence of tooth preparation taper and cement type on recementation strength of complete metal crowns. J Prosthet Dent. 2009 Dec;102(6):354-61. doi: 10.1016/S0022-3913(09)60192-X. PubMed PMID: 19961993.  477: Fahmy N, Naguib H, Guindy JE. Effect of light-emitting diode (LED) curing modes on resin/dentin bond strength. J Prosthodont. 2009 Dec;18(8):670-5. doi: 10.1111/j.1532-849X.2009.00502.x. Epub 2009 Aug 13. PubMed PMID: 19682222.  478: Borges GA, Caldas D, Taskonak B, Yan J, Sobrinho LC, de Oliveira WJ. Fracture loads of all-ceramic crowns under wet and dry fatigue conditions. J Prosthodont. 2009 Dec;18(8):649-55. doi: 10.1111/j.1532-849X.2009.00498.x. Epub 2009 Aug 4. PubMed PMID: 19682214.  479: Almilhatti HJ, Giampaolo ET, Vergani CE, Machado AL, Pavarina AC, Betiol EA. Adhesive bonding of resin composite to various Ni-Cr alloy surfaces using different metal conditioners and a surface modification system. J Prosthodont. 2009 Dec;18(8):663-9. doi: 10.1111/j.1532-849X.2009.00491.x. Epub 2009 Jun 11. PubMed PMID: 19523025.  480: Dimkov A, Nicholson JW, Gjorgievska E. On the possibility of incorporating antimicrobial components into glass-ionomer cements. Prilozi. 2009 Dec;30(2):219-37. PubMed PMID: 20087262.  481: Ahn HB, Ahn SJ, Lee SJ, Kim TW, Nahm DS. Analysis of surface roughness and surface free energy characteristics of various orthodontic materials. Am J Orthod Dentofacial Orthop. 2009 Nov;136(5):668-74. doi: 10.1016/j.ajodo.2007.11.032. PubMed PMID: 19892283.  482: Jagdish N, Padmanabhan S, Chitharanjan AB, Revathi J, Palani G, Sambasivam M, Sheriff K, Saravanamurali K. Cytotoxicity and degree of conversion of orthodontic adhesives. Angle Orthod. 2009 Nov;79(6):1133-8. doi: 10.2319/080808-418R.1. PubMed PMID: 19852605.  483: Coutinho E, Jarmar T, Svahn F, Neves AA, Verlinden B, Van Meerbeek B, Engqvist H. Ultrastructural characterization of tooth-biomaterial interfaces prepared with broad and focused ion beams. Dent Mater. 2009 Nov;25(11):1325-37. doi: 10.1016/j.dental.2009.06.002. Epub 2009 Jul 10. PubMed PMID: 19596422.  484: Coutinho E, Cardoso MV, De Munck J, Neves AA, Van Landuyt KL, Poitevin A, Peumans M, Lambrechts P, Van Meerbeek B. Bonding effectiveness and interfacial characterization of a nano-filled resin-modified glass-ionomer. Dent Mater. 2009 Nov;25(11):1347-57. doi: 10.1016/j.dental.2009.06.004. PubMed PMID: 19595446.  485: Ji W, Chen Z, Frencken JE. Strength of tunnel-restored teeth with different materials and marginal ridge height. Dent Mater. 2009 Nov;25(11):1363-70. doi: 10.1016/j.dental.2009.06.007. PubMed PMID: 19595444.  486: Tanomaru-Filho M, Spinola SG, Reis JM, Chavez-Andrade GM, Guerreiro-Tanomaru JM. In vitro sealing ability of temporary restorative materials used in endodontics. Gen Dent. 2009 Nov-Dec;57(6):622-5. PubMed PMID: 19906614.  487: Pirani C, Iacono F, Chersoni S, Sword J, Pashley DH, Tay FR, Looney S, Gandolfi MG, Prati C. The effect of ultrasonic removal of various root-end filling materials. Int Endod J. 2009 Nov;42(11):1015-25. doi: 10.1111/j.1365-2591.2009.01612.x. PubMed PMID: 19825036.  488: Moosavi H, Ghavamnasiri M, Manari V. Effect of postoperative bleaching on marginal leakage of resin composite and resin-modified glass ionomer restorations at different delayed periods of exposure to carbamide peroxide. J Contemp Dent Pract. 2009 Nov 1;10(6):E009-16. PubMed PMID: 20020076.  489: Marquezan M, Fagundes TC, Toledano M, Navarro MF, Osorio R. Differential bonds degradation of two resin-modified glass-ionomer cements in primary and permanent teeth. J Dent. 2009 Nov;37(11):857-64. doi: 10.1016/j.jdent.2009.06.018. Epub 2009 Jul 4. PubMed PMID: 19631441.  490: Alencar CJ, Braga MM, de Oliveira E, Nicolau J, Mendes FM. Dye-enhanced laser fluorescence detection of caries lesions around brackets. Lasers Med Sci. 2009 Nov;24(6):865-70. doi: 10.1007/s10103-008-0572-0. Epub 2008 Jun 7. PubMed PMID: 18536957.  491: Perez Cdos R, Hirata RJ, da Silva AH, Sampaio EM, de Miranda MS. Effect of a glaze/composite sealant on the 3-D surface roughness of esthetic restorative materials. Oper Dent. 2009 Nov-Dec;34(6):674-80. doi: 10.2341/08-014-L. PubMed PMID: 19953776.  492: Chen YL, Shih PH, Chiang LC, Chang YK, Lu HC, Chang JE. The influence of heavy metals on the polymorphs of dicalcium silicate in the belite-rich clinkers produced from electroplating sludge. J Hazard Mater. 2009 Oct 15;170(1):443-8. doi: 10.1016/j.jhazmat.2009.04.076. Epub 2009 Apr 24. PubMed PMID: 19464111.  493: Chin MY, Sandham A, Rumachik EN, Ruben JL, Huysmans MC. Fluoride release and  cariostatic potential of orthodontic adhesives with and without daily fluoride rinsing. Am J Orthod Dentofacial Orthop. 2009 Oct;136(4):547-53. doi: 10.1016/j.ajodo.2007.10.053. PubMed PMID: 19815157.  494: Shintome LK, Nagayassu MP, Di Nicoló R, Myaki SI. Microhardness of glass ionomer cements indicated for the ART technique according to surface protection treatment and storage time. Braz Oral Res. 2009 Oct-Dec;23(4):439-45. PubMed PMID: 20027452.  495: Lugato IC, Pignatta LM, Arantes Fde M, Santos EC. Comparison of the shear bond strengths of conventional mesh bases and sandblasted orthodontic bracket bases. Braz Oral Res. 2009 Oct-Dec;23(4):407-14. PubMed PMID: 20027448.  496: Moshaverinia A, Roohpour N, Ansari S, Moshaverinia M, Schricker S, Darr JA, Rehman IU. Effects of N-vinylpyrrolidone (NVP) containing polyelectrolytes on surface properties of conventional glass-ionomer cements (GIC). Dent Mater. 2009 Oct;25(10):1240-7. doi: 10.1016/j.dental.2009.05.006. Epub 2009 Jun 24. PubMed PMID: 19556000.  497: Magne P, Knezevic A. Thickness of CAD-CAM composite resin overlays influences fatigue resistance of endodontically treated premolars. Dent Mater. 2009 Oct;25(10):1264-8. doi: 10.1016/j.dental.2009.05.007. Epub 2009 Jun 18. PubMed PMID: 19539358.  498: Pieper CM, Zanchi CH, Rodrigues-Junior SA, Moraes RR, Pontes LS, Bueno M. Sealing ability, water sorption, solubility and toothbrushing abrasion resistance of temporary filling materials. Int Endod J. 2009 Oct;42(10):893-9. doi: 10.1111/j.1365-2591.2009.01590.x. Epub 2009 Jun 22. PubMed PMID: 19549149.  499: Schmage P, Nergiz I, Sito F, Platzer U, Rosentritt M. Wear and hardness of different core build-up materials. J Biomed Mater Res B Appl Biomater. 2009 Oct;91(1):71-9. doi: 10.1002/jbm.b.31375. PubMed PMID: 19402146.  500: Yesilyurt C, Yildirim T, Taşdemir T, Kusgoz A. Shear bond strength of conventional glass ionomer cements bound to mineral trioxide aggregate. J Endod. 2009 Oct;35(10):1381-3. doi: 10.1016/j.joen.2009.06.003. Epub 2009 Aug 6. PubMed PMID: 19801234.  501: Rohilla M, Pandit IK, Srivastava N. Why the carioprotective potential of luting cements crucial? J Indian Soc Pedod Prev Dent. 2009 Oct-Dec;27(4):219-23. doi: 10.4103/0970-4388.57656. PubMed PMID: 19915272.  502: Wren A, Clarkin OM, Laffir FR, Ohtsuki C, Kim IY, Towler MR. The effect of glass synthesis route on mechanical and physical properties of resultant glass ionomer cements. J Mater Sci Mater Med. 2009 Oct;20(10):1991-9. doi: 10.1007/s10856-009-3781-6. Epub 2009 May 21. PubMed PMID: 19459033.  503: Ciftçi A, Vardarli DA, Sönmez IS. Coronal microleakage of four endodontic temporary restorative materials: an in vitro study. Oral Surg Oral Med Oral Pathol Oral Radiol Endod. 2009 Oct;108(4):e67-70. doi: 10.1016/j.tripleo.2009.05.015. Epub 2009 Aug 28. PubMed PMID: 19716727.  504: Bonifácio CC, Kleverlaan CJ, Raggio DP, Werner A, de Carvalho RC, van Amerongen WE. Physical-mechanical properties of glass ionomer cements indicated for atraumatic restorative treatment. Aust Dent J. 2009 Sep;54(3):233-7. doi: 10.1111/j.1834-7819.2009.01125.x. PubMed PMID: 19709111.  505: Sadaghiani L, Adusei G, Rees J. Effects of storage media on physical properties of selected tooth coloured restorative materials. Eur J Prosthodont Restor Dent. 2009 Sep;17(3):116-20. PubMed PMID: 19839187.  506: Assis CP, Moyses MR, Teixeira HM, Ribeiro JC, Ribeiro JG, Dias SC. Fatigue limits for composite restorations with and without glass ionomer cement liners. Gen Dent. 2009 Sep-Oct;57(5):485-9; quiz 490-1, 535-6. PubMed PMID: 19903639.  507: Johnson GH, Lepe X, Zhang H, Wataha JC. Retention of metal-ceramic crowns with contemporary dental cements. J Am Dent Assoc. 2009 Sep;140(9):1125-36. PubMed PMID: 19723946.  508: Saraç D, Külünk S, Saraç YS, Karakas O. Effect of fluoride-containing desensitizing agents on the bond strength of resin-based cements to dentin. J Appl Oral Sci. 2009 Sep-Oct;17(5):495-500. PubMed PMID: 19936532.  509: Thomé T, Mayer MP, Imazato S, Geraldo-Martins VR, Marques MM. In vitro analysis of inhibitory effects of the antibacterial monomer MDPB-containing restorations on the progression of secondary root caries. J Dent. 2009 Sep;37(9):705-11. doi: 10.1016/j.jdent.2009.05.024. Epub 2009 May 28. PubMed PMID: 19540033.  510: Limapornvanich A, Jitpukdeebodintra S, Hengtrakool C, Kedjarune-Leggat U. Bovine serum albumin release from novel chitosan-fluoro-aluminosilicate glass ionomer cement: stability and cytotoxicity studies. J Dent. 2009 Sep;37(9):686-90. doi: 10.1016/j.jdent.2009.05.007. Epub 2009 May 14. PubMed PMID: 19501445.  511: Bertolini MJ, Zaghete MA, Gimenes R, Padovani GC, Cruz CA. Preparation and evaluation of an experimental luting glass ionomer cement to be used in dentistry. J Mater Sci Mater Med. 2009 Sep;20(9):1781-5. doi: 10.1007/s10856-009-3748-7. Epub 2009 May 5. PubMed PMID: 19415231.  512: Khoroushi M, Fardashtaki SR. Effect of light-activated bleaching on the microleakage of Class V tooth-colored restorations. Oper Dent. 2009 Sep-Oct;34(5):565-70. doi: 10.2341/08-050-L. PubMed PMID: 19830971.  513: Gorduysus M, Avcu N. Evaluation of the radiopacity of different root canal sealers. Oral Surg Oral Med Oral Pathol Oral Radiol Endod. 2009 Sep;108(3):e135-40. doi: 10.1016/j.tripleo.2009.04.016. Epub 2009 Jul 3. PubMed PMID: 19577493. | 1/1: Oskoee SS, Bahari M, Kimyai S, Rikhtegaran S, Puralibaba F, Ajami H. Effect of Nd:YAG laser pulse energy on mercury vapor release from the dental amalgam. Photomed Laser Surg. 2013 Oct;31(10):480-5. doi: 10.1089/pho.2013.3549. Epub 2013 Sep 21. PubMed PMID: 24053716.  2/2: Mirza AJ, Ahmad A, Mohammad T, Khan ZA. Evaluation of various concentrations of alkaline surface treatment on interfacial bond strengths of amalgam bonded to amalgam. Pak J Pharm Sci. 2013 Sep;26(5):1033-6. PubMed PMID: 24035964.  3/3: Scholtanus JD, van der Hoorn W, Ozcan M, Huysmans MC, Roeters JF, Kleverlaan CJ, Feilzer AJ. Staining of dentin from amalgam corrosion is induced by  demineralization. Am J Dent. 2013 Aug;26(4):185-90. PubMed PMID: 24693627.  4/4: Soussa E, Shalaby Y, Maria AM, Maria OM. Evaluation of oral tissue response and blood levels of mercury released from dental amalgam in rats. Arch Oral Biol. 2013 Aug; 58(8):981-8. doi: 10.1016/ j.archoralbio.2013.03.012. Epub 2013 Apr 20. PubMed PMID: 23611063.  5/5: Wang Y, Goodrich JM, Werner R, Gillespie B, Basu N, Franzblau A. Relationship of estimated dietary intake of n-3 polyunsaturated fatty acids from fish with peripheral nerve function after adjusting for mercury exposure. Sci Total Environ. 2013 Jun 1;454-455:73-8. doi: 10.1016/j.scitotenv.2013.02.075. Epub 2013 Mar 26. PubMed PMID: 23538138; PubMed Central PMCID: PMC3640748.  6/6: Sherman LS, Blum JD, Franzblau A, Basu N. New insight into biomarkers of human mercury exposure using naturally occurring mercury stable isotopes. Environ Sci  Technol. 2013 Apr 2;47(7):3403-9. doi: 10.1021/es305250z. Epub 2013 Mar 20. PubMed PMID: 23463943.  7/7: Guneser MB, Akbulut MB, Eldeniz AU. Effect of various endodontic irrigants on the push-out bond strength of biodentine and conventional root perforation repair materials. J Endod. 2013 Mar;39(3):380-4. doi: 10.1016/j.joen.2012.11.033. Epub 2013 Jan 16. PubMed PMID: 23402511.  8/8: Murat S, Kamburoğlu K, Isayev A, Kurşun S, Yüksel S. Visibility of artificial buccal recurrent caries under restorations using different radiographic techniques. Oper Dent. 2013 Mar-Apr;38(2):197-207. doi: 10.2341/12-158-L. Epub 2012 Aug 23. PubMed PMID: 22917443.  9/9: Formosa LM, Mallia B, Camilleri J. A quantitative method for determining the antiwashout characteristics of cement-based dental materials including mineral trioxide aggregate. Int Endod J. 2013 Feb;46(2):179-86. doi: 10.1111/j.1365-2591.2012.02108.x. Epub 2012 Jul 27. PubMed PMID: 22845340.  10/10: Choi YJ, Razzoog ME. Masking ability of zirconia with and without veneering porcelain. J Prosthodont. 2013 Feb;22(2):98-104. doi: 10.1111/j.1532-849X.2012.00915.x. PubMed PMID: 23387963.  11/11: Yilmaz S, Misirlioglu M. The effect of 3 T MRI on microleakage of amalgam restorations. Dentomaxillofac Radiol. 2013;42(8):20130072. doi: 10.1259/dmfr.20130072. Epub 2013 May 14. PubMed PMID: 23674614; PubMed Central PMCID: PMC3756742.  12/12: Tymofiyeva O, Vaegler S, Rottner K, Boldt J, Hopfgartner AJ, Proff PC, Richter EJ, Jakob PM. Influence of dental materials on dental MRI. Dentomaxillofac Radiol. 2013;42(6):20120271. doi: 10.1259/dmfr.20120271. Epub 2013 Apr 22. PubMed PMID: 23610088; PubMed Central PMCID: PMC3667526.  13/13: Salomone P, Bueno RP, Trinidade RF, Nascimento PC, Pozzobon RT. Assessment of the release of mercury from silver amalgam alloys exposed to different 10% carbamide peroxide bleaching agents. Gen Dent. 2013 Jan-Feb;61(1):33-5. Erratum in: Gen Dent. 2013 Mar-Apr;61(2):75. PubMed PMID: 23302360.  14/14: Pospiech P, Nagel F, Gebhart F, Nothdurft FP, Mitov G. In vitro investigation of the performance of different restorative materials under cast circumferential  clasps for removable dental prostheses. Clin Oral Investig. 2012 Dec;16(6):1659-67. doi: 10.1007/s00784-011-0653-5. Epub 2011 Dec 17. PubMed PMID: 22173722.  15/15: Darvell BW. Development of strength in dental silver amalgam. Dent Mater. 2012 Oct;28(10):e207-17. doi: 10.1016/j.dental.2012.05.002. Epub 2012 Jun 5. PubMed PMID: 22677140.  16/16: Darvell BW. Effect of corrosion on the strength of dental silver amalgam. Dent Mater. 2012 Sep;28(9):e160-7. doi: 10.1016/j.dental.2012.06.001. Epub 2012 Jul 4. PubMed PMID: 22770402.  17/17: Pilo R, Nissan J, Shafir H, Shapira G, Alter E, Brosh T. The influence of long term water immersion on shear bond strength of amalgam repaired by resin composite and mediated by adhesives or resin modified glass ionomers. J Dent. 2012 Jul;40(7):594-602. doi: 10.1016/j.jdent.2012.04.001. Epub 2012 Apr 12. PubMed PMID: 22504527.  18/18: Al Ghadban A, Al Shaarani F. Antibacterial properties of amalgam and composite resin materials used as cores under crowns. Eur J Prosthodont Restor Dent. 2012 Jun;20(2):71-6. PubMed PMID: 22852523.  19/19: Neuhaus KW, Rodrigues JA, Seemann R, Lussi A. Detection of proximal secondary caries at cervical class II-amalgam restoration margins in vitro. J Dent. 2012 Jun;40(6):493-9. doi: 10.1016/j.jdent.2012.02.014. Epub 2012 Mar 17. PubMed PMID: 22429927.  20/20: Kilinc E, Rothrock J, Migliorati E, Drukteinis S, Roshkind DM, Bradley P. Potential surface alteration effects of laser-assisted periodontal surgery on existing dental restorations. Quintessence Int. 2012 May;43(5):387-95. PubMed PMID: 22536590.  21/21: Bahadure RN, Pandey RK, Kumar R, Gopal K, Singh RK. An estimation of fluoride release from various dental restorative materials at different pH: In vitro study. J Indian Soc Pedod Prev Dent. 2012 Apr-Jun;30(2):122-6. doi: 10.4103/0970-4388.99983. PubMed PMID: 22918096.  22/22: Ghavamnasiri M, Motamed-Sanaye V, Chasteen J, Ameri H, Hajizadeh H, Khashyarmanesh Z. Energy dispersive x-ray analysis of corrosion products in nondiscolored dentin and a dye-extraction study of Class 2 composite restorations following amalgam removal. Quintessence Int. 2012 Apr;43(4):325-32. PubMed PMID: 22532947.  23/23: Varghese S, Ariga P, Padmanaban TV, Subramanian R. A finite element thermal analysis of various dowel and core materials. Indian J Dent Res. 2012  Mar-Apr;23(2):176-81. doi: 10.4103/0970-9290.100422. PubMed PMID: 22945706.  24/24: Hakki SS, Bozkurt SB, Ozcopur B, Purali N, Belli S. Periodontal ligament fibroblast response to root perforations restored with different materials: a laboratory study. Int Endod J. 2012 Mar;45(3):240-8. doi: 10.1111/j.1365-2591.2011.01968.x. Epub 2011 Oct 19. PubMed PMID: 22007644.  25/25: Oskoee PA, Kachoei M, Rikhtegaran S, Fathalizadeh F, Navimipour EJ. Effect of surface treatment with sandblasting and Er,Cr:YSGG laser on bonding of stainless steel orthodontic brackets to silver amalgam. Med Oral Patol Oral Cir Bucal. 2012 Mar 1;17(2):e292-6. PubMed PMID: 22143706; PubMed Central PMCID: PMC3448331.  26/26: Zogheib CM, Hardan L, Khoury CK, Naaman NB. [Amalgam, composite and compomer: microbiological study]. Odontostomatol Trop. 2012 Mar;35(137):44-50. French. PubMed PMID: 22715643.  27/27: Arita ES, Silveira GP, Cortes AR, Brucoli HC. Comparative study between the radiopacity levels of high viscosity and of flowable composite resins, using digital imaging. Eur J Esthet Dent. 2012 Winter;7(4):430-8. PubMed PMID: 23150871.  28/28: Shah P, Gugwad SC, Bhat C, Lodaya R. Effect of three different core materials on the fracture resistance of endodontically treated deciduous mandibular second molars: an in vitro study. J Contemp Dent Pract. 2012 Jan 1;13(1):66-70. PubMed PMID: 22430696.  29/29: Blum IR, Hafiana K, Curtis A, Barbour ME, Attin T, Lynch CD, Jagger DC. The effect of surface conditioning on the bond strength of resin composite to amalgam. J Dent. 2012 Jan;40(1):15-21. doi: 10.1016/j.jdent.2011.10.019. Epub 2011 Nov 7. PubMed PMID: 22100436.  30/30: Takahashi Y, Tsuruta S, Honda A, Fujiwara Y, Satoh M, Yasutake A. Effect of dental amalgam on gene expression profiles in rat cerebrum, cerebellum, liver and  kidney. J Toxicol Sci. 2012;37(3):663-6. PubMed PMID: 22688007.  31/31: Klinke T, Daboul A, Maron J, Gredes T, Puls R, Jaghsi A, Biffar R. Artifacts in magnetic resonance imaging and computed tomography caused by dental materials. PLoS One. 2012;7(2):e31766. doi: 10.1371/journal.pone.0031766. Epub 2012 Feb 22. PubMed PMID: 22384071; PubMed Central PMCID: PMC3285178.  32/32: Popoff DA, Gonçalves FS, Magalhães CS, Moreira AN, Ferreira RC, Mjör IA. Repair of amalgam restorations with composite resin and bonded amalgam: a  microleakage study. Indian J Dent Res. 2011 Nov-Dec;22(6):799-803. doi: 10.4103/0970-9290.94672. PubMed PMID: 22484874.  33/33: Soares CJ, Roscoe MG, Castro CG, Santana FR, Raposo LH, Quagliatto PS, Novais VR. Effect of gamma irradiation and restorative material on the biomechanical  behaviour of root filled premolars. Int Endod J. 2011 Nov;44(11):1047-54. doi: 10.1111/j.1365-2591.2011.01920.x. Epub 2011 Jul 11. PubMed PMID: 21740445.  34/34: Jakovljević A, Pešić D, Popović M, Melih I. [Influence of different bonding agents on marginal sealing quality of amalgam restorations]. Srp Arh Celok Lek. 2011 Nov-Dec;139(11-12):722-7. Serbian. PubMed PMID: 22338466.  35/35: Libonati A, Marzo G, Klinger FG, Farini D, Gallusi G, Tecco S, Mummolo S, De Felici M, Campanella V. Embryotoxicity assays for leached components from dental restorative materials. Reprod Biol Endocrinol. 2011 Oct 6;9:136. doi: 10.1186/1477-7827-9-136. PubMed PMID: 21978455; PubMed Central PMCID: PMC3204284.  36/36: Bittar DG, Murakami C, Hesse D, Imparato JC, Mendes FM. Efficacy of two methods for restorative materials' removal in primary teeth. J Contemp Dent Pract. 2011 Sep 1;12(5):372-8. PubMed PMID: 22269199.  37/37: Gómez JA, Marques T, Kinoshita A, Belmonte G, Nicolucci P, Baffa O. Influence of dental restorative materials on ESR biodosimetry in tooth enamel. Radiat Res. 2011 Aug;176(2):259-63. Epub 2011 Jun 1. PubMed PMID: 21631287.  38/38: Shafiei F, Memarpour M, Karimi F. Fracture resistance of cuspal coverage of endodontically treated maxillary premolars with combined composite-amalgam  compared to other techniques. Oper Dent. 2011 Jul-Aug;36(4):439-47. doi: 10.2341/11-029-L. Epub 2011 Aug 5. PubMed PMID: 21819198.  39/39: Idiyatullin D, Corum C, Moeller S, Prasad HS, Garwood M, Nixdorf DR. Dental magnetic resonance imaging: making the invisible visible. J Endod. 2011 Jun;37(6):745-52. doi: 10.1016/j.joen.2011.02.022. Epub 2011 Apr 6. PubMed PMID: 21787482; PubMed Central PMCID: PMC3146019.  40/40: Aghdaee NA, Darban JG, Mohajeri A. Fracture strength in restored teeth before and after load cycling: an in vitro study. J Calif Dent Assoc. 2011 May;39(5):300-7. PubMed PMID: 21721474.  41/41: Gupta I, Gupta S, Kothari A. Revisiting amalgam: a comparative study between bonded amalgam restoration and amalgam retained with undercuts. J Contemp Dent Pract. 2011 May 1;12(3):164-70. PubMed PMID: 22186810.  42/42: Al-Hezaimi K, Al-Tayar BA, Bajuaifer YS, Salameh Z, Al-Fouzan K, Tay FR. A hybrid approach to direct pulp capping by using emdogain with a capping material. J Endod. 2011 May;37(5):667-72. doi: 10.1016/j.joen.2011.02.003. PubMed PMID: 21496668.  43/43: Ozcan M, Koolman C, Aladag A, Dündar M. Effects of different surface conditioning methods on the bond strength of composite resin to amalgam. Oper Dent. 2011 May-Jun;36(3):318-25. doi: 10.2341/10-294-L. Epub 2011 Jul 8. PubMed PMID: 21740243.  44/44: Chern Lin JH, Chen FY, Chiang HJ, Ju CP. Effect of ball milling on structures and properties of dispersed-type dental amalgam. Dent Mater. 2011 Apr;27(4):e65-79. doi: 10.1016/j.dental.2010.11.013. Epub 2010 Dec 21. PubMed PMID: 21176947.  45/45: Karam JR, Rinchuse DJ. Dental amalgam corrosion in vacuum-formed retainers. Orthodontics (Chic.). 2011 Spring;12(1):70-4. PubMed PMID: 21789292.  46/46: Hengtrakool C, Kukiattrakoon B, Kedjarune-Leggat U. Gradual surface degradation of restorative materials by acidic agents. Gen Dent. 2011 Mar-Apr;59(2):e50-62. PubMed PMID: 21903509.  47/47: Vasudeva G, Bogra P, Nikhil V, Singh V. Effect of occlusal restoration on stresses around class V restoration interface: a finite-element study. Indian J Dent Res. 2011 Mar-Apr;22(2):295-302. doi: 10.4103/0970-9290.84308. PubMed PMID: 21891903.  48/48: Parolia A, Kundabala M, Gupta V, Verma M, Batra C, Shenoy R, Srikant N. Microleakage of bonded amalgam restorations using different adhesive agents with  dye under vacuum: an in vitro study. Indian J Dent Res. 2011 Mar-Apr;22(2):252-5. doi: 10.4103/0970-9290.84298. PubMed PMID: 21891895.  49/49: Fayazi S, Ostad SN, Razmi H. Effect of ProRoot MTA, Portland cement, and amalgam on the expression of fibronectin, collagen I, and TGFβ by human periodontal ligament fibroblasts in vitro. Indian J Dent Res. 2011 Mar-Apr;22(2):190-4. doi: 10.4103/0970-9290.84278. PubMed PMID: 21891883.  50/50: Zavanelli AC, Mazaro VQ, Silva CR, Zavanelli RA, Mancuso DN. Surface roughness analysis of four restorative materials exposed to 10% and 15% carbamide  peroxide. Int J Prosthodont. 2011 Mar-Apr;24(2):155-7. PubMed PMID: 21479284.  51/51: Diniz MB, Lima LM, Eckert G, Zandona AG, Cordeiro RC, Pinto LS. In vitro evaluation of ICDAS and radiographic examination of occlusal surfaces and their  association with treatment decisions. Oper Dent. 2011 Mar-Apr;36(2):133-42. doi: 10.2341/10-006-L. PubMed PMID: 21777096.  52/52: Alizadeh K, Parooi R, Hashemi P, Rezaei B, Ganjali MR. A new Schiff's base ligand immobilized agarose membrane optical sensor for selective monitoring of mercury ion. J Hazard Mater. 2011 Feb 28;186(2-3):1794-800. doi: 10.1016/j.jhazmat.2010.12.067. Epub 2010 Dec 21. PubMed PMID: 21232854.  53/53: Heintze SD, Barkmeier WW, Latta MA, Rousson V. Round robin test: wear of nine dental restorative materials in six different wear simulators - supplement to the round robin test of 2005. Dent Mater. 2011 Feb;27(2):e1-9. doi: 10.1016/j.dental.2010.09.003. PubMed PMID: 20888629.  54/54: Asvanund P, Morgano SM. Photoelastic stress analysis of different prefabricated post-and-core materials. Dent Mater J. 2011;30(5):684-90. Epub 2011  Sep 23. PubMed PMID: 21946489.  55/55: Cianconi L, Conte G, Mancini M. Shear bond strength, failure modes, and confocal microscopy of bonded amalgam restorations. Dent Mater J. 2011;30(2):216-21. Epub 2011 Mar 4. PubMed PMID: 21383518.  56/56: Anbiaee N, Mohassel AR, Imanimoghaddam M, Moazzami SM. A comparison of the accuracy of digital and conventional radiography in the diagnosis of recurrent caries. J Contemp Dent Pract. 2010 Dec 1;11(6):E025-32. PubMed PMID: 21203734.  57/57: Kasraei S, Rezaei-Soufi L, Azarsina M. The effect of a 16% carbamide peroxide gel on mercury and silver ion release from admixed and spherical dental amalgams.  J Contemp Dent Pract. 2010 Dec 1;11(6):E009-16. PubMed PMID: 21203732.  58/58: Khani H, Rofouei MK, Arab P, Gupta VK, Vafaei Z. Multi-walled carbon nanotubes-ionic liquid-carbon paste electrode as a super selectivity sensor: application to potentiometric monitoring of mercury ion(II). J Hazard Mater. 2010 Nov 15;183(1-3):402-9. doi: 10.1016/j.jhazmat.2010.07.039. Epub 2010 Jul 15. PubMed PMID: 20692088.  59/59: Ozcan M, Schoonbeek G, Gökçe B, Cömlekoglu E, Dündar M. Bond strength comparison of amalgam repair protocols using resin composite in situations with and without dentin exposure. Oper Dent. 2010 Nov-Dec;35(6):655-62. doi: 10.2341/10-091-L. PubMed PMID: 21180005.  60/60: Alptekin T, Ozer F, Unlu N, Cobanoglu N, Blatz MB. In vivo and in vitro evaluations of microleakage around Class I amalgam and composite restorations. Oper Dent. 2010 Nov-Dec;35(6):641-8. doi: 10.2341/10-065-L. PubMed PMID: 21180003.  61/61: Mo SS, Bao W, Lai GY, Wang J, Li MY. The microfloral analysis of secondary caries biofilm around Class I and Class II composite and amalgam fillings. BMC Infect Dis. 2010 Aug 17;10:241. doi: 10.1186/1471-2334-10-241. PubMed PMID: 20712908; PubMed Central PMCID: PMC2931511.  62/62: Hitz T, Ozcan M, Göhring TN. Marginal adaptation and fracture resistance of root-canal treated mandibular molars with intracoronal restorations: effect of thermocycling and mechanical loading. J Adhes Dent. 2010 Aug;12(4):279-86. doi: 10.3290/j.jad.a17712. PubMed PMID: 20157655.  63/63: Baek SH, Lee WC, Setzer FC, Kim S. Periapical bone regeneration after endodontic microsurgery with three different root-end filling materials: amalgam, SuperEBA, and mineral trioxide aggregate. J Endod. 2010 Aug;36(8):1323-5. doi: 10.1016/j.joen.2010.04.008. Epub 2010 Jun 14. PubMed PMID: 20647089.  64/64: Wang Y, Darvell BW. Interactive effect of indenter size and specimen thickness in Hertzian indentation test. Dent Mater. 2010 Jun;26(6):539-44. doi:  10.1016/j.dental.2010.02.001. Epub 2010 Mar 29. PubMed PMID: 20303159.  65/65: Kournetas N, Kakaboura A, Giftopoulos D, Chakmachi M, Rahiotis C, Geis-Gerstorfer J. Marginal behaviour of self-etch adhesive/composite and combined amalgam-composite restorations. Eur J Prosthodont Restor Dent. 2010 Jun;18(2):70-7. PubMed PMID: 20698421.  66/66: Badr AE. Marginal adaptation and cytotoxicity of bone cement compared with amalgam and mineral trioxide aggregate as root-end filling materials. J Endod. 2010 Jun;36(6):1056-60. doi: 10.1016/j.joen.2010.02.018. Epub 2010 Apr 8. PubMed PMID: 20478465.  67/68: Roedig JJ, Shah J, Elayi CS, Miller CS. Interference of cardiac pacemaker and implantable cardioverter-defibrillator activity during electronic dental device use. J Am Dent Assoc. 2010 May;141(5):521-6. PubMed PMID: 20436099.  68/69: Cehreli SB, Arhun N, Celik C. Amalgam repair: quantitative evaluation of amalgam-resin and resin-tooth interfaces with different surface treatments. Oper Dent. 2010 May-Jun;35(3):337-44. doi: 10.2341/09-135-L. PubMed PMID: 20533635.  69/70: Roggenkamp CL, Berry FA, Lu H. In vitro bond strengths of amalgam added to existing amalgams. Oper Dent. 2010 May-Jun;35(3):314-23. doi: 10.2341/09-180-L. PubMed PMID: 20533632.  70/71: Suzuki P, de Souza V, Holland R, Murata SS, Gomes-Filho JE, Dezan Junior E, Rodrigues Dos Passos T. Tissue reaction of the EndoREZ in root canal fillings short of or beyond an apical foramenlike communication. Oral Surg Oral Med Oral Pathol Oral Radiol Endod. 2010 May;109(5):e94-9. doi: 10.1016/j.tripleo.2009.12.047. PubMed PMID: 20416527.  71/72: Dornelles Rde F, Cardim VL, Martins MT, Pinto AC, Alonso N. Spring-mediated skull expansion: overall effects in sutural and parasutural areas. An experimental study in rabbits. Acta Cir Bras. 2010 Apr;25(2):169-75. PubMed PMID: 20305884.  72/73: Machado DF, Bertassoni LE, Souza EM, Almeida JB, Rached RN. Effect of additives on the compressive strength and setting time of a Portland cement. Braz Oral Res. 2010 Apr-Jun;24(2):158-64. PubMed PMID: 20658033.  73/74: Hashemipour MA, Mohammadpour A, Nassab SA. Transient thermal and stress analysis of maxillary second premolar tooth using an exact three-dimensional model. Indian J Dent Res. 2010 Apr-Jun;21(2):158-64. doi: 10.4103/0970-9290.66624. PubMed PMID: 20657080.  74/75: Kim SY, Ferracane J, Kim HY, Lee IB. Real-time measurement of dentinal fluid flow during amalgam and composite restoration. J Dent. 2010 Apr;38(4):343-51. doi: 10.1016/j.jdent.2009.12.008. Epub 2010 Jan 7. PubMed PMID: 20060432.  75/76: Baliga MS, Bhat SS. Effect of fluorides from various restorative materials on remineralization of adjacent tooth: an in vitro study. J Indian Soc Pedod Prev  Dent. 2010 Apr-Jun;28(2):84-90. doi: 10.4103/0970-4388.66742. PubMed PMID: 20660973.  76/77: Hewlett S, Wadenya RO, Mante FK. Bond strength of luting cements to core foundation materials. Compend Contin Educ Dent. 2010 Mar;31(2):140-6. PubMed PMID: 20344899.  77/78: Imbery TA, Swigert R, Richman B, Sawicki V, Pace L, Moon PC. Resistance of composite and amalgam core foundations retained with and without pins and bonding agents. Gen Dent. 2010 Mar-Apr;58(2):130-7; quiz 138-9. PubMed PMID: 20236921.  78/79: Pereira HA, Iano FG, da Silva TL, de Oliveira RC, de Menezes ML, Buzalaf MA. Recovery of silver residues from dental amalgam. J Appl Oral Sci. 2010 Mar-Apr;18(2):121-6. PubMed PMID: 20485922.  79/80: Choi C, Driscoll CF, Romberg E. Comparison of cutting efficiencies between electric and air-turbine dental handpieces. J Prosthet Dent. 2010 Feb;103(2):101-7. doi: 10.1016/S0022-3913(10)60013-3. PubMed PMID: 20141814.  80/81: Post LK, Lima FG, Xavier CB, Demarco FF, Gerhardt-Oliveira M. Sealing ability of MTA and amalgam in different root-end preparations and resection bevel angles: an in vitro evaluation using marginal dye leakage. Braz Dent J. 2010;21(5):416-9. PubMed PMID: 21180797.  81/82: Braga MM, Chiarotti AP, Imparato JC, Mendes FM. Validity and reliability of methods for the detection of secondary caries around amalgam restorations in primary teeth. Braz Oral Res. 2010 Jan-Mar;24(1):102-7. PubMed PMID: 20339722.  82/83: Rodrigues Junior SA, Pin LF, Machado G, Della Bona A, Demarco FF. Influence of different restorative techniques on marginal seal of class II composite restorations. J Appl Oral Sci. 2010 Jan-Feb;18(1):37-43. PubMed PMID: 20379680.  83/84: George GN, Singh SP, Hoover J, Pickering IJ. The chemical forms of mercury in aged and fresh dental amalgam surfaces. Chem Res Toxicol. 2009 Nov;22(11):1761-4. doi: 10.1021/tx900309c. PubMed PMID: 19842619; PubMed Central PMCID: PMC2866173.  84/85: Shahidi SH, Bronoosh P, Alavi AA, Zamiri B, Sadeghi AR, Bagheri MH, Javadpour S. Effect of magnetic resonance imaging on microleakage of amalgam restorations: an in vitro study. Dentomaxillofac Radiol. 2009 Oct;38(7):470-4. doi: 10.1259/dmfr/30077669. PubMed PMID: 19767518.  85/86: Rajbaran S, Dannheimer M, De Wet F. The effect of thermocycling on the determination of microleakage in Permite amalgam restorations. SADJ. 2009 Oct;64(9):394-6. PubMed PMID: 20411833. |
