# Supplementary material for: Do Laboratory Results Concerning High-Viscosity Glass-Ionomers versus Amalgam for Tooth Restorations Indicate Similar Effect Direction and Magnitude than that of Controlled Clinical Trials? - A Meta-Epidemiological Study
Source: PLoS One. 2015 Jul 13;10(7):e0132246. doi: 10.1371/journal.pone.0132246 (PMC4500394; doi:10.1371/journal.pone.0132246)
Supplement: S1 PRISMA Flow Chart — (DOC) [file pone.0132246.s005.doc]

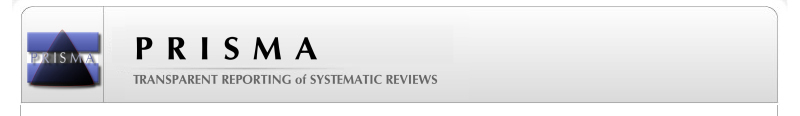
**PRISMA 2009 Flow Diagram**

**Screening**

**Included**

**Eligibility**

**Identification**

Records identified through database searching
(n = 25 517)

Additional records identified through other sources
(n = 6 )

Records after duplicates and non-relevant reports removed (n = 32 )

Records screened
(n = 32 )

Records excluded
(n = 15)

Records accepted eligibility (n = 17)

Datasets extracted and included in analysis
(n = 34)

Datasets related to laboratory trials
(n = 13 )

Datasets related to clinical controlled trials (n = 21 )
